# SARS-CoV-2-specific immune responses and clinical outcomes after COVID-19 vaccination in patients with immune-suppressive disease

**DOI:** 10.1038/s41591-023-02414-4

**Published:** 2023-07-06

**Authors:** Eleanor Barnes, Carl S. Goodyear, Michelle Willicombe, Charlotte Gaskell, Stefan Siebert, Thushan I de Silva, Sam M. Murray, Daniel Rea, John A. Snowden, Miles Carroll, Sarah Pirrie, Sarah J. Bowden, Susanna J. Dunachie, Alex Richter, Zixiang Lim, Jack Satsangi, Gordon Cook, Ann Pope, Ana Hughes, Molly Harrison, Sean H. Lim, Paul Miller, Paul Klenerman, PITCH consortium, Alex G. Richter, Alex Mentzer, Alexandra Deeks, Anni Jamsen, Anthony Brown, Chris Conlon, Chris Dold, Christopher J. A. Duncan, Donal Skelly, Barbara Kronsteiner, Priyanka Abraham, Eloise Phillips, Katie Jeffery, Lance Turtle, Lisa Frending, Lizzie Stafford, Mohammad Ali, Patpong Rongkard, Rebecca Payne, Sandra Adele, Simon Travis, Siobhan Gardiner, Sue L. Dobson, Tom Malone, Sagida Bibi, Miles Carroll, Sian Faustini, Sarah Foulkes, John Frater, Victoria Hall, Susan Hopkins, Jasmin Islam, Teresa Lambe, Stephanie Longet, Shona C. Moore, Ashley Otter, Sarah L. Rowland-Jones, James E. D. Thaventhir, Daniel G. Wootton, Neil Basu, Ashley Gilmour, Sophie Irwin, Georgina Meacham, Thomas Marjot, Stavros Dimitriadis, Peter Kelleher, Maria Prendecki, Candice Clarke, Paige Mortimer, Stacey McIntyre, Rachael Selby, Naomi Meardon, Dung Nguyen, Tom Tipton, Stephanie Longet, Stephen Laidlaw, Kim Orchard, Georgina Ireland, CONSENSUS, Kevin Brown, Gayatri Amirthalingam, David Thomas, Pamela Kearns, Amanda Kirkham, Iain B. McInnes, OCTAVE Collaborative Group, Richard Beesley, Vicky Churchill, Holly Loughton, Elspeth Insch, Eilean MacDonald, Gary Middleton, Lucinda Billingham, Faye Lowe, Sophia Magwaro, Saly Al-Taei, Maxine Arnott, Louise Bennett, James Brock, Victora Keillor, Andrew Melville, Lisa Melville, Samantha Miller, Aurelie Najm, Caron Paterson, Lewis Rodgers, Matthew Rutherford, Suzann Rundell, Emily Smith, Lynn Stewart, Flavia Sunzini, Andrew Tong, Kieran Woolcock, Faisal Basheer, Charles Crawley, Ram Malladi, Andrew King, Sophie Lockey, Ben Uttenthal, Mickey B. C. Koh, Sam Hansford, Gurjinder Sandhar, Murali Kesavan, Celia Moore, Pinelopi Manousou, Gareth Hahn, Benjamin Mullish, Maria Atta, Sarah Gleeson, Liz Lightstone, Paul Martin, Stephen McAdoo, Tina Thomson, Daniele Avenoso, Robin Sanderson, Claire Taylor, Khushpreet Bhandal, Diana Hull, Palak Trivedi, Andrew Filer, Erin Hurst, Amy Publicover, Katy Scouse, Jem Chalk, Daniel Hanke, Josef Hanke, Saoirse Healy, Nicholas Provine, Sarah Thomas, Victoria Walker, Zay Win, Doreen Trown, Patricia Faria, Julie Chackathayil, Clare Hutchison, Deborah Richardson

**Affiliations:** 1 Nuffield Department of Medicine, University of Oxford, Oxford, UK; 2 Oxford University Hospitals NHS Foundation Trust, Oxford, UK; 3 College of Medical, Veterinary & Life Sciences, University of Glasgow, Glasgow, UK; 4 Centre for Inflammatory Disease, Department of Immunology and Inflammation, Imperial College London, Hammersmith Campus, London, UK; 5 Cancer Research UK Clinical Trials Unit (CRCTU), University of Birmingham, Edgbaston, Birmingham, UK; 6 Department of Infection, Immunity and Cardiovascular Disease, The Medical School, The University of Sheffield, Sheffield, UK; 7 Department of Haematology, Sheffield Teaching Hospitals NHS Foundation Trust, Royal Hallamshire Hospital, Sheffield, UK; 8 Wellcome Centre for Human Genetics, University of Oxford, Oxford, UK; 9 Clinical Immunology Service, University of Birmingham, Edgbaston, Birmingham, UK; 10 National Institute for Health Research, Leeds MIC, University of Leeds, Leeds, UK; 11 Centre for Cancer Immunology, University of Southampton, Southampton, UK; 12 British Society of Blood and Marrow Transplantation and Cellular Therapy, Guy’s Hospital, London, UK; 13 Department of Infectious Diseases, Imperial College London, School of Medicine Chelsea and Westminster Hospital, London, UK; 14 Department of Haematology, University Hospital Southampton NHS Foundation Trust, Southampton, UK; 15 UK Health Security Agency (UKHSA), Immunisation and Vaccine Preventable Diseases Division, UK Health Security Agency, London, UK; 16 National Institute for Health Research Birmingham Biomedical Research Centre, Institute of Cancer and Genomic Sciences, University of Birmingham, Birmingham, UK; 17 Oxford Vaccine Group, Department of Paediatrics, University of Oxford, Oxford, UK; 18 Translational and Clinical Research Institute, Newcastle University, Newcastle, UK; 19 Nuffield Dept of Clinical Neurosciences, University of Oxford, Oxford, UK; 20 Institute of Infection, Veterinary and Ecological Sciences, University of Liverpool, Liverpool, UK; 21 Institute for Immunology and Immunotherapy, College of Medical and Dental Science, University of Birmingham, Birmingham, UK; 22 Immunisation and Vaccine Preventable Diseases Division, UK Health Security Agency, London, UK; 23 Department of Infection, Immunity and Cardiovascular Disease, University of Sheffield, Sheffield, UK; 24 MRC Toxicology Unit, University of Cambridge, Cambridge, UK; 25 Patient and Public Representatives on the Trial Management Group, Birmingham, UK; 26 Cancer Centre, University Hospitals Birmingham, NHS Foundation Trust, Birmingham, UK; 27 Clinical Immunology Service,University of Birmingham, Edgebaston, Birmingham, UK; 28 University of Glasgow, Glasgow, UK; 29 Department of Haematology, Cambridge University Hospitals NHS Foundation Trust, Cambridge, UK; 30 Infection and Immunity Clinical Academic Group, St. George’s, University of London; Department of Haematology, St. George’s University Hospital NHS Foundation Trust, London, UK; 31 Department of Oncology, Cancer and Haematology Centre, Churchill Hospital, Headington, Oxford, UK; 32 Division of Digestive Diseases, Department of Metabolism, Digestion and Reproduction, Faculty of Medicine, Imperial College London, London, UK; 33 Department of Haematology, Hammermith Hospital, London, UK; 34 Imperial College Healthcare NHS Trust, Hammersmith Hospital, London, UK; 35 King’s College Hospital NHS Foundation Trust, London, UK; 36 Leeds Institute of Medical Research, University of Leeds, Leeds, UK; 37 Liver Research Delivery Team, University Hospitals Birmingham NHS Foundation Trust, Birmingham, UK; 38 National Institute for Health Research (NIHR) Birmingham Biomedical Research Centre, Centre for Liver and Gastrointestinal Research, Institute of Immunology and Immunotherapy, University of Birmingham, Birmingham, UK; 39 National Institute for Health Research (NIHR) Birmingham Biomedical Research Centre and NIHR Clinical Research Facility, Institute of Inflammation and Ageing, University of Birmingham and University Hospitals Birmingham NHS Foundation Trust, Birmingham, UK; 40 Northern Centre for Cancer Care, Freeman Hospital, Newcastle upon Tyne, UK; 41 Sheffield Teaching Hospitals NHS Foundation Trust, Royal Hallamshire Hospital, Sheffield, UK; 42 St. George’s hospital and Medical School, St George’s University Hospitals NHS Foundation Trust, London, UK; 43 University Hospital Southampton NHS Foundation Trust, Southampton General Hospital, Southampton, UK

**Keywords:** Translational immunology, Risk factors

## Abstract

Severe acute respiratory syndrome coronavirus 2 (SARS-CoV-2) immune responses and infection outcomes were evaluated in 2,686 patients with varying immune-suppressive disease states after administration of two Coronavirus Disease 2019 (COVID-19) vaccines. Overall, 255 of 2,204 (12%) patients failed to develop anti-spike antibodies, with an additional 600 of 2,204 (27%) patients generating low levels (<380 AU ml^−1^). Vaccine failure rates were highest in ANCA-associated vasculitis on rituximab (21/29, 72%), hemodialysis on immunosuppressive therapy (6/30, 20%) and solid organ transplant recipients (20/81, 25% and 141/458, 31%). SARS-CoV-2-specific T cell responses were detected in 513 of 580 (88%) patients, with lower T cell magnitude or proportion in hemodialysis, allogeneic hematopoietic stem cell transplantation and liver transplant recipients (versus healthy controls). Humoral responses against Omicron (BA.1) were reduced, although cross-reactive T cell responses were sustained in all participants for whom these data were available. BNT162b2 was associated with higher antibody but lower cellular responses compared to ChAdOx1 nCoV-19 vaccination. We report 474 SARS-CoV-2 infection episodes, including 48 individuals with hospitalization or death from COVID-19. Decreased magnitude of both the serological and the T cell response was associated with severe COVID-19. Overall, we identified clinical phenotypes that may benefit from targeted COVID-19 therapeutic strategies.

## Abstract (web summary)

Serological analysis and infection outcomes of participants in the multi-center, prospectively enrolled OCTAVE cohort, comprising 2,686 participants with immune-suppressive diseases who recieved two COVID-19 vaccines, reveals specific clinical phenotypes that might benefit from specific COVID-19 therapeutic strategies.

## Main

The rapid development of vaccines against severe acute respiratory syndrome coronavirus 2 (SARS-CoV-2) has been hugely effective in the management of the Coronavirus Disease 2019 (COVID-19) pandemic^1,2^. National vaccination programs have shown that COVID-19 vaccines prevent wild-type SARS-CoV-2 infection and protect against severe disease from other SARS-CoV-2 variants, including Omicron^3^. However, volunteers in the original vaccine trials were healthy, without known chronic disease and not receiving immune-modifying treatments. In the United Kingdom (UK), in 2019, more than 60% of people aged over 65 years had one or more chronic disease, with more than 12 million people aged 18–65 years living with a chronic condition lasting more than 12 months^4^. UK government estimates suggest that 500,000 people have immune-suppressive diseases. Disease cohort studies^5^ and population studies using primary care health records^6^ showed that immune-suppressed patients are at increased risk of severe COVID-19 and death after SARS-CoV-2 infection in the pre-COVID-19 vaccine era. Many studies have shown suboptimal COVID-19 vaccine immune responses in cohorts of patients with chronic disease and in those receiving immune-suppressive therapy^7–23^. In general, these studies have focused on specific disease cohorts, and few have robustly evaluated cellular immune responses. Furthermore, vaccine responses against SARS-CoV-2 Omicron have been rarely assessed in specific patient cohorts^24,25^. Population studies including immune-suppressed patients have shown lower rates of SARS-CoV-2 spike antibody positivity after vaccination with only moderate vaccine effectiveness^26^ and have identified immune-suppressive disease after vaccination as a risk factor for severe COVID-19 and death^27,28^. Immune-suppressive disease remains a risk factor for severe outcomes with Omicron infection^29–31^, even though this variant appears less pathogenic, even when accounting for confounders, including vaccination status^32^.

In this prospective, multi-center study (Observational Cohort trial T cells, Antibodies and Vaccine Efficacy in SARS-CoV-2 (OCTAVE)), we evaluated functional humoral and T cell responses after COVID-19 vaccination, using centralized immune assays in patients receiving immune-suppressive therapy (for solid cancer, hematological malignancy, ANCA-associated vasculitis on rituximab, inflammatory arthritis, liver and kidney transplantation, autoimmune liver disease on immunosuppression, inflammatory bowel disease, ulcerative colitis and undefined inflammatory bowel disease); patients receiving autologous and allogeneic hematopoietic stem cell transplant (auto-HSCT and allo-HSCT); patients treated with chimeric antigen receptor (CAR) T cells; or patients with disease states known to modulate immune responses intrinsically (patients with end-stage kidney disease receiving hemodialysis with or without immune suppression and patients with advanced liver disease). Patients were vaccinated using mRNA (BNT162b2 or mRNA-1273) or ChAdOx1 nCoV-19 encoding ancestral SARS-CoV-2 spike antigens according to UK government-recommended vaccine schedules, and vaccine responses were evaluated before and after homologous first dose (V1) and second dose (V2) vaccination.

Patients were recruited for evaluation of SARS-CoV-2 serological responses 28 d after V2 with the magnitude of the T cell response assessed in a large subset of patients longitudinally (primary study endpoints). These responses were compared to a healthy control cohort matched by age, sex, prior SARS-CoV-2 infection and vaccine type, and the safety profile of vaccines in patient populations was assessed. Cellular and humoral responses were associated with SARS-CoV-2 infection events and COVID-19 disease severity. Exploratory endpoints included characterization of functional T cell and humoral responses and immune analysis in blood and saliva against variants of concern (VOCs). Using pairwise and regression analysis, we determined the contribution of disease phenotype, drug therapy and vaccine type to COVID-19 humoral and cellular vaccine responses, identifying patient subgroups that failed to seroconvert. Using uniform sampling timepoints and centralized immune assays, we directly compared COVID-19 vaccine immune responsiveness and infection outcomes among multiple disease phenotypes in immune-suppressive disease.

## Results

### Patient Demographics

**Extended Data Fig. 1:**
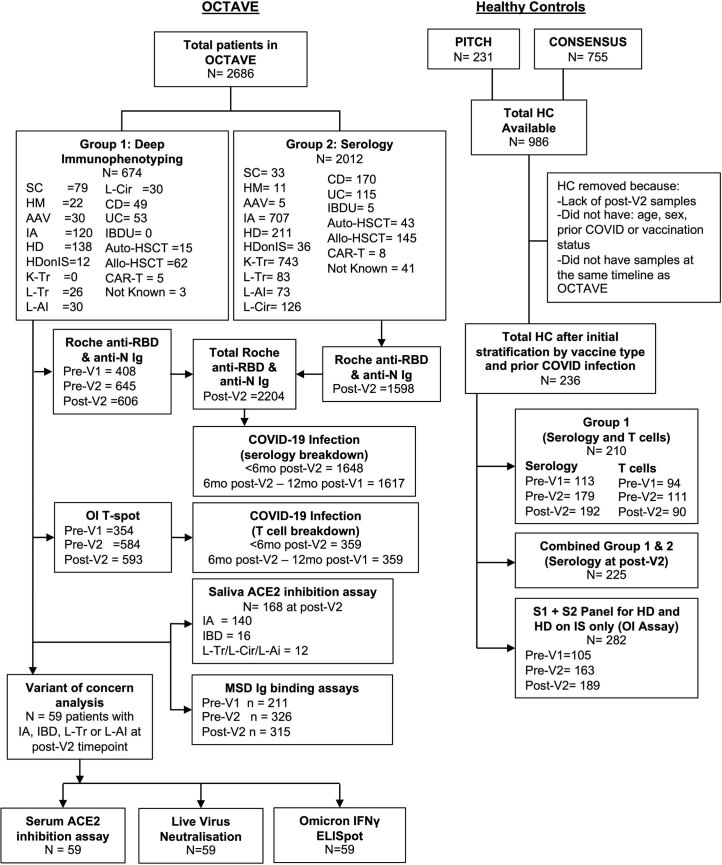
Recruitment and analysis overview of OCTAVE and healthy control groups. Recruitment and analysis overview of OCTAVE and HC participants. MSD = MesoScale Discovery, Ig = Immunoglobulin, S = Spike, RBD = Receptor binding domain, N = Nucleocapsid.

OCTAVE recruited 2,686 patients, including 2,012 for the evaluation of SARS-CoV-2 anti-receptor-binding domain (RBD) antibody responses (serology cohort) 28 d after V2 and 674 into a deep immunophenotyping cohort for the evaluation of T cell and humoral responses over time (Extended Data Fig. 1). In addition, 236 matched healthy control individuals (UK Health Security Agency (UKHSA) CONSENSUS and PITCH cohorts) were available for comparative analysis. Demographic data (available in 2,645 patients and 236 healthy controls) (Table 1) show that 1,430 of 2,881 (50%) patients were male, although distribution varied by disease cohort. Most patients, 2,629 of 2,881 (91%), were younger than 75 years of age; 2,038 of 2,881 (70%) reported White ethnicity; 479 of 2,881 (17%) reported Asian ethnicity; and 150 of 2,881 (5%) reported Black ethnicity. Previous SARS-CoV-2 infection (SARS-CoV-2 polymerase chain reaction (PCR) positive or anti-nucleocapsid or anti-spike antibody detected at baseline) was reported in 398 of 2,881 (14%) individuals, with higher rates in some disease cohorts (for example, hemodialysis in 104/211 (49%)). Of 2,881 participants (44%), 1,249 had overweight or obesity, and 567 (20%) had type 1 or type 2 diabetes. Of 2,881 participants, 1,876 (65%) received ChAdOx1 nCoV-19, and 975 (34%) received BNT162b2. Three participants received mRNA-1273 (in 27 individuals, the vaccine type was unknown).

**Table 1:** Patient characteristics presented for patients in the deep immunophenotyping and serology groups

	HC (236)		SC (112)		HM (33)		AAV (35)		IA (707)		HD (211)		HD on IS (36)		K-Tr (743)		L-Tr (83)		L-AI (73)		L-Cir (126)		CD (170)		UC (115)		IBD-U (5)		Auto-HSCT (43)		Allo-HSCT (145)		CAR-T (8)		Total (2,881)	
Sex																																				
Male	104	(44%)	9	(8%)	21	(64%)	19	(54%)	236	(33%)	119	(56%)	20	(56%)	474	(64%)	52	(63%)	24	(33%)	68	(54%)	97	(57%)	70	(61%)	3	(60%)	26	(60%)	83	(57%)	5	(63%)	1430	(50%)
Female	132	(56%)	103	(92%)	12	(36%)	16	(46%)	470	(66%)	92	(44%)	16	(44%)	269	(36%)	31	(37%)	49	(67%)	58	(46%)	73	(43%)	45	(39%)	2	(40%)	17	(40%)	62	(43%)	3	(38%)	1450	(50%)
Unknown	0	(0%)	0	(0%)	0	(0%)	0	(0%)	1	(0%)	0	(0%)	0	(0%)	0	(0%)	0	(0%)	0	(0%)	0	(0%)	0	(0%)	0	(0%)	0	(0%)	0	(0%)	0	(0%)	0	(0%)	1	(0%)
Age (years)																																				
15–44	22	(9%)	24	(21%)	2	(6%)	10	(29%)	130	(18%)	18	(9%)	5	(14%)	153	(21%)	17	(20%)	10	(14%)	8	(6%)	113	(66%)	69	(60%)	5	(100%)	4	(9%)	42	(29%)	4	(50%)	636	(22%)
45–64	63	(27%)	55	(49%)	19	(58%)	17	(49%)	393	(56%)	61	(29%)	19	(53%)	361	(49%)	39	(47%)	39	(53%)	65	(52%)	54	(32%)	38	(33%)	0	(0%)	22	(51%)	72	(50%)	4	(50%)	1321	(46%)
65–74	101	(43%)	24	(21%)	9	(27%)	4	(11%)	152	(21%)	58	(27%)	9	(25%)	172	(23%)	23	(28%)	19	(26%)	45	(36%)	3	(2%)	6	(5%)	0	(0%)	16	(37%)	30	(21%)	0	(0%)	671	(23%)
75+	50	(21%)	9	(8%)	3	(9%)	4	(11%)	32	(5%)	73	(35%)	3	(8%)	57	(8%)	4	(5%)	5	(7%)	8	(6%)	0	(0%)	2	(2%)	0	(0%)	1	(2%)	1	(1%)	0	(0%)	252	(9%)
Unknown	0	(0%)	0	(0%)	0	(0%)	0	(0%)	0	(0%)	1	(0%)	0	(0%)	0	(0%)	0	(0%)	0	(0%)	0	(0%)	0	(0%)	0	(0%)	0	(0%)	0	(0%)	0	(0%)	0	(0%)	1	(0%)
Ethnicity																																				
White	193	(82%)	84	(75%)	26	(79%)	28	(80%)	625	(88%)	50	(24%)	14	(39%)	319	(43%)	76	(92%)	61	(84%)	116	(92%)	159	(94%)	104	(90%)	5	(100%)	40	(93%)	131	(90%)	7	(88%)	2038	(71%)
Black	11	(5%)	7	(6%)	2	(6%)	0	(0%)	4	(1%)	54	(26%)	6	(17%)	59	(8%)	2	(2%)	0	(0%)	1	(1%)	1	(1%)	1	(1%)	0	(0%)	1	(2%)	1	(1%)	0	(0%)	150	(5%)
Asian	16	(7%)	1	(1%)	2	(6%)	1	(3%)	10	(1%)	96	(45%)	14	(39%)	308	(41%)	1	(1%)	10	(14%)	4	(3%)	7	(4%)	5	(4%)	0	(0%)	1	(2%)	2	(1%)	1	(13%)	479	(17%)
Mixed/Other	12	(5%)	17	(15%)	1	(3%)	2	(6%)	20	(3%)	9	(4%)	1	(3%)	36	(5%)	3	(4%)	2	(3%)	3	(2%)	3	(2%)	3	(3%)	0	(0%)	0	(0%)	4	(3%)	0	(0%)	116	(4%)
Not known	4	(2%)	3	(3%)	2	(6%)	4	(11%)	48	(7%)	2	(1%)	1	(3%)	21	(3%)	1	(1%)	0	(0%)	2	(2%)	0	(0%)	2	(2%)	0	(0%)	1	(2%)	7	(5%)	0	(0%)	98	(3%)
BMI																																				
Underweight	1	(0%)	1	(1%)	0	(0%)	0	(0%)	10	(1%)	8	(4%)	1	(3%)	3	(0%)	0	(0%)	3	(4%)	0	(0%)	6	(4%)	4	(3%)	0	(0%)	0	(0%)	6	(4%)	1	(13%)	44	(2%)
Healthy weight	14	(6%)	31	(28%)	11	(33%)	7	(20%)	163	(23%)	35	(17%)	4	(11%)	62	(8%)	30	(36%)	24	(33%)	25	(20%)	92	(54%)	54	(47%)	5	(100%)	15	(35%)	57	(39%)	2	(25%)	631	(22%)
Overweight	7	(3%)	20	(18%)	10	(30%)	8	(23%)	261	(37%)	32	(15%)	4	(11%)	81	(11%)	26	(31%)	28	(38%)	34	(27%)	39	(23%)	39	(34%)	0	(0%)	14	(33%)	42	(29%)	4	(50%)	649	(23%)
Obese	1	(0%)	28	(25%)	10	(30%)	15	(43%)	257	(36%)	42	(20%)	4	(11%)	71	(10%)	26	(31%)	14	(19%)	60	(48%)	28	(16%)	14	(12%)	0	(0%)	10	(23%)	20	(14%)	0	(0%)	600	(21%)
Unknown	5	(2%)	32	(29%)	2	(6%)	5	(14%)	16	(2%)	94	(45%)	23	(64%)	526	(71%)	1	(1%)	4	(5%)	7	(6%)	5	(3%)	4	(3%)	0	(0%)	4	(9%)	20	(14%)	1	(13%)	749	(26%)
Data unavailable	208	(88%)	0	(0%)	0	(0%)	0	(0%)	0	(0%)	0	(0%)	0	(0%)	0	(0%)	0	(0%)	0	(0%)	0	(0%)	0	(0%)	0	(0%)	0	(0%)	0	(0%)	0	(0%)	0	(0%)	208	(7%)
Prior COVID																																				
No confirmed infection	198	(84%)	95	(85%)	30	(91%)	33	(94%)	637	(90%)	106	(50%)	23	(64%)	692	(93%)	76	(92%)	69	(95%)	111	(88%)	154	(91%)	101	(88%)	5	(100%)	36	(84%)	112	(77%)	5	(63%)	2483	(86%)
Yes	38	(16%)	17	(15%)	3	(9%)	2	(6%)	70	(10%)	105	(50%)	13	(36%)	51	(7%)	7	(8%)	4	(5%)	15	(12%)	16	(9%)	14	(12%)	0	(0%)	7	(16%)	33	(23%)	3	(38%)	398	(14%)
Vaccine type																																				
ChAdOx nCoV-19	156	(66%)	44	(39%)	19	(58%)	33	(94%)	591	(84%)	127	(60%)	29	(81%)	326	(44%)	62	(75%)	52	(71%)	93	(74%)	140	(82%)	99	(86%)	3	(60%)	37	(86%)	61	(42%)	4	(50%)	1876	(65%)
BNT162b2	80	(34%)	61	(54%)	13	(39%)	2	(6%)	116	(16%)	84	(40%)	7	(19%)	410	(55%)	21	(25%)	21	(29%)	33	(26%)	30	(18%)	16	(14%)	2	(40%)	5	(12%)	70	(48%)	4	(50%)	975	(34%)
mRNA1273	0	(0%)	1	(1%)	0	(0%)	0	(0%)	0	(0%)	0	(0%)	0	(0%)	0	(0%)	0	(0%)	0	(0%)	0	(0%)	0	(0%)	0	(0%)	0	(0%)	0	(0%)	2	(1%)	0	(0%)	3	(0%)
Unknown	0	(0%)	6	(5%)	1	(3%)	0	(0%)	0	(0%)	0	(0%)	0	(0%)	7	(1%)	0	(0%)	0	(0%)	0	(0%)	0	(0%)	0	(0%)	0	(0%)	1	(2%)	12	(8%)	0	(0%)	27	(1%)
Smoking status																																				
Never smoked	20	(8%)	26	(23%)	14	(42%)	15	(43%)	369	(52%)	41	(19%)	7	(19%)	163	(22%)	45	(54%)	34	(47%)	40	(32%)	96	(56%)	69	(60%)	4	(80%)	7	(16%)	47	(32%)	6	(75%)	1003	(35%)
Previous smoker	1	(0%)	18	(16%)	2	(6%)	8	(23%)	255	(36%)	17	(8%)	2	(6%)	37	(5%)	28	(34%)	25	(34%)	55	(44%)	49	(29%)	30	(26%)	0	(0%)	4	(9%)	27	(19%)	1	(13%)	559	(19%)
Current smoker	0	(0%)	7	(6%)	0	(0%)	4	(11%)	72	(10%)	12	(6%)	0	(0%)	10	(1%)	2	(2%)	10	(14%)	23	(18%)	23	(14%)	13	(11%)	1	(20%)	0	(0%)	4	(3%)	0	(0%)	181	(6%)
Unknown	7	(3%)	61	(54%)	17	(52%)	8	(23%)	11	(2%)	141	(67%)	27	(75%)	533	(72%)	8	(10%)	4	(5%)	8	(6%)	2	(1%)	3	(3%)	0	(0%)	32	(74%)	67	(46%)	1	(13%)	930	(32%)
Data unavailable	208	(88%)	0	(0%)	0	(0%)	0	(0%)	0	(0%)	0	(0%)	0	(0%)	0	(0%)	0	(0%)	0	(0%)	0	(0%)	0	(0%)	0	(0%)	0	(0%)	0	(0%)	0	(0%)	0	(0%)	208	(7%)
Diabetes																																				
No	24	(10%)	101	(90%)	31	(94%)	27	(77%)	641	(91%)	107	(51%)	22	(61%)	473	(64%)	66	(80%)	59	(81%)	71	(56%)	165	(97%)	113	(98%)	5	(100%)	37	(86%)	138	(95%)	8	(100%)	2088	(72%)
Yes	0	(0%)	6	(5%)	2	(6%)	7	(20%)	62	(9%)	104	(49%)	14	(39%)	269	(36%)	17	(20%)	14	(19%)	55	(44%)	5	(3%)	2	(2%)	0	(0%)	4	(9%)	6	(4%)	0	(0%)	567	(20%)
Not known	4	(2%)	5	(4%)	0	(0%)	1	(3%)	4	(1%)	0	(0%)	0	(0%)	1	(0%)	0	(0%)	0	(0%)	0	(0%)	0	(0%)	0	(0%)	0	(0%)	2	(5%)	1	(1%)	0	(0%)	18	(1%)
Data unavailable	208	(88%)	0	(0%)	0	(0%)	0	(0%)	0	(0%)	0	(0%)	0	(0%)	0	(0%)	0	(0%)	0	(0%)	0	(0%)	0	(0%)	0	(0%)	0	(0%)	0	(0%)	0	(0%)	0	(0%)	208	(7%)

Reported as *n* (%).

ANCA-associated vasculitis; CD, Crohn’s disease; HC, healthy controls; HD, hemodialysis; HD on IS, hemodialysis on immunosuppression; HM, hemotological malignancy; IA, inflammatory arthritis; IBD-U, undefined inflammatory bowel disease; K-Tr, kidney transplant; L-AI, autoimmune hepatitis; L-Cir, liver cirrhosis; L-Tr, liver transplant; SC, solid cancer; UC, ulcerative colitis.

### COVID-19 serology after two vaccines

**Fig. 1:**
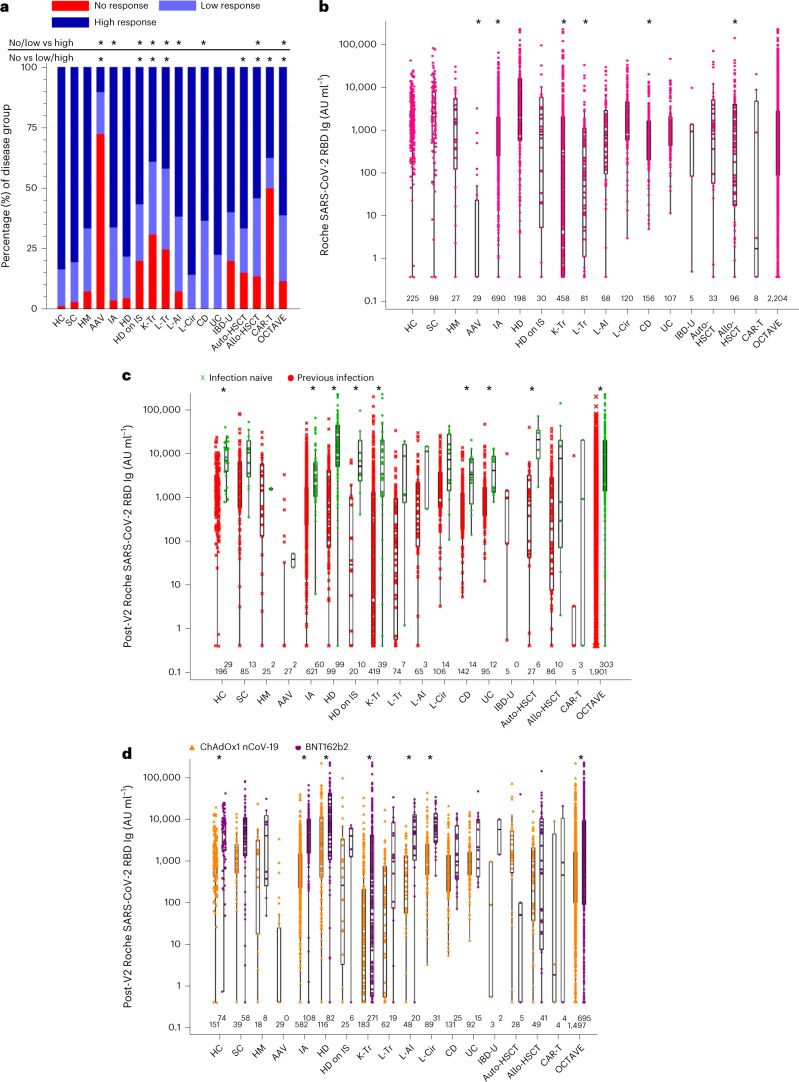
Anti-SARS-CoV-2 RBD total Ig responses in whole OCTAVE cohort at post-V2 timepoint. **a**, Proportion of group 1 and group 2 non (<0.8 AU ml^−1^), low (<380 AU ml^−1^) and high (>380 AU ml^−1^) anti-SARS-CoV-2 spike RBD total Ig responses. Statistical comparisons of the proportion of low and no versus high response and no versus low and high response in disease groups compared to healthy controls are presented. **b**, Magnitude of serological response in disease groups and healthy controls. Statistical comparisons comparing disease group to healthy controls are presented. **c**, Anti-SARS-CoV-2 spike RBD total Ig responses comparing previously infected with infection-naive patents. Statistical comparison of infection-naive individuals and previously infected individuals within each group is presented. **d**, Anti-SARS-CoV-2 spike RBD total Ig responses separated by vaccine type. Statistical comparison of vaccine type in each disease group is presented. Unpaired statistical comparison was made on all groups using a two-sided Kruskal–Wallis with post hoc Dunn’s testing. Comparisons of proportions were performed using χ^2^ or Fisher’s exact tests adjusted for significance using Bonferroni correction (adjusted alpha = 0.003). Only significant comparisons are presented. * indicates statistically significant by Bonferroni-adjusted alpha. Boxes represent median and IQR; whiskers represent ±1.5× IQR. AAV, ANCA-associated vasculitis; CD, Crohn’s disease; HC, healthy controls; HD, hemodialysis; HD on IS, hemodialysis on immunosuppression; HM, hemotological malignancy; IA, inflammatory arthritis; L-AI, autoimmune hepatitis; L-Cir, liver cirrhosis; L-Tr, liver transplant; SC, solid cancer; UC, ulcerative colitis.

Immunogenicity after two COVID-19 vaccines was evaluable in 2,204 patients and 225 matched healthy controls (Table 1). We assessed the rate of seropositivity (anti-RBD antibody titer ≥ 0.8 AU ml^−1^) after V2 (Fig. 1a): in healthy controls, 222 of 225 (99%) were seropositive compared to 1,949 of 2,204 (88%) patients (Fisher’s exact test, *P* < 0.001) (Fig. 1a and Supplementary Table 1). Compared to the healthy control group, there was a decrease in rates of seropositivity in ANCA-associated vasculitis (8/29, 28%), hemodialysis on immunosuppression (24/30, 80%), kidney transplant (317/458, 69%), liver transplant (61/81, 75%), auto-HSCT (28/33, 85%), allo-HSCT (83/96, 86%) and CAR-T (4/8, 50%) disease groups (*P* < 0.003, Bonferroni-adjusted alpha) (Fig. 1a and Supplementary Table 1). All other groups had a similar rate of seropositivity to healthy controls (cirrhosis, Crohn’s disease and ulcerative colitis, 100% seropositive rate).

Compared to the healthy control group, the median anti-RBD titers after V2 were decreased in the ANCA-associated vasculitis (*z* = 8.42, *P* < 0.001), inflammatory arthritis (*z* = 4.92, *P* < 0.001), kidney transplant (*z* = 10.58, *P* < 0.001), liver transplant (*z* = 6.82, *P* < 0.001), Crohn’s disease (*z* = 4.32, *P* = 0.001) and allo-HSCT (*z* = 4.18, *P* = 0.002) cohorts; other disease groups with enough participants to be included in the analysis did not differ from the healthy control group (Fig. 1b and Supplementary Table 2).

Low serological response threshold was defined by assessing anti-RBD Ig responses in healthy controls and identifying the upper value of the lowest decile (<380 AU ml^−1^) (Fig. 1b). There was a greater number of low responders across the entire disease cohort compared to the healthy control group (Fig. 1a). Combining no-responders and low-responders, there were significantly more patients in ANCA-associated vasculitis (Fisher’s exact *P* < 0.001), inflammatory arthritis (χ^2^ = 24.48, *P* < 0.001), hemodialysis on immunosuppression (χ^2^ = 12.14, *P* < 0.001), kidney transplant (χ^2^ = 120.03, *P* < 0.001), liver transplant (χ^2^ = 51.70, *P* < 0.001), autoimmune liver disease (χ^2^ = 14.69, *P* < 0.001), Crohn’s disease (χ^2^ = 20.02, *P* < 0.001) and allo-HSCT (χ^2^ = 30.81, *P* < 0.001) groups compared to the healthy control group (Fig. 1b and Supplementary Table 3).

We examined the effect of prior SARS-CoV-2 infection on anti-RBD Ig titers after V2 in the healthy control and disease groups (Fig. 1c and Supplementary Table 4). In those previously infected, anti-RBD Ig titers were significantly increased in healthy controls (*P* < 0.0001) and in the total OCTAVE cohort (*P* < 0.0001). Within disease groups, patients with previous infection and inflammatory arthritis (*P* < 0.0001), hemodialysis (*P* < 0.0001), hemodialysis on immunosuppression (*P* = 0.0002), kidney transplant (*P* < 0.0001), Crohn’s disease (*P* = 0.0006), ulcerative colitis (*P* = 0.0014) or auto-HSCT (*P* = 0.0004) had higher titers than SARS-CoV-2-naive patients (Supplementary Table 4).

Median anti-RBD Ig titer was significantly higher in patients who received two doses of BNT162b2 (*n* = 695) compared to two doses of ChAdOx1 nCoV-19 (*n* = 1,497) (*P* < 0.0001) and within the solid cancer (*P* = 0.0004), inflammatory arthritis (*P* < 0.0001), kidney transplant (*P* < 0.0001), autoimmune liver disease (*P* < 0.0001) and cirrhosis (*P* < 0.0001) individual disease groups (Fig. 1d and Supplementary Table 5). This was also seen in the healthy control group.

### SARS-CoV-2 spike and common cold coronavirus immunoglobulins

**Fig. 2:**
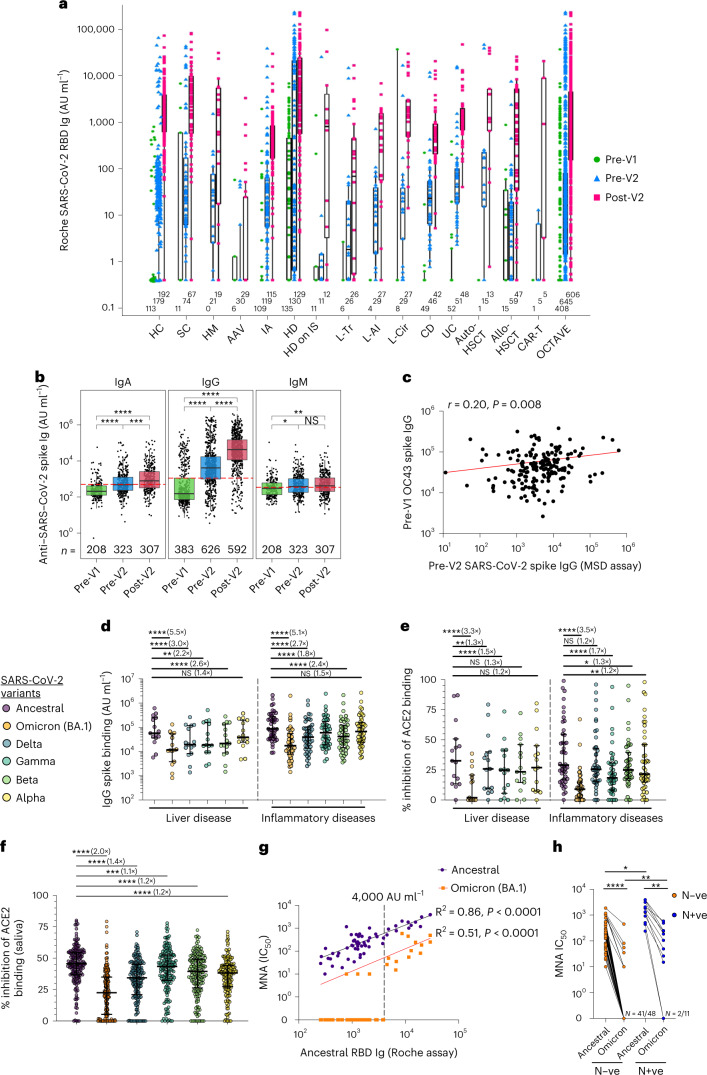
Serological responses to CCCs and SARS-CoV-2 VOCs after vaccination. **a**, Anti-SARS-CoV-2 RBD-binding total Ig before first vaccine (pre-V1) and before (pre-V2) and after (post-V2) second vaccine in group 1 participants. **b**, Anti-SARS-CoV-2 spike binding IgG, IgM and IgA assessed at all available timepoints. IgG was assessed in all group 1 participants; IgM and IgA were assessed in group 1 participants in the UC, CD, L-Tr, L-AI, L-Cir, IA and ANCA-associated vasculitis disease groups. Lines indicate threshold for seropositivity. **c**, Spearman’s correlation of anti-HCoV-OC43 spike IgG at pre-V1 compared to pre-V2 anti-SARS-CoV-2 full-spike IgG assessed in all group 1 participants. **d**, Serum IgG binding to SARS-CoV-2 VOC spike at post-V2 timepoint. **e**,**f**, Inhibition of SARS-CoV-2 VOC spike binding to hACE2 by participant serum (**e**) or saliva (**f**) at post-V2 timepoint. **g**,**h**, Microneutralization of live ancestral or omicron BA.1 SARS-CoV-2 at the post-V2 timepoint (**h**). Correlation of microneutralization IC_50_ with ancestral anti-SARS-CoV-2 RBD-binding total Ig (**g**) and microneutralization IC_50_ separated by previous SARS-CoV-2 infection status. **d**,**e**,**g**,**h**, *n* = 59 participants selected from liver and inflammatory disease groups with anti-SARS-CoV-2 RBD total Ig above 250 AU ml^−1^. **f**, *n* = 168 participants selected from inflammatory and liver disease groups. **d**–**f**, Lines represent median and IQR. Paired statistical comparisons among multiple groups (**d**–**f**,**h**) were assessed using two-sided Friedman’s test with Dunn’s correction or Wilcoxon’s rank-sum test with Bonferroni correction. Unpaired statistical comparisons among multiple groups were assessed using two-sided Mann–Whitney *U*-test with Bonferroni correction. *P < 0.05, **P < 0.01, ***P < 0.001, ****P < 0.0001. AAV, ANCA-associated vasculitis; CD, Crohn’s disease; HC, healthy controls; HD, hemodialysis; HD on IS, hemodialysis on immunosuppression; HM, hematological malignancy; IA, inflammatory arthritis; L-AI, autoimmune hepatitis; L-Cir, liver cirrhosis; L-Tr, liver transplant; MNA, microneutralization; Nucleocapsid negative, N-ve; Nucleocapsid positive, N+ve; NS, not significant; SC, solid cancer; V1, COVID-19 vaccine dose 1; V2, COVID-19 vaccine dose 2; UC, ulcerative colitis.

**Extended Data Fig. 2:**
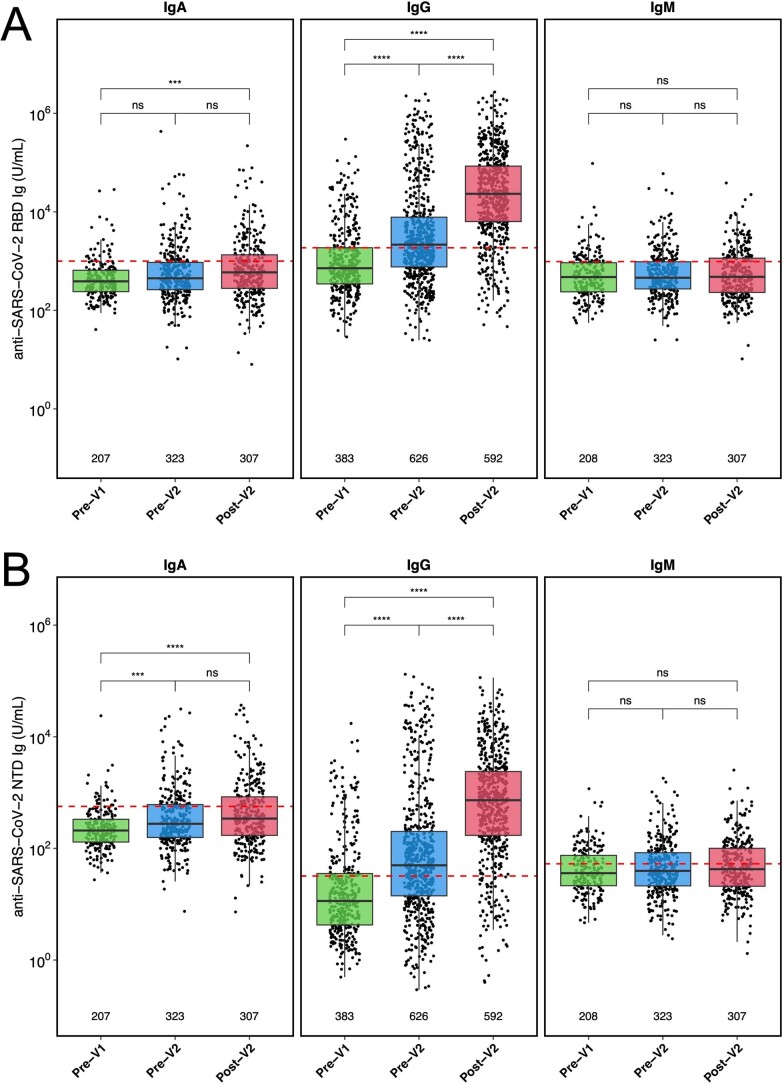
Binding IgA, IgG and IgM to SARS-CoV-2 Spike receptor binding domain (RBD) and N- terminal domain (NTD) measured by MSD assay. Binding IgA, IgG and IgM to SARS-CoV-2 Spike (**a**) receptor binding domain (RBD) and (**b**) N-terminal domain (NTD) measured by MSD assay. IgG in all patients in group 1 at all available timepoints, IgA and IgM in all timepoints from all group 1 participants in the IA, AAV, IBD, L-Tr, L-AI and L-Cir disease groups at the pre-V1 (Pre-V1), pre-V2 (Pre-V2) and post-V2 (Post V2) timepoints. Two-sided Mann-Whitney U test corrected for multiple comparisons by Bonferroni correction. * = adjusted P value < 0.05, ** adjusted P value < 0.01, ****adjusted P value < 0.0001. Boxes represent median and IQR, whiskers represent +/- 1.5x IQR.

**Extended Data Fig. 3:**
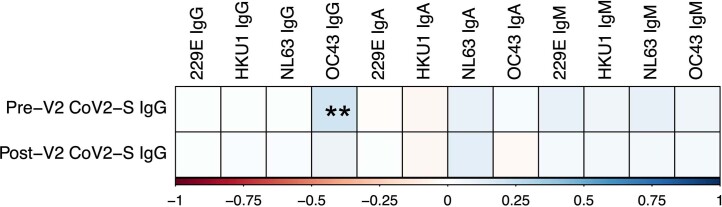
Correlation of vaccine induced IgG with baseline common cold coronavirus antibodies. Correlation plot of pre first vaccine (Pre-V2) and post second vaccine (Post-V2) anti- SARS-CoV-2 Spike binding IgG (MSD Assay) against baseline (Pre-V1) common cold coronavirus spike IgG, IgA and IgM antibodies. IgG data collected at baseline, Pre-V2 and Post-V2 in all participants in group 1. IgM and IgA collected in all group 1 participants in the UC, CD, L-Tr, L-AI, L-Cir, IA and AAV disease groups. Includes HCoV’s – 229E, HKU1, NL63 and OC43. Pearson correlation. *P < 0.05, **P < 0.01.

Serological and cellular immune responses were evaluated before V1, immediately before V2 and 28 d after V2 in 674 patients and in healthy controls matched by age, sex, prior SARS-CoV-2 and vaccine type (Supplementary Table 6). Median anti-RBD Ig titers were lower before V2 in ANCA-associated vasculitis, hemodialysis on immunosuppression, liver transplant, allo-HSCT and CAR-T groups compared to healthy controls (Fig. 2a and Supplementary Table 7) but increased after a second COVID-19 vaccine in all disease groups other than ANCA-associated vasculitis. Spike, RBD and N-terminal domain (NTD) IgG and spike IgA responses significantly increased after both one vaccine dose (*P* < 0.0001) and two vaccine doses (IgG *P* < 0.0001 and IgA *P* = 0.0003). Spike IgM and NTD IgA increased significantly only after the first dose (Fig. 2b). IgM responses to RBD and NTD did not increase after either dose (Extended Data Fig. 2a,b). We correlated IgG/IgA/IgM to each common cold coronavirus (CCC) spike protein at baseline with SARS-CoV-2 spike IgG after one and two vaccines in seronegative anti-nucleocapsid IgG patients: only IgG to HCoV-OC43 showed a weak positive correlation after the first vaccine (Fig. 2c and Extended Data Fig. 3).

### Serological responses to SARS-CoV-2 VoC in blood and saliva

**Extended Data Fig. 4:**
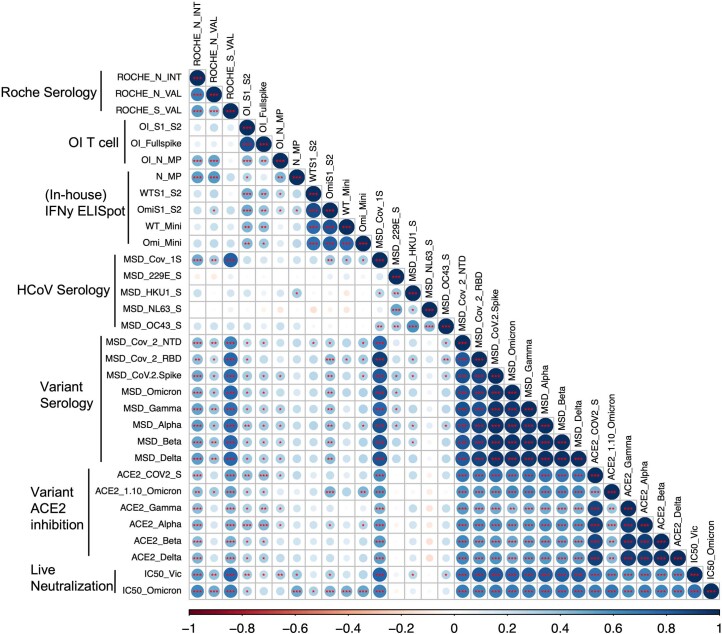
Correlation of post- second vaccine immune assay responses. Correlation plot of all assays used to evaluate serological and cellular responses on the 59 group 1 participants with deep immunophenotyping at the Post-V2 timepoint. Spearman rank sum test used. R^2^ depicted by area of circle and colour legend. * P value < 0.05; ** P value < 0.01, ***; P value < 0.001.

**Extended Data Fig. 5:**
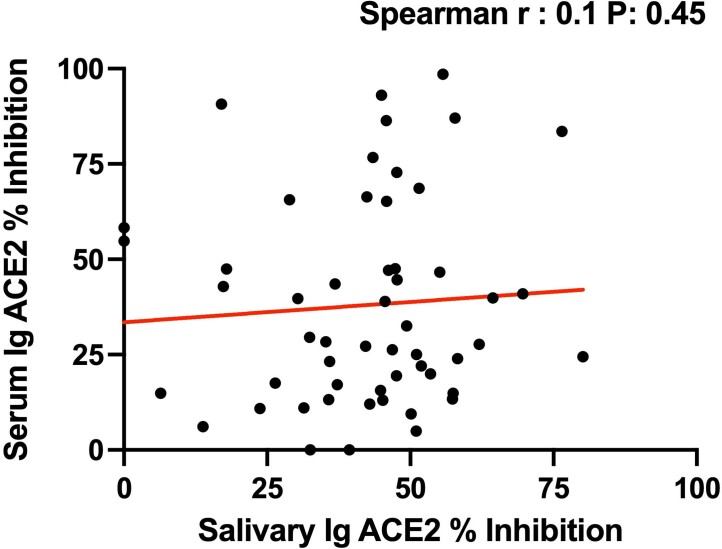
Correlation of serum and salivary inhibition of ACE2 binding. Spearman rank sum correlation of % inhibition of ACE2 by serum and saliva Ig at the post-V2 in immunocompromised individuals (n = 54).

We assessed the cross-reactivity of SARS-CoV-2 spike ancestral antibody responses to VOCs (Alpha (B.1.1.7), Beta (B.1.351), Gamma (P.1), Delta (B.1.617.2) and Omicron (B.1.1.529 and BA.1)) in 59 patients from the liver transplant, autoimmune liver disease, cirrhosis and inflammatory arthritis cohorts, representing a range of low to high anti-RBD Ig titers (Roche assay, range: 257–29,332 AU ml^−1^) after V2. Compared to ancestral, median spike IgG and spike–ACE-2 binding was significantly decreased to all VOCs except Alpha in all disease groups but most notably to Omicron BA.1 (Fig. 2d,e). Binding to ancestral and each VOC spike correlated with post-V2 anti-RBD antibody titer (Extended Data Fig. 4). Salivary Ig inhibited ancestral spike ACE2 binding with 80% efficiency (Fig. 2f) but was reduced against all VOCs. Inhibition of ACE2 binding in saliva and serum did not correlate (Extended Data Fig. 5). In live microneutralization assays, all patients neutralized ancestral SARS-CoV-2 (mean half-maximal inhibitory concentration (IC_50_) = 589), but there was a 13-fold decrease in the neutralization Omicron BA.1 (mean IC_50_ = 44). Only 27% of patients could neutralize Omicron BA.1 (Fig. 2g,h). There was a significant positive correlation between anti-RBD Ig titers and ancestral and Omicron BA.1 neutralization (Fig. 2g). Notably, those with a Roche anti-RBD Ig titer of <4,000 AU ml^−1^ were largely unable to neutralize Omicron. Patients with previous SARS-CoV-2 infection had significantly higher microneutralization IC_50_ than naive patients (Fig. 2h), with a higher proportion able to neutralize Omicron (9/11 versus 7/48, *P* < 0.0001, Fisher’s exact test).

### Cellular immune responses after vaccination

**Fig. 3:**
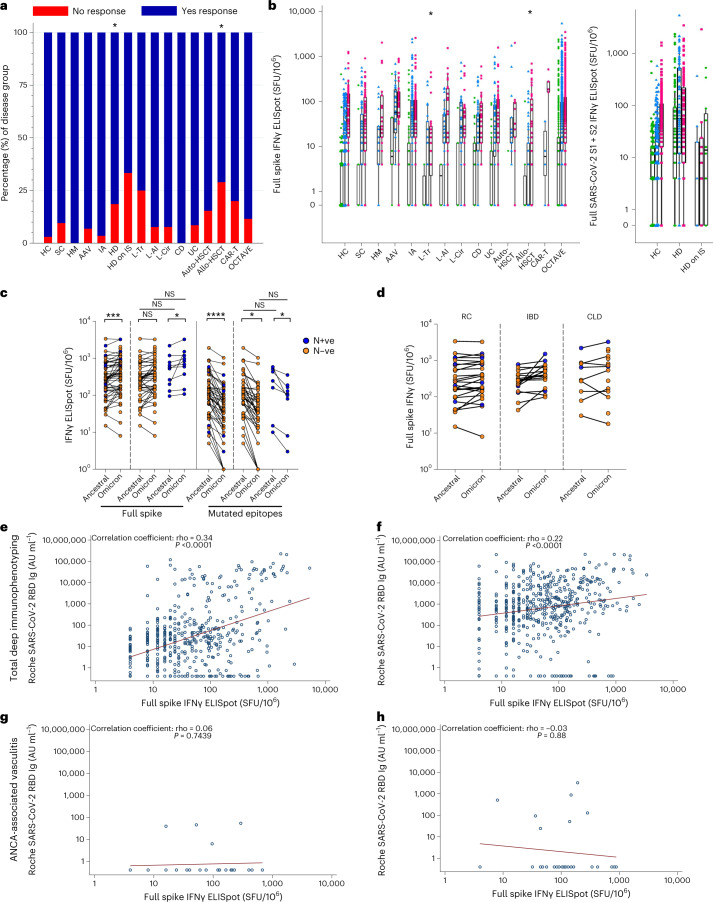
T cell responses to ancestral and Omicron BA.1 SARS-CoV-2 after vaccination. **a**,**b**, IFNγ T cell response to SARS-CoV-2 spike measured by Oxford Immunotec assay presented as the proportion of individuals with or without an anti-SARS-CoV-2 spike T cell response (**a**) and the magnitude of IFNγ T cell response in disease groups (*n* = 645) and healthy controls (*n* = 189) (**b**). **a**,**b**, The statistical comparison presented is disease group compared to healthy controls (HC) in all participants in group 1. **c**,**d**, IFNγ T cell response to ancestral and Omicron BA.1 spike or pools of peptides covering regions mutated in BA.1 and their ancestral equivalents, measured by in-house IFNγ ELISpot at post-V2 timepoint (*n* = 59 participants selected from liver, rheumatic and inflammatory disease cohorts). **e**–**h**, Selected examples of the correlation of anti-SARS-CoV-2 RBD binding total Ig with IFNγ T cell response to ancestral SARS-CoV-2 spike at pre-V2 (**e**,**g**) and post-V2 (**f**,**h**) timepoints in group 1 (all disease groups) (**e**,**f**) and ANCA-associated vasculitis on rituximab patients (**g**,**h**). Unpaired statistical comparisons (**b**,**c**,**d**) were assessed with a Kruskal–Wallis test with post hoc Dunn’s testing (adjusted alpha = 0.003). Paired statistical tests were performed with two-sided Wilcoxon’s rank-sum test with Bonferroni correction (adjusted alpha = 0.0125). **a**–**c**, * indicates statistically significant by Bonferroni-adjusted alpha. ***adjusted *P* < 0.001, ****adjusted *P* < 0.0001. **e**–**h**, Correlations are Spearman’s rank-sum correlation, and fitted line is presented. **b**, Boxes represent median and IQR; whiskers represent ±1.5× IQR. AAV, ANCA-associated vasculitis; CD, Crohn’s disease; CLD, chronic liver disease; HD, hemodialysis; HD on IS, hemodialysis on immunosuppression; HM, hematological malignancy; IA, inflammatory arthritis; L-AI, autoimmune hepatitis; L-Cir, liver cirrhosis; L-Tr, liver transplant; Nucleocapsid negative, N-ve; Nucleocapsid positive, N+ve; NS, not significant; RC, rheumatic conditions; SC, solid cancer; UC, ulcerative colitis.

**Extended Data Fig. 6:**
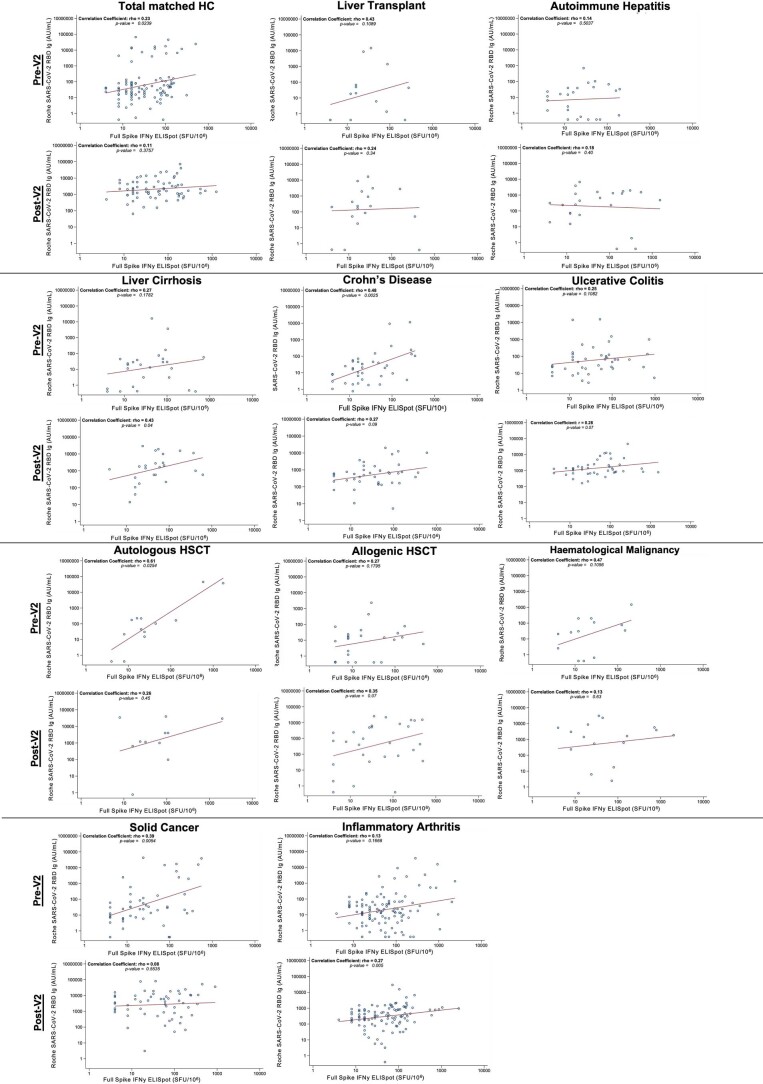
Correlation of anti-RBD Ig titre against full- Spike IFNy ELISpot magnitude at the pre- and post-V2 timepoints in OCTAVE subgroups. anti-SARS-CoV-2 RBD Ig titre (Roche assay) plotted against full- Spike IFNy ELISpot magnitude at the pre- and post-V2 timepoints in OCTAVE subgroups. Solid cancer, Crohn’s disease and Autologous-HSCT show positive correlations at pre-v2 but not post-v2. Inflammatory arthritis and liver cirrhosis sub-groups showed no significant correlation pre-V2 but showed weak positive correlation post-V2. Pearson correlations taken at each timepoint and individual sub-group.

T cell responses to spike and nucleocapsid were evaluated before V1, before V2 and 28 d after V2 using the Oxford Immunotec T-SPOT Discovery IFNγ ELISpot assay in 656 patients and 210 matched healthy controls. After V2, the hemodialysis (*P* < 0.003) and allo-HSCT (*P* < 0.003) groups had a significantly higher proportion of T cell non-response compared to the healthy control group (Fig. 3a and Supplementary Table 8). IFNγ-secreting T cell magnitude to spike antigens was lower in liver transplant (*z* = 3.821, *P* = 0.004) and allo-HSCT (*z* = 3.339, *P* = 0.03) groups compared to the healthy control group (Fig. 3b and Supplementary Table 9). To complement the serological characterization of Omicron BA.1 responses, an in-house IFNγ ELISpot assay was used to investigate T cell responses to ancestral and Omicron BA.1 spike in the 59-patient subset after V2. Regardless of prior SARS-CoV-2 status, the T cell response to full spike was maintained against Omicron peptides relative to ancestral (Fig. 3c,d), although stimulation with a reduced peptide set containing only peptides with variant amino acids relative to ancestral showed a significant decrease in Omicron reactivity (Fig. 3c). Serological and T cell responses showed weak correlations at pre-V2 only in healthy controls (*r* = 0.24, *P* = 0.02) but at pre-V2 and post-V2 timepoints in the overall patient cohort (pre-V1: *r* = 0.34, *P* < 0.0001; post-V2: *r* = 0.22, *P* < 0.0001) (Fig. 3e,f and Extended Data Fig. 6). In the ANCA-associated vasculitis group (with all patients taking B-cell-depleting therapies), there was no relationship between anti-RBD Ig and SARS-CoV-2 spike T cell responses; here, antibody responses were low after both vaccines, but many generated robust T cell responses after one vaccine (Fig. 3g,h). Positive correlations were also seen in disease subgroups at pre-V2 and/or post-V2 timepoints (Extended Data Fig. 6).

### Predictors of vaccine humoral and cellular responses

**Fig. 4:**
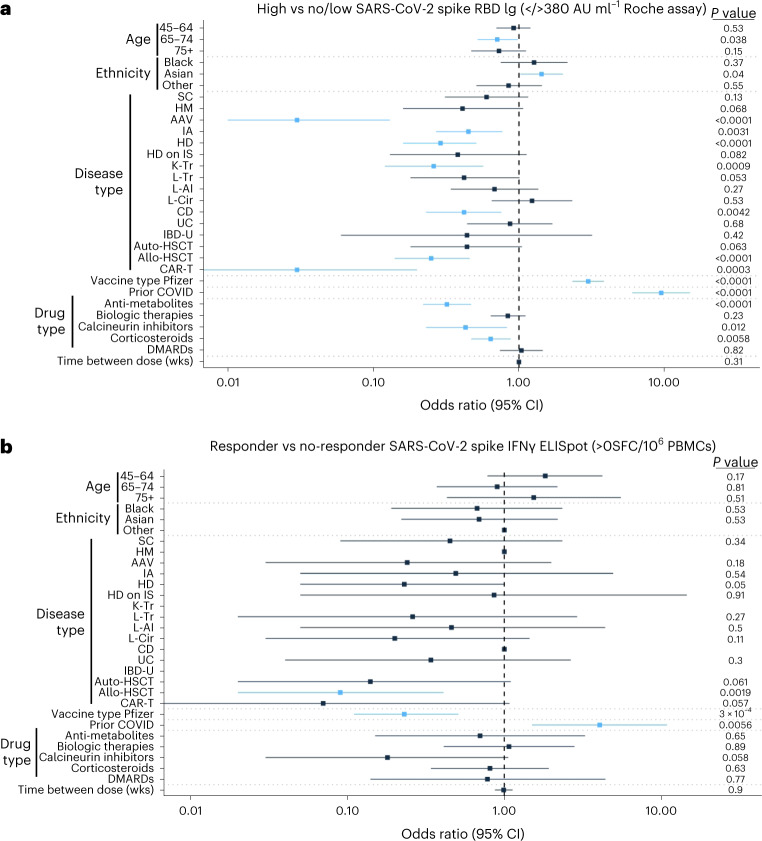
Predictors of anti-SARS-CoV-2 spike antibody and T cell responses after two doses of COVID-19 vaccine. Forest plot of multivariable logistic regression model fitted on post-V2 anti-SARS-CoV-2 RBD-binding total Ig antibody (whole group 1 and group 2 (*n* = 2,204) and matched healthy controls (*n* = 225)) (**a**) and IFNγ T cell responses (group 1 (*n* = 645) and matched healthy controls (*n* = 189)) (**b**). OR of anti-SARS-CoV-2 RBD-binding total Ig >380 AU ml^−1^ (**a**) and OR of anti-SARS-CoV-2 spike IFNγ T cell response ≥4 SFCs per 10^6^ PBMCs (**b**). Center of error bars represents OR, and whiskers represent 95% CI. *P* values are outputs of multivariable logistic regression model. *P* < 0.05 is significant and marked with blue lines. AAV, ANCA-associated vasculitis; CD, Crohn’s disease; DMARDs, disease-modifying anti-rheumatic drugs; HD, hemodialysis; HD on IS, hemodialysis on immunosuppression; HM, hematological malignancy; IA, inflammatory arthritis; L-AI, autoimmune hepatitis; L-Cir, liver cirrhosis; L-Tr, liver transplant; OSFC, zero spot forming cells; SC, solid cancer; UC, ulcerative colitis; wks, weeks.

**Extended Data Fig. 7:**
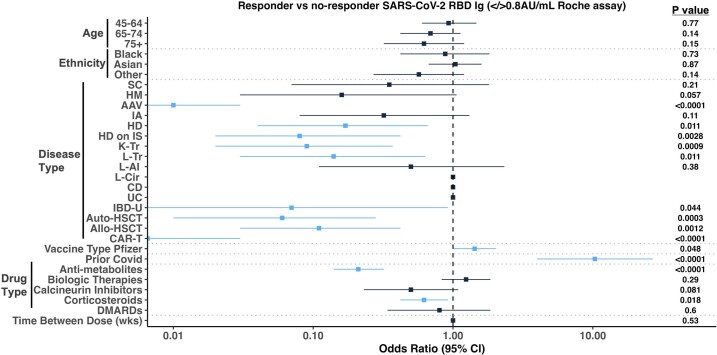
Multivariable logistic regression model presenting odds ratio of anti-SARS-CoV-2 RBD non- responsiveness after two COVID-19 vaccine doses. Forest plot depicting the outcome of a multivariable logistic regression model, presenting the odds ratio of non-response as measured by a Roche anti-RBD Ig titre of <0.8 AU/mL. All individuals in group 1 and group 2 with post-V2 serological titre included, except L-Cirr, UC, and CD groups which had no non-responders and therefore were not included in analysis. N = 1821 in disease group and n = 225 matched healthy controls.

The contribution of demographics, disease group, vaccine type, prior SARS-CoV-2, therapeutic regimen and time between vaccines to vaccine immunogenicity was assessed using multivariable logistic regression in OCTAVE patients compared to matched healthy controls. Patients aged 65–74 years had significantly lower odds of having a robust serological response (Roche anti-RBD Ig >380 AU ml^−1^) compared to patients in the 15–44-year age group (Fig. 4a and Supplementary Table 10). Patients of Asian versus White ethnicity had significantly higher odds of having a robust serological response (odds ratio (OR): 1.43, 95% confidence interval (CI) 1.02–2.01). Disease groups more likely to have a low or absent serological response (compared to the healthy control group) included ANCA-associated vasculitis (OR: 0.03, 95% CI 0.01–0.13), inflammatory arthritis (OR: 0.45, 95% CI 0.27–0.77), hemodialysis (OR: 0.29, 95% CI 0.16–0.51), kidney transplant (OR: 0.26, 95% CI 0.12–0.57), Crohn’s disease (OR: 0.42, 95% CI 0.23–0.76), allo-HSCT (OR: 0.25, 95% CI 0.14–0.46) and CAR-T (OR: 0.03, 95% CI 0–0.2). Patients receiving anti-metabolites (OR: 0.32, 95% CI 0.22–0.47), calcineurin inhibitors (OR: 0.43, 95% CI 0.23–0.83) and corticosteroids (OR: 0.64, 95% CI 0.47–0.88) were each more likely to have a low or absent serological response compared to healthy controls. Prior SARS-CoV-2 infection (OR: 9.48, 95% CI 6–14.97) and vaccination with BNT162b2 vaccine (OR: 2.99, 95% CI 2.33–3.84) significantly increased the odds of having a high serological response. These findings were generally recapitulated when analyzing the OR of likelihood of anti-RBD Ig seropositivity (>0.8 AU ml^−1^) (Extended Data Fig. 7 and Supplementary Table 11), although liver transplant (OR: 0.14, 95% CI 0.03–0.63) and hemodialysis on immunosuppression (OR: 0.08, 95% CI 0.02–0.42) disease groups were additionally associated with a decreased rate of seropositivity compared to the healthy control group.

In evaluating T cell responses, we used a responder threshold of ≥4 spot-forming cells (SFCs) per 10^6^ peripheral blood mononuclear cell (PBMCs) (Fig. 4b and Supplementary Table 12). The only disease group with reduced cellular responses was allo-HSCT (OR: 0.09, 95% CI 0.02–0.41). In contrast to the serological results, vaccination with BNT162b2 was associated with significantly decreased odds of generating a cellular response (OR: 0.23, 95% CI 0.11–0.51). Previous SARS-CoV-2 infection significantly increased the odds of generating a cellular response (OR: 4.05, 95% CI 1.5–10.9). No other variables were associated with T cell response.

### SARS-CoV-2 infection and COVID-19 severity

**Fig. 5:**
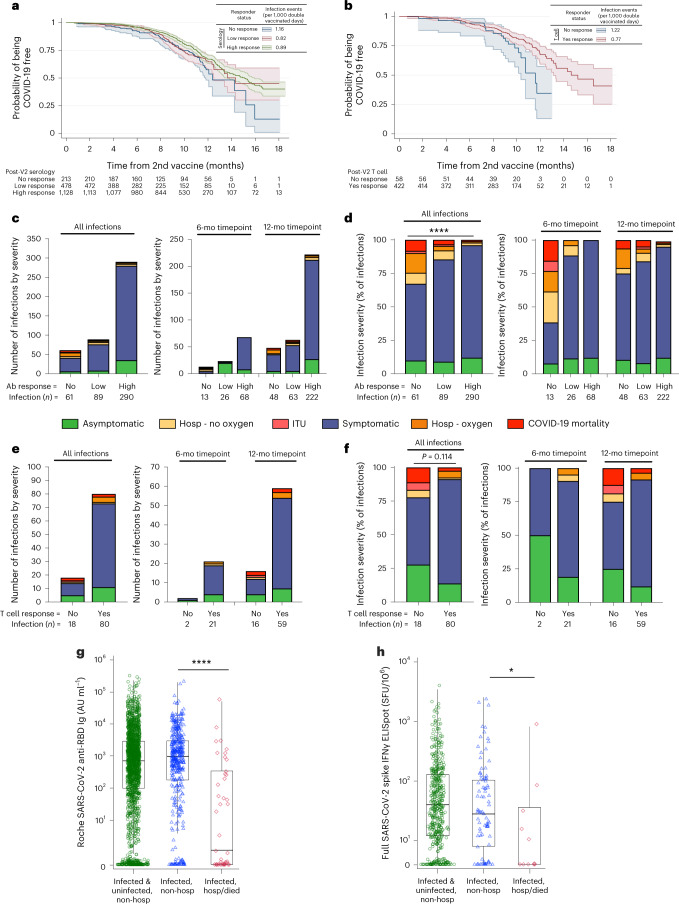
SARS-CoV-2 infection and severity after COVID-19 vaccination. **a**,**b**, COVID-19 incidence-free progression over time after second vaccine and infection rate per 1,000 double-vaccinated days, split by post-V2 anti-SARS-CoV-2 RBD-binding total Ig responder status (*n* = 1,617) (**a**) and post-V2 IFNγ T cell response to SARS-CoV-2 spike (*n* = 359) (**b**). Total number (**c**,**e**) and proportion (**d**,**f**) of SARS-CoV-2 infections stratified by COVID-19 disease severity and SARS-CoV-2 RBD-binding total Ig (*n* = 440)(**c**,**d**) or SARS-CoV-2 spike IFNγ T cell (*n* = 98) (**e**,**f**) response status. Severity data are shown at 6-month post-V2 and 12-month post-V1 timepoints and both timepoints combined, including only patients with known infection severity. **g**,**h**, Magnitude of post-V2 SARS-CoV-2 RBD-binding total Ig (*n* = 2,191) (**g**) and SARS-CoV-2 spike IFNγ T cell response (*n* = 573) (**h**) stratified by COVID-19 infection/severity—includes all non-hospitalized individuals with or without SARS-CoV-2 infection (infected and uninfected, non-hospitalized) and infected individuals who were not hospitalized with COVID-19 (infected, non-hospitalized) and individuals who were hospitalized or died with COVID-19 (infected, hospitalized). Fisher’s exact tests (**d**,**f**) or two-sided Mann–Whitney rank-sum statistical tests (**g**,**h**) were used without correction for multiple comparisons. **a**,**b**, Lines represent COVID-19-free progression, and shading represents 95% CI. **g**,**h**, Boxes represent median and IQR; whiskers represent ±1.5× IQR. *P < 0.05, ****P < 0.0001. Ab, anti-SARS-CoV-2 RBD-binding total Ig; Hosp–Oxygen, hospitalized with COVID-19 and required oxygen; Hosp–No Oxygen, hospitalized with COVID-19 but did not require oxygen; mo, month.

SARS-CoV-2 infection outcomes were collected in patients with both serology and infection data (V2 to 6 months after V2 in 1,648 patients and 6 months after V2 to 12 months after V1 in 1,617 patients). Overall, 474 infections were reported (Supplementary Table 13), including one infection that occurred during the Alpha VOC time epoch (14 January 2021–24 May 2021), 110 Delta (24 May 2021–20 December 2021), 336 Omicron (20 December 2021–17 October 2022) and 27 with exact infection date unknown. In total, 113 of 474 (24%) infections occurred within 6 months of V2, and 361 of 474 (76%) infections occurred at the >6-month timepoint. Most infections occurred in patients with kidney transplant, inflammatory arthritis and Crohn’s disease, with infection rates of 123/456 (27.0%), 79/689 (11.5%) and 67/156 (42.9%), respectively. Four hundred thirty-one infections were in patients who were previously infection naive (Supplementary Table 14), and 43 patients were previously SARS-CoV-2 infected. There was a higher rate of infection (infections per 1,000 d after V2) in patients with absent serological or T cell responses compared to those with high responses (Fig. 5a,b). However, most patients in OCTAVE overall had high serology (61.2%) and measurable T cell responses (88.5%), and most infections occurred in these groups (Fig. 5c–e and Supplementary Table 13).

Infection severity was evaluated in 440 of 474 (92.8%) infections. Most infections of known severity were mild (397/440, 90.2%), including asymptomatic infection (49/440, 11.1%) and symptomatic infection that did not require hospitalization (348/440, 79.1%) (Fig. 5c,d and Extended Data Table 1). Severe disease requiring hospitalization or COVID-19-related death was reported in 43 of 440 (9.8%) infections; 15 of 440 (3.4%) patients required oxygen; three patients were admitted to the intensive treatment unit (ITU) (0.7%); and 10 of 440 patients died (2.3%). Five patients died of COVID-19 without serological titers taken and were excluded from subsequent analysis. Infections occurring within 6 months after V2 (11/107 (10.2%)) were not more severe (hospitalized or died) than those at more than 6 months after V2 (32/333 (9.6%)) (Fig. 5c,d). Of 434 patients with known severity and precise date of infection, more severe infections occurred in those infected in the Delta versus the Omicron time epochs (eight died and 23 severe/107 Delta versus two died and 17 severe/327 Omicron; *P* < 0.0001). Severe disease occurred predominantly in patients with renal disease (hemodialysis 5/43 (11.6%), hemodialysis on immunosuppression 2/9 (22%) and kidney transplant 23/118 (19.5%)) (Extended Data Table 1). In some disease groups, infection rates were low but the proportion of severe disease was notably high (AAV (1/3, 33%), auto-HSCT (1/2, 50%) and CAR-T (3/3, 100%)). Low rates of severe disease were reported in ulcerative colitis (0/42, 0%) and Crohn’s disease (1/62, 1.6%). COVID-19-related deaths occurred in ANCA-associated vasculitis, hemodialysis, hemodialysis on immunosuppression, kidney transplant, auto-HSCT and CAR-T groups.

Infection severity was increased in patients with no (20/61, 32.3% severe) or low (13/89, 14.6%) post-V2 serological response compared to those with high serological response (10/290, 3.4%) (no versus low and high, *P* < 0.0001) (Fig. 5c,d), but post-V2 T cell responder status was not significantly associated with increased COVID-19 severity (4/18 (22.2%) non-response versus 7/80 (8.8%) response (*P* = 0.11, Fisher’s exact test)) (Fig. 5e,f and Extended Data Table 2). Of the COVID-19-related deaths, eight of 10 individuals had no detectable or low post-V2 serological response, and two of four (50%) individuals had no detectable T cell response. The magnitude of post-V2 anti-SARS-CoV-2 RBD Ig and spike-specific T cells were each significantly reduced (Ig: *P* < 0.0001, T cell: *P* = 0.033) in patients with severe COVID-19 compared to mild disease (Fig. 5g,h). These findings were generally recapitulated when patients infected at baseline were removed from analysis (Supplementary Tables 14–16).

### Adverse events

Adverse events (AEs) reported in 2,662 post-V1 and 2,629 post-V2 (Supplementary Tables 17 and 18) patients were generally mild (>97% of AEs after V1 and V2 were grade 1 or 2, with none higher than grade 3). Local injection site reactions were most common (27% post-V1 and 21% post-V2). Other common AEs included headache (16% post-V1 and 10% post-V2), chills (11% post-V1 and 4% post-V2), myalgia (10% post-V1 and 5% post-V2) and pyrexia/fever (10% post-V1 and 5% post-V2). Two serious adverse reactions (myalgia and cough) resulted in hospitalization but resolved without sequelae (Supplementary Table 19). One suspected unexpected serious adverse reaction (SUSAR) of thrombocytopenia was reported (Supplementary Table 20).

## Discussion

People with immune-suppressive disease remain vulnerable to COVID-19 (refs. ^27,29–31^), and identifying patient populations most at risk remains a UK government imperative. We show that, after two vaccines, in comparison to healthy volunteers, a substantial minority of immune-suppressed patients generated low-magnitude SARS-CoV-2 antibodies (in particular, ANCA-associated vasculitis on rituximab, hemodialysis on immunosuppressive therapy and solid organ transplant recipients), and that, although T cell responses were generally maintained, these were also reduced in some patient groups (hemodialysis, allo-HSCT and liver transplant recipients). Lower serological or T cell responses were associated with hospitalization or death from COVID-19.

Although vaccine correlates of immune protection against SARS-CoV-2 are not precisely defined, there is consensus that higher antibody titers are advantageous^33–38^. Waning immunity enhances disease susceptibility, especially in patients with additional comorbidities^39^, whereas higher levels of antibodies generated by booster vaccines are protective^40^. SARS-CoV-2-specific T cells protect against SARS-CoV-2 infection^41^ and appear less susceptible to viral escape as VOCs have emerged^42^. Previous studies highlighted the role of serological responses in protecting immune-suppressed patients, including (1) inflammatory arthritis with breakthrough infections increased in those who fail to seroconvert after vaccination^43^; (2) primary immune deficiency with increased COVID-19 mortality compared to the general population after vaccination^44^; and (3) renal disease with both breakthrough infection and COVID-19 severity/mortality correlating with serological responses^44–46^.

SARS-CoV-2 infection rates varied among disease subtypes and were higher in patients with no detectable antibody or T cells. However, infection rates cannot be confidently ascribed to disease phenotype, as social shielding behavior and SARS-CoV-2 exposure are likely to have differed among groups. However, disease severity in those infected can be definitively correlated with vaccine responsiveness. Although most (93.6%) patients had asymptomatic or mild infection, a substantial number had severe COVID-19 (33/440), and, additionally, 15 patients died. Patients with severe COVID-19 included ANCA-associated vasculitis, inflammatory arthritis, hemodialysis, hemodialysis on immunosuppression, kidney transplant, liver transplant, cirrhosis, Crohn’s disease, allo/auto-HSCT and CAR-T. Failure to seroconvert and the magnitude of the serological and cellular response were each associated with severe disease. However, one quarter of patients with severe disease seroconverted and had antibody levels similar to healthy controls, highlighting the fact that other factors contribute to disease susceptibility—for example, disease phenotype and/or comorbidities. Although most infections occcured during the Omicron time epoch, there were proportionally many more severe infections in the Delta epoch. The Omicron epoch coincided with the rollout of new therapeutic strategies, additional vaccines and a dominant VOC that is less pathogenic^32^, and it is not possible to disentangle the relative contribution of each of these to clinical outcomes in our study.

Vaccination with BNT162b2 generated higher antibody responses, whereas cellular responses were higher in patients who received ChAdOx1 nCoV-19, as previously shown in healthy populations^47–52^. Two studies in hemodialysis and solid organ transplant recipients assessed cellular responses in different vaccine types and showed no difference, but responses were low magnitude and the studies were underpowered to detect a difference^16,53^. The reasons why ChAd vaccines generate higher T cell responses than mRNA vaccines may relate to more persistent antigen expression in lymph nodes and the stimulation of distinct immune pathways, with ChAdOx dependent on robust follicular helper T (Tfh) cell responses^54^, whereas mRNA vaccines are Tfh independent^55^. Heterologous vaccination most effectively boosts T cell and antibody titers in healthy people and in solid organ transplant recipients^50,56,57^ and should be further evaluated in immune-suppressed patient groups. In this study, all immune-suppressant drug classes evaluated (other than biological therapies) were associated with a suboptimal serological response to vaccination, and, therefore, all patients receiving these therapies should be considered at risk for severe COVID-19.

In evaluating the effect of CCC cross-reactive antibodies on vaccines responsiveness, only HCoV-OC43 antibody titers (the CCC with the highest homology to SARS-CoV-2 spike^58–60^) were predictive of serological response to the first vaccine only. This shows that cross-reactive memory immune responses do not contribute to vaccine responsiveness once a significant SARS-CoV-2 memory pool has been established in immune-suppressed patients. Although T cell cross-reactivity using whole spike antigens against Omicron was maintained, antibody responses against all VOCs were decreased in both blood and saliva, and, at Roche anti-RBD Ig titers below 4,000 AU ml^−1^, most patients failed to neutralize Omicron BA.1. We suggest that this antibody titer threshold be explored in subsequent studies as a possible correlate of protection in immune-suppressed patients. New COVID-19 vaccine boosting regimens that account for the loss of recognition against emerging SARS-CoV-2 variants are likely to be particularly required for clinically vulnerable patients.

The strengths of our study include recruitment of a large number of patients and healthy controls, a wide range of disease phenotypes, national geographical spread, robust standardized procedures and standardization of timepoints with infection outcomes. Limitations include missing baseline data (due to the very rapid delivery of the vaccination program in vulnerable groups), no randomization for vaccine type, controls recruited in a separate study and heterogeneity of disease duration, severity, therapeutic regimen and comorbidities within groups. Additionally, OCTAVE assessed responses after two COVID-19 vaccines, whereas vulnerable patients have now typically received three or more vaccines. Nevertheless, because all healthy volunteers generate high-magnitude immune responses after two vaccines, the head-to-head comparison to assess relative vaccine effectiveness in disease cohorts compared to healthy controls remains informative.

Overall, our data are reassuring because most patients generated robust T cell responses and moderate serological responses and had mild COVID-19. However, the fact that some patients groups fail to generate high-magnitude immune responses, and the association of these with severe COVID-19, highlights the importance of developing strategies to (1) maximize cellular and humoral immune responses with new vaccine strategies; (2) protect patients by alternative therapeutic strategies^61–64^; and (3) continue to identify predictors of suboptimal vaccine responsiveness in immune-suppressed patients. Further studies on the effects of third and fourth vaccine doses alongside clinical outcomes are required. A national study (OCTAVE DUO) assessing third vaccine doses in immune-suppressed patients has fully recruited (ISRCTN15354495). Although our data may be directly used to inform COVID-19-related vaccination strategies in vulnerable patients, the future use of stored biological samples to evaluate biological pathways in secondary immune deficiency, alongside the relative risks of COVID-19 disease severity and immune responsiveness in disease subgroups, may inform clinical strategies in relation to vaccines or infection susceptibility in general.

## Methods

### Trial design and oversight

The OCTAVE trial is a multi-center, multi-disease, prospective cohort trial of the immune response to SARS-CoV-2 vaccination in patients receiving COVID-19 vaccination as part of routine, publicly funded National Health Service (NHS) care. The trial is a collaboration between the universities of Birmingham, Glasgow, Imperial College London, Oxford and Sheffield and is coordinated by the Cancer Research UK Clinical Trials Unit (CRCTU) at the University of Birmingham, which is the sponsor. The trial is conducted in accordance with Good Clinical Practice (GCP) guidelines. It was approved by the UK Medicines and Healthcare Products Regulatory Agency (MHRA) on 5 February 2021 and by the London and Chelsea Research Ethics Committee (REC ref.: 21/HRA/0489) on 12 February 2021. The protocol has subsequently been amended eight times with five substantial amendments (with ethical approvals dated 3 March 2021, 19 April 2021, 24 December 2021 and 4 April 2022) and three non-substantial amendments: protocol versions dated 22 April 2021, 14 July 2021 and 10 September 2021. The trial is registered on ISRCTN12821688.

### Patients recruited into OCTAVE

Written informed consent was obtained from all participants. The trial is recruiting adult and pediatric patients. Adult patients in clinically vulnerable groups were recruited between 19 February 2021 and 1 October 2021 (last vaccine administered) based on the following eligibility criteria:

- Are eligible for vaccination by one of the SARS-CoV-2 vaccines approved by the MHRA, administered in accordance with national guidelines.
- Have not received their second dose of the vaccine for the ‘deep immunophenotyping group’ or have not passed the day 28 post-second vaccine dose timepoint (21–84 d after second vaccination) for the ‘serology group’.
- Have an anticipated lifespan of 6 months or longer.
- Have a diagnosis belonging to one of the following disease groups: solid cancer; hematological malignancy; rheumatic inflammatory conditions (including ANCA-associated vasculitis on rituximab and inflammatory arthritis), chronic renal disease (including end-stage kidney disease (patients on hemodialysis and hemodialysis with immunosuppression) and kidney transplantation), chronic liver disease (including liver cirrhosis, liver disease on immunosuppressive therapy and liver transplantation), inflammatory bowel disease on immunosuppressive therapy (Crohn’s disease, ulcerative colitis and undefined inflammatory bowel disease), HSCT patients and CAR-T therapies. CAR-T patients are those who most recently received CAR-T as treatment in their therapeutic course. Inclusion criteria are included in the trial protocol (Supplementary Appendix 1).

All patients who fulfilled the patient characteristics inclusion criteria could be enrolled into either study group. Investigators generally recruited patients into group 1 first where possible, before patients had received two vaccines as part of the rapid UK COVID-19 vaccine program. Some patients opted for group 2 because fewer study visits were involved (patient choice).

OCTAVE recruited 2,686 adult patients: 2,012 patients into the serology group and 674 patients into the deep immune phenotyping group (see Supplementary Table 21 for recruitment per site). The full protocol (Supplementary Appendix 1) is available in the supplementary material.

The deep immunophenotyping cohort was assessed pre-vaccine (baseline), pre-second vaccine dose (pre-V2), 28 d post-second vaccine dose (post-V2, within ±3 d), 6 months post-second vaccine dose and 12 months after the first vaccine dose (as close to timepoints as possible). The serology cohort was assessed 28 d post-second vaccine dose (−7/+56 d) and followed up 6 months post-second vaccine dose and 12 months after the first vaccine dose (as close to timepoints as possible).

Anti-RBD total Ig immunogenicity data were available in 2,204 patients and 225 healthy controls. T cell data were available in 656 patients in the deep immunophenotyping cohort and in 210 controls. Infection data were available in 1,648 OCTAVE patients at the <6-month post-V2 timepoint and 1,613 OCTAVE patients at the 6-month post-V2 to 12-month post-V1 timepoint. Severity data were available in 93% of those infected. AEs were reported in 2,662 patients post-V1 and 2,669 OCTAVE patients post-V2. Detailed information regarding the number of samples included in each immunogenicity assays is available in Extended Data Fig. 1.

After trial entry, 66 adult participants were found to have been recruited at the wrong timepoint in accordance with the eligibility criteria: ‘Have not received their second dose of the vaccine for the ‘deep immunophenotyping group’ or have not passed the day 28 post-second vaccine dose timepoint (21–84 d after second vaccination) for the ‘serology group’’; and, for 24 participants, trial consent was obtained after the collection of post-booster samples (although these patients were recruited into another ethically approved study with full consent) before trial consent in OCTAVE. This was reported to the MHRA as a serious breach, and patients were still included in the analysis.

### Outcome measures

The primary outcomes for this trial are the magnitude of the anti-SARS-CoV-2 IgG antibodies and the magnitude of the T cell responses to SARS-CoV-2 peptides after vaccination. The secondary outcome is the proportion of first-symptomatic, PCR-proven COVID-19 infection 14 d after V1 in participants without evidence of prior COVID-19 infection. The exploratory outcomes are described in detail in the protocol (Supplementary Appendix 1).

This manuscript represents the definitive analysis of the primary outcome for the adult cohort.

### Vaccine administration

Vaccine (BNT162b2 (Pfizer/BioNTech) or ChAdOx1 nCoV-19 vaccine) was administered in line with its temporary authorization under Regulation 174 of the Human Medicines Regulations 2012, the national recommendations and guidance of the Joint Committee on Vaccination and Immunisation (JCVI) and current standard NHS practice. The trial has no influence on the type of vaccine given or the timing of the booster vaccine delivery. Vaccines were administered both through NHS pathways and by OCTAVE study investigators. The interval between vaccines was in accordance with national recommendations and the guidance of the JCVI. As vaccines were being given to new patient populations, this study was registered with the MHRA (UK MHRA clinical trial authorization number: 21761/0365/001).

### AEs

AEs were captured up to 28 d after the second vaccine and were graded 1–5 using the Common Terminology Criteria for Adverse Events (CTCAE), version 4.03.

### Sample collection

Serum samples were collected 4 weeks post-second dose (−7/+14 d) for all participants, alongside whole blood for the Oxford Immunotec assay, PBMCs and plasma, when feasible. Where available, baseline (pre-vaccine samples, including samples that may have been collected before recruitment to OCTAVE) or pre-second dose samples taken any time after V1 but before the second dose were included. All samples were collected in accordance with national regulations and requirements, including standard operating procedures for logistics and infrastructure. Samples were taken in appropriately licensed premises and stored and transported in accordance with Human Tissue Authority guidelines and NHS Trust policies.

### Anti-SARS-CoV-2 Ig analysis

The magnitude of anti-SARS-CoV-2 antibodies was measured using the Roche Elecsys AntiSARS-CoV-2 S and Roche Elecsys AntiSARS-CoV-2 N assays by the UKHSA Laboratories at Porton Down. The Roche assay measures the presence and the amount of serum antibodies to the spike RBD antigen of SARS-CoV-2. Seroconversion is defined as a response equal to or greater than 0.8 AU ml^−1^, and no response is defined as less than 0.8 AU ml^−1^. Low response was defined on the Roche anti-RBD Ig assay after evaluation of the serological response to vaccine in healthy controls. A cutoff for low was defined as the upper value of the bottom decile of healthy controls.

### T-SPOT DISCOVERY SARS-CoV-2 assay

**Extended Data Fig. 8:**
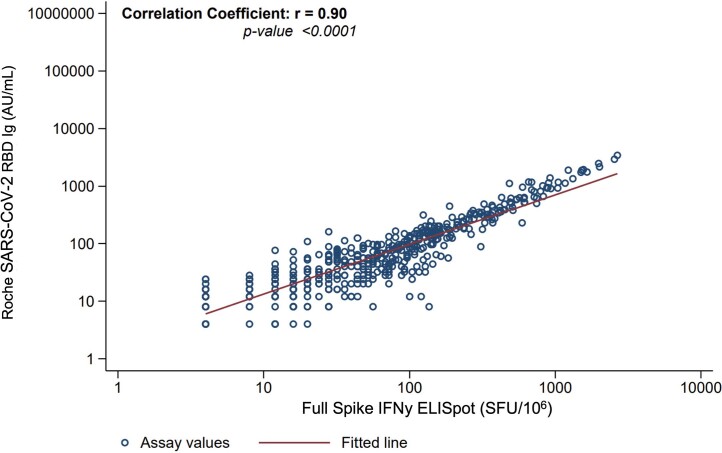
Correlation of Oxford Immunotec T-Spot SARS-CoV-2 spike peptide pools. Exploratory scatter plot to show correlation between the ELISpot Spike 1 + 2 & Full Spike Assay measures, for all patients with both values available in the Deep Immunophenotyping Group. Presented on the log scale. Pearson’s correlation.

The magnitude of the T cell responses was measured using the T-SPOT DISCOVERY SARS-CoV-2 assay by Oxford Immunotec (https://www.tspotdiscovery.com/). Peptide pools representing the full spike (S) proteins, subunits S1 and S2, nucleocapsid and membrane, plus positive (phytohaemagglutinin) and negative controls were used to stimulate 250,000 PBMCs separated from fresh whole blood. IFNγ-secreting T cells were enumerated on an automated plate reader. Final values were calculated by subtracting the negative control and multiplying by 4 to define the number of IFNγ-secreting T cells per 10^6^ PBMCs. Values greater than or equal to four IFNγ-secreting T cells per 10^6^ PBMCs were defined as a positive response. In the renal cohort (hemodialysis with immunosuppression and hemodialysis), the full spike peptide pool was not included in the assay at all timepoints. To generate equivalent data, the S1 and S2 values were combined, and a cutoff of four IFNγ-secreting T cells per 10^6^ PBMCs was used for positivity. There was strong correlation between S1 + S2 and full spike pools in this assay (*r* = 0.90, *P* < 0.0001) (Extended Data Fig. 8).

### IFNγ T cell ELISpot assay

Frozen PBMCs were thawed, and the Human IFNγ ELISpot Basic Kit (Mabtech, 3420-2A) at Oxford University laboratories was used, as in ref. ^65^, but using 200,000 cells per well in duplicate. For antigens, we used overlapping peptide pools (18-mers with 10 amino acid overlap, mimotopes) representing ancestral spike (S1 and S2), Omicron (B.1.1.529 and BA.1) spike (S1 and S2), ancestral membrane and nucleocapsid SARS-CoV-2 proteins and pools of ancestral or Omicron 18-mer peptides covering the mutated regions of Omicron at a final concentration of 2 μg ml^−1^. The mean spots of the negative control wells were subtracted from the test wells and then multiplied by 5 to give antigen-specific responses expressed as SFU per 10^6^ PBMCs. Total spike responses were defined by adding S1 and S2 responses together.

### Meso Scale Discovery IgG, IgA and IgM binding assays

IgG, IgA and IgM responses to SARS-CoV-2, SARS-CoV-1 and seasonal coronaviruses were measured using a multiplexed Meso Scale Discovery (MSD) immunoassay: V-PLEX COVID-19 Coronavirus Panel 2 Kit (K15369U-2) from Meso Scale Diagnostics. IgG was measured in all group 1 participants at all available timepoints; IgA and IgM were measured in ulcerative colitis, Crohn’s disease, liver transplant, autoimmune liver disease, cirrhosis, inflammatory arthritis and ANCA-associated vasculitis disease groups at all available timepoints. A MULTI-SPOT 96-well, 10-spot plate was coated with four SARS-CoV-2 antigens (spike, RBD, nucleoprotein and NTD) and SARS-CoV-1 spike trimer as well as spike proteins from seasonal human coronaviruses, OC43, HKU1, 229E and NL63 and BSA. Antigens were spotted at 200−400 μg ml^−1^ (MSD Coronavirus Plate 2). Multiplex MSD assays were performed as per the manufacturerʼs instructions. To measure IgG, IgA and IgM binding antibodies, 96-well plates were blocked with MSD Blocker A for 30 min. After washing with washing buffer, plasma samples diluted 1:1,000–10,000 in diluent buffer were added to wells, along with MSD standard or undiluted MSD internal controls. After a 2-h incubation and a washing step, detection antibody (MSD SULFO-TAG Anti-Human IgG, IgA or IgM Antibody, 1/200) was added. After washing, MSD GOLD Read Buffer B was added, and plates were read using a MESO SECTOR S 600 Reader. The standard curve was established by fitting the signals from the standard using a four-parameter logistic model. Concentrations of samples were determined from the electrochemiluminescence signals by backfitting to the standard curve and multiplied by the dilution factor. Concentrations are expressed in AU ml^−1^. Cutoffs were determined for each SARS-CoV-2 antigen (spike, RBD, nucleoprotein and NTD) based on 64 pre-SARS-CoV-2 pandemic sera (average concentration + 3× standard deviation for IgG, IgA and IgM binding) (Supplementary Table 22). As samples were from UK individuals with low probability to have been exposed to SARS-CoV-1, a cutoff for SARS-CoV-1 spike was similarly determined.

Alternatively, IgG responses to SARS-CoV-2 variant spike antigens, including Wuhan (ancestral) strain, Alpha, Beta, Gamma, Delta and Omicron (BA.1), were similarly measured using a multiplexed V-PLEX SARS-CoV-2 Panel 23 Kit (K15567U-2) from Meso Scale Diagnostics.

### MSD ACE2 inhibition surrogate neutralization assay

V-PLEX SARS-CoV-2 Panel 23 Kit was used to measure the ability of oral fluid or serum samples to inhibit ACE2 binding to different variants of SARS-CoV-2 spike, including B lineage Wuhan-Hu-1 spike, B.1.1.7/Alpha, B.1.351/Beta, P.1/Gamma, B.1.617.2/Delta and B.1.1.529;BA.1/Omicron. Assays were performed as per the manufacturer’s instructions with neat oral fluid samples or diluted sera. To measure ACE2 inhibition, 96-well MSD plates were blocked with MSD Blocker for 30 min. Plates were then washed in MSD washing buffer, and 25 μl of undiluted oral fluid samples or diluted sera (1/10–1/100) were added to the plate. After 1-h incubation, recombinant human ACE2-SULFO-TAG was added to all wells. After a further 1 h, plates were washed, and MSD GOLD Read Buffer B was added; plates were then immediately read using a MESO SECTOR S 600 Reader. Neutralizing activity was determined by measuring the presence of antibodies able to block the binding of ACE2 to SARS-CoV-2 spike proteins from Wuhan-Hu-1 spike, B.1.1.7/Alpha, B.1.351/Beta, P.1/Gamma, B.1.617.2/Delta and B.1.1.529;BA.1/Omicron and was expressed as percentage of ACE2 inhibition in comparison to the blanks on the same plate.

### Microneutralization assay

Sera were serially diluted in DMEM supplemented with 1% FBS from an initial dilution of 1:10 to 1:10,000. Equal volumes of diluted sera and SARS-CoV-2 virus (approximately 100 foci-forming units (FFU)) were combined and incubated for 30 min. Viruses used in this assay were Victoria (VIC01) and Omicron (B.1.1.529 (BA.1)). After incubation, 100 µl of Vero E6 cells (4.5 × 10^5^ per milliliter) was added to each well, and virus was allowed to infect the cells for 2 h at 37 °C and 5% CO_2_, followed by the addition of 100 µl of carboxymethyl cellulose (1.5%) to each well. The plates were incubated for a further 20–22 h at 37 °C and 5% CO_2_. All assays were carried out in triplicate.

Cells were washed with 200 µl of DPBS and then fixed with paraformaldehyde 4% v/v (100 µl per well) for 30 min at room temperature. Cells were permeabilized with Triton X-100 (2% in PBS) and then stained for SARS-CoV-2 nucleoprotein using a human monoclonal antibody (FB9B). Bound antibody was detected after incubation with a goat anti-human IgG HRP conjugate (Sigma-Aldrich) and, after TrueBlue Peroxidase Substrate (Insight Biotechnology) addition, imaged using an ELISpot reader. The IC_50_ was defined as the concentration of compound that reduced the FFU by 50% compared to the control wells.

### Clinical data

Clinical data were collected electronically from participating sites using a REDCap (Research Electronic Data Capture) database held at the CRCTU. Data collected on trial entry included sex, ethnicity, body mass index (BMI), smoking status, medical history and details of prior COVID-19 infection. Details of disease cohort treatment, COVID-19 vaccination, AEs and subsequent COVID-19 infection were collected during the course of the trial.

### SARS-CoV-2 infection and disease severity

SARS-CoV-2 infection was captured at each study visit in all patients through to V1 + 12 months between 14 January 2021 and 17 October 2022. Symptomatic infection (SARS-CoV-2 confirmed using PCR assay or lateral flow antigen test) was captured through (1) direct patient interview at all follow-up visits; (2) systematic review of the electronic patient record and/or regional databases; and (3) telephone interviews as required. Infection with a given VOC was defined as COVID-19 infection within time epochs where the VOC was most prevalent in the UK: Alpha, 14 January 2021 (study start) to 24 May 2021; Delta, 24 May to 20 December 2021; and Omicron 20 December 2021 onwards based on UK prevalence (https://covariants.org/). Disease severity was recorded on all patients with infection where available. If two infections were reported after V2, then only the first infection was included in subsequent analysis (*n* = 9).

### Control group

The healthy controls group was derived from participant data and samples from two sources: the UK PITCH cohort and the UKHSA CONSENSUS cohort^66^. PITCH is a prospective, multi-center study assessing T cell responses to COVID-19 natural infection and vaccination^59^. Healthcare worker participants received SARS-CoV-2 vaccination as part of workplace programs. PITCH is a sub-study of the SIREN study (trial ID: 252 ISRCTN11041050), which was approved by the Berkshire REC, Health Research 250 Authority (IRAS ID: 284460, REC ref.: 20/SC/0230), with PITCH recognized as a sub-study on 2 December 2020. Some participants were recruited under PITCH-aligned study protocols. In Oxford, participants were recruited under the GI Biobank Study 16/YH/0247, approved by the Yorkshire & The Humber–Sheffield REC on 29 July 2016, which was amended for this purpose on 8 June 2020. In Liverpool, some participants were recruited under the ‘Human immune responses to acute virus infections’ study (16/NW/0170), approved by the North West–Liverpool Central REC on 8 March 2016 and amended on 14 September 2020 and 4 May 2021. In Sheffield, participants were recruited under the Observational Biobanking study STHObs (18/YH/0441), which was amended for this study on 10 September 2020. This study was conducted in compliance with all relevant ethical regulations for work with human participants and according to the principles of the Declaration of Helsinki (2008) and GCP guidelines. Written informed consent was obtained from all participants enrolled in the study.

Data from 986 participants were available from the two cohorts. The healthy controls group was then sorted to ensure that all control data met the following criteria:

- A post-boost sample was available.
- Data relating to age, sex, prior COVID status and vaccination type were available.
- Samples were taken within the same timeframe defined for the OCTAVE participant samples.

After sorting, the healthy control group was matched to the OCTAVE patient group based on prior COVID status and vaccine type using a proportional matching method. The relevant participants for each group were then randomly selected from the available control data to match the four analysis groups:

- Complete OCTAVE dataset (deep immunophenotyping and serology groups together)
- OCTAVE deep immunophenotyping only
- OCTAVE serology group only
- OCTAVE renal disease group only

A separate analysis group was created for the renal disease group, as these patients did not have the full spike peptide pool tested on the T-SPOT DISCOVERY at all timepoints due to the timing of recruitment.

### Statistical analysis

#### Dataset preparation

A healthy control data pool comprising 986 participants (231 PITCH study and 755 UKHSA dataset) was sampled to create the healthy controls analysis group by matching, as closely as possible, the proportions of age, sex, prior COVID status and vaccination type, as observed in the OCTAVE recruits. ELISpot assay raw data required processing before analytical use; the control blank readings were subtracted from the sample readings, and any negative numbers were replaced with zero; the new values were then multiplied by 4 to give cell counts per million.

### Analysis

Descriptive statistics data are presented as number of observations with percentages and medians with interquartile ranges (IQRs), unless indicated otherwise. Data were questioned using two-sided statistical tests, including Kruskal–Wallis H test (as data non-normal) with post hoc Dunn’s testing for pairwise comparisons, χ^2^ and Fisherʼs exact tests where required. Where multiple testing occurred, *P* values were adjusted using Bonferroni corrections. Pearson’s correlations and logistic regressions investigated data relationships. COVID-19-free time post-second vaccination was estimated using the method of Kaplan and Meier (1958). Time was taken from date of the second vaccine to date last seen. COVID-19 events were taken as the date of confirmed SARS-CoV-2 infection or date of death confirmed as being COVID-19 related. The number of SARS-CoV-2 infection events per 1,000 d was calculated by using (total number of SARS-CoV-2 infections) / (total number of days at risk post-second vaccination) × 1,000 = number of SARS-CoV-2 infection events per 1,000 d. COVID-19 disease severity was defined as asymptomatic; symptomatic without hospitalization; hospitalization with COVID-19 without requirement of oxygenation; hospitalization with COVID-19 with requirement of oxygenation; admission to ITU with COVID-19; or death related to COVID-19. Data were visualised using various plot types: box and whisker with overlaid data points using the jitter function to aid interpretation; vertically stacked bar charts of percentages of response types (non-responder, low-responder and responder); scatter plots with Pearson’s correlation analysis line; connected scatter plot showing changes; and matrix (panel) correlation plots illustrating assay results. All analyses used the statistical package Stata version 17.0 (StataCorp), GraphPad Prism (version 9.4.0) or R (version 4.2.1) with RStudio 2022.02.3.

### Statistics and reproducibility

The OCTAVE trial is a multi-center, multi-disease, prospective observational cohort trial. Statistical analyses were completed, independently replicated and compared to initial results. No discrepancies were found. The sample size was based on an estimation of the number of participants who could be recruited within the short space of time required. An effect size calculation, based on a *t*-test, was done to provide information as to whether the number of recruits would be sufficient. Missing data were excluded from the analyses. No other data were omitted. As an observational study, experiments were not randomized, and investigators were not blinded to allocation.

### Reporting summary

Further information on research design is available in the Nature Portfolio Reporting Summary linked to this article.

## Online content

Any methods, additional references, Nature Portfolio reporting summaries, source data, extended data, supplementary information, acknowledgements, peer review information; details of author contributions and competing interests; and statements of data and code availability are available at 10.1038/s41591-023-02414-4.

### Supplementary information

Supplementary Information Supplementary Tables 1–22 and OCTAVE Study Protocol.

Reporting Summary

## Figures and Tables

**Extended Data Table 1:**
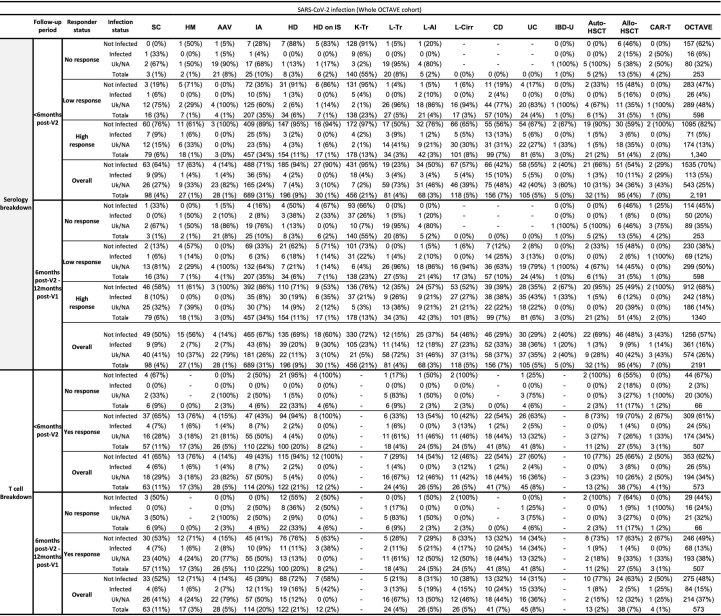
Summary of COVID-19 severity after COVID-19 vaccination split by serological response

Summary of COVID-19 severity reported in combined follow-up periods after COVID-19 vaccination for all immunocompromised patients in OCTAVE, split by disease subgroup and based on Roche anti-RBD Ig assay result ≥0.8 AU ml^−1^, low response <380 AU ml^−1^ and high response >380 AU mL^−1^ at the post-V2 timepoint.

^*#*^ % of total OCTAVE infections.

**Extended Data Table 2:**
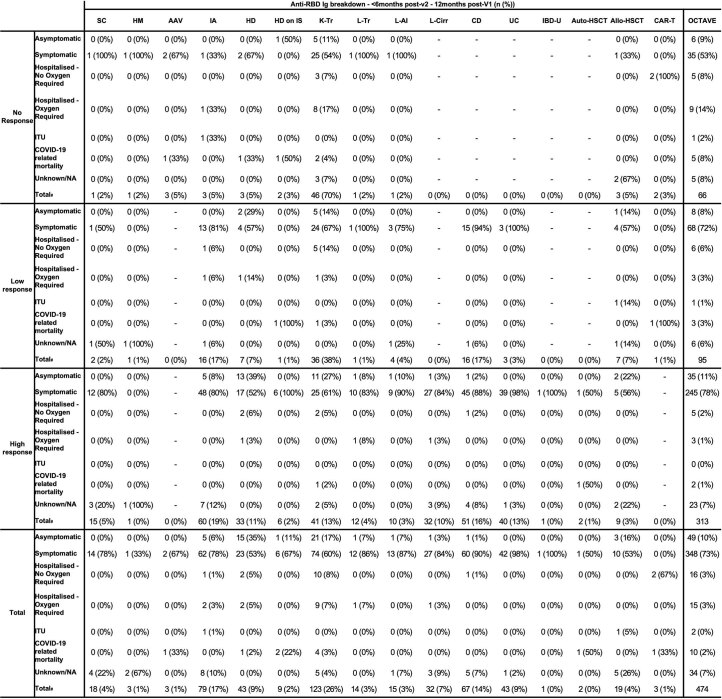
Summary of COVID-19 severity after COVID-19 vaccination split by T cell response

Summary of COVID-19 severity in reported SARS-CoV-2-infected individuals after vaccination in group1 of the OCTAVE cohort, split by disease subgroup and based on IFNγ ELISpot response to SARS-CoV-2 spike at the post-V2 timepoint. No response = 0SFC/10^6^; yes response > 0SFC/10^6^.

* Kidney transplant and undefined inflammatory bowel disease groups were not included in group 1.

^*#*^ % of total OCTAVE infections.

## Data Availability

Participant data and the associated supporting documentation will be available for requesting within 6 months after the publication of this manuscript. Details of our data request process are available on the CRCTU website. Only scientifically sound proposals from appropriately qualified research groups will be considered for data sharing. The decision to release data will be made by the CRCTU Director’s Committee, which will consider the scientific validity of the request, the qualifications and resources of the research group, the views of the Chief Investigator and the trial steering committee, consent arrangements, the practicality of anonymizing the requested data and contractual obligations. A data-sharing agreement will cover the terms and conditions of the release of trial data and will include publication requirements, authorship and acknowledgements and obligations for the responsible use of data. An anonymized encrypted dataset will be transferred directly using a secure method and in accordance with the University of Birmingham’s IT guidance on encryption of datasets. Information on data requests, including a contact address and expected timeframe of requests, can be found at https://www.birmingham.ac.uk/research/crctu/data-sharing-policy.aspx.

## Code availability

Code is available upon reasonable request through the same process as data, as detailed on the CRCTU website: https://www.birmingham.ac.uk/research/crctu/data-sharing-policy.aspx.
