# Supplementary material for: SARS-CoV-2-specific immune responses and clinical outcomes after COVID-19 vaccination in patients with immune-suppressive disease
Source: Nat Med. 2023 Jul 6;29(7):1760–74. doi: 10.1038/s41591-023-02414-4 (PMC10353927; doi:10.1038/s41591-023-02414-4)
Supplement: Supplementary file 1 — Supplementary Tables 1–22 and OCTAVE Study Protocol. [file 41591_2023_2414_MOESM1_ESM.pdf]

nature medicine

Article

https://doi.org/10.1038/s41591-023-02414-4

SARS-CoV-2-specific immune responses and clinical outcomes after COVID-19 vaccination in patients with immune-suppressive disease

In the format provided by the authors and unedited

Healthy Controls			Disease Sub-Group			
	**No Response, N(%)**	**Responder, N(%)**		**No Response, N(%)**	**Responder, N(%)**	**p-value**
Healthy Controls	3 (1.33%)	222 (98.67%)	OCTAVE	255 (11.57%)	1949 (88.43%)	<0.001*
			SC	3 (1.8%)	95 (96.84%)	0.373
			HM	2 (7.41%)	25 (92.59%)	0.090
			AAV	21 (72.41%)	8 (27.59%)	<0.001*
			IA	25 (3.62%)	665 (96.38%)	0.116
			HD	9 (4.55%)	189 (95.45%)	0.075
			HD on IS	6 (20.00%)	24 (80.00%)	<0.001*
			K-Tr	141 (30.79%)	317 (69.21%)	<0.001*
			L-Tr	20 (24.69%)	61 (75.31%)	<0.001*
			L-AI	5 (7.35%)	63 (92.65%)	0.018
			L-Cir	0 (0.00%)	120 (100.00%)	0.554
			CD	0 (0.00%)	156 (100.00%)	0.273
			UC	0 (0.00%)	107 (100.00%)	0.554
			IBD-U	1 (20.00%)	4 (80.00%)	0.085
			Auto-HSCT	5 (15.15%)	28 (84.85%)	0.001*
			Allo-HSCT	13 (13.54%)	83 (86.46%)	<0.001*
			CAR-T	4 (50.00%)	4 (50.00%)	<0.001*

Supplementary Table 1: Fisher’s Exact test results investigating the different response proportions between no (<0.8AU/mL) responses and any responses measured using the Roche anti-RBD Ig assay following the second Covid vaccination. Significant values after Bonferroni correction (alpha =0.003) are marked with an *.

Obs Number	Disease Group	Post-hoc Dunn's Test (z score)	p-value
98	SC	-1.156	1.00
27	HM	0.984	1.00
29	AAV	8.419	<0.001*
690	IA	4.915	<0.001*
198	HD	-2.347	0.99
30	HD on IS	2.441	0.77
458	K-Tr	10.580	<0.001*
81	L-Tr	6.822	<0.001*
68	L-AI	2.830	0.24
120	L-Cir	-0.665	1.00
156	CD	4.319	0.001*
107	UC	1.646	1.00
33	Auto-HSCT	1.154	1.00
96	Allo-HSCT	4.178	0.002*

Supplementary Table 2: Post-hoc Dunn's test (two-sided) to determine which sub-disease group(s) were different when compared to the healthy control group (n = 225) for Roche anti-RBD Ig assay response following the second vaccination (results include non-responders). Significant values after Bonferroni correction (alpha =0.003) are marked with an *.

Healthy Controls			Disease Sub-Group				
	**No or low Response, N(%)**	**High Response, N(%)**		**No or low Response, N(%)**	**High Response, N(%)**	**Test statistic**	**p-value**
Healthy Controls	37 (16.44%)	188 (83.56%)	OCTAVE	855 (38.79%)	1349 (61.21%)	χ^2=43.8819	<0.001*
			SC	19 (19.39%)	79 (80.61%)	χ^2=0.4127	0.521
			HM	9 (33.33%)	18 (66.67%)	χ^2=4.6081	0.032
			AAV	26 (89.66%)	3 (10.34%)	NA (Fisher's Exact)	<0.001*
			IA	233 (33.77%)	457 (66.23%)	χ^2=24.4799	<0.001*
			HD	43 (21.72%)	155 (78.28%)	χ^2=1.9093	0.167
			HD on IS	13 (43.33%)	17 (56.67%)	χ^2=12.1413	<0.001*
			K-Tr	279 (60.92%)	179 (39.08%)	χ^2=120.0329	<0.001*
			L-Tr	47 (58.02%)	34 (41.98%)	χ^2=51.7048	<0.001*
			L-AI	26 (38.24%)	42 (61.76%)	χ^2=14.6906	<0.001*
			L-Cir	17 (14.17%)	103 (85.83%)	χ^2=0.3076	0.579
			CD	57 (36.54%)	99 (63.46%)	χ^2=20.0150	<0.001*
			UC	24 (22.43%)	83 (77.57%)	χ^2=1.7322	0.188
			IBD-U	2 (40.00%)	3 (60.00%)	NA (Fisher's Exact)	0.200
			Auto-HSCT	11 (33.33%)	22 (66.67%)	χ^2=5.4207	0.020
			Allo-HSCT	44 (45.83%)	52 (54.17%)	χ^2=30.8055	<0.001*
			CAR-T	5 (62.50%)	3 (37.50%)	NA (Fisher's Exact)	0.006

Supplementary Table 3: Results of the Chi2 or Fisher's Exact tests (as appropriate) investigating the different response proportions between no (<0.8AU/mL) or low (<380AU/mL) responses and high responses measured using the Roche anti-RBD Ig assay following the second Covid vaccination. Significant values after Bonferroni correction (alpha =0.003) are marked with an *.

Disease Group	No Prior Covid (N)	Confirmed Prior Covid (N)	Test Statistic (z)	p-value
HC	196	29	-5.734	<0.0001*
OCTAVE	1901	303	-16.807	<0.0001*
SC	85	13	-2.676	0.0075
HM	25	2	-0.093	0.9573 #
AAV	27	2	-1.748	0.1330 #
IA	621	69	-6.940	<0.0001*
HD	99	99	-8.720	<0.0001*
HD on IS	20	10	-3.489	0.0002*
K-Tr	419	39	-6.462	<0.0001*
L-Tr	74	7	-2.411	0.0142 #
L-AI	65	3	-1.598	0.1186 #
L-Cir	106	14	-2.379	0.0174
CD	142	14	-3.413	0.0006*
UC	95	12	-3.189	0.0014*
IBD-U	0	5	NA‡	NA‡
Auto-HSCT	27	6	-3.223	0.0004*#
Allo-HSCT	86	10	-1.741	0.0816
CAR-T	5	3	-0.953	0.3406#

‡Analysis could not be done as the five IBD-U patients were all recorded as not having a prior Covid infection.

# Exact test p-values were used due to the small observation numbers.

Supplementary Table 4: Results table of Wilcoxon rank-sum test investigating differences in immune response following the second vaccination as quantified by the Roche anti-RBD Ig assay in patients with or without prior SARS-CoV-2 infection. Significant values after Bonferroni correction (alpha =0.003) are marked with an *.

Disease Group	ChAdOx1 nCoV-19 (N)	BNT162b2 (N)	Test Statistic (z)	p-value
HC	151	74	-6.593	<0.0001*
OCTAVE	1497	695	-9.959	<0.0001*
SC	39	58	-3.539	0.0004*
HM	18	8	-1.111	0.2664
AAV	29	0	NA	NA
IA	582	108	-10.280	<0.0001*
HD	116	82	-2.611	0.0090
HD on IS	24	6	-1.067	0.3004 #
K-Tr	183	271	-5.576	<0.0001*
L-Tr	62	19	-2.650	0.0081
L-AI	48	20	-4.173	<0.0001*
L-Cir	89	31	-5.477	<0.0001*
CD	131	25	-2.082	0.0369
UC	92	15	-1.943	0.0521
IBD-U	3	2	-1.732	0.2000 #
Auto-HSCT	28	5	1.508	0.1380 #
Allo-HSCT	49	41	-0.997	0.3186
CAR-T	4	4	-0.308	0.8286 #

Exact test p-values were used due to the small observation numbers.

Supplementary Table 5: Results table of Wilcoxon rank-sum test investigating differences in immune response following the second vaccination as quantified by the Roche anti-RBD Ig assay, in patients vaccinated with either ChAdOx1 nCoV-19 or BNT162b2 vaccines. Significant values after Bonferroni correction (alpha =0.003) are marked with an *.

	HC (210)		SC (79)		HM (22)		AAV (30)		IA (120)		HD (138)		HD on IS (12)		L-Tr (26)		L-AI (30)		L-Cir (30)		CD (49)		UC (53)		Auto-HSCT (15)		Allo-HSCT (62)		CAR-T (5)		Total (881)	
Sex																																
Male	100	(48%)	9	(11%)	14	(64%)	14	(47%)	40	(33%)	82	(59%)	4	(33%)	18	(69%)	10	(33%)	17	(57%)	32	(65%)	33	(62%)	6	(40%)	32	(52%)	3	(60%)	414	(47%)
Female	110	(52%)	70	(89%)	8	(36%)	16	(53%)	80	(67%)	56	(41%)	8	(67%)	8	(31%)	20	(67%)	13	(43%)	17	(35%)	20	(38%)	9	(60%)	30	(48%)	2	(40%)	467	(53%)
Age (years)																																
15-44	27	(13%)	18	(23%)	1	(5%)	9	(30%)	42	(35%)	17	(12%)	1	(8%)	5	(19%)	5	(17%)	4	(13%)	40	(82%)	33	(62%)	2	(13%)	15	(24%)	1	(20%)	220	(25%)
45-64	64	(30%)	34	(43%)	12	(55%)	13	(43%)	69	(57%)	48	(35%)	6	(50%)	14	(54%)	19	(63%)	19	(63%)	9	(18%)	16	(30%)	7	(47%)	33	(53%)	4	(80%)	367	(42%)
65-74	98	(47%)	20	(25%)	6	(27%)	4	(13%)	9	(8%)	40	(29%)	4	(33%)	7	(27%)	6	(20%)	7	(23%)	0	(0%)	3	(6%)	6	(40%)	14	(23%)	0	(0%)	224	(25%)
75+	21	(10%)	7	(9%)	3	(14%)	4	(13%)	0	(0%)	33	(24%)	1	(8%)	0	(0%)	0	(0%)	0	(0%)	0	(0%)	1	(2%)	0	(0%)	0	(0%)	0	(0%)	70	(8%)
Ethnicity																																
White	160	(76%)	56	(71%)	19	(86%)	26	(87%)	109	(91%)	40	(29%)	6	(50%)	25	(96%)	26	(87%)	28	(93%)	45	(92%)	45	(85%)	13	(87%)	59	(95%)	5	(100%)	662	(75%)
Black	12	(6%)	6	(8%)	0	(0%)	0	(0%)	1	(1%)	36	(26%)	4	(33%)	0	(0%)	0	(0%)	0	(0%)	0	(0%)	1	(2%)	1	(7%)	0	(0%)	0	(0%)	61	(7%)
Asian	19	(9%)	0	(0%)	2	(9%)	0	(0%)	0	(0%)	55	(40%)	2	(17%)	0	(0%)	3	(10%)	2	(7%)	3	(6%)	4	(8%)	0	(0%)	0	(0%)	0	(0%)	90	(10%)
Mixed/Other	10	(5%)	14	(18%)	0	(0%)	2	(7%)	10	(8%)	5	(4%)	0	(0%)	1	(4%)	1	(3%)	0	(0%)	1	(2%)	2	(4%)	0	(0%)	1	(2%)	0	(0%)	47	(5%)
Not Known	9	(4%)	3	(4%)	1	(5%)	2	(7%)	0	(0%)	2	(1%)	0	(0%)	0	(0%)	0	(0%)	0	(0%)	0	(0%)	1	(2%)	1	(7%)	2	(3%)	0	(0%)	21	(2%)
BMI																																
Underweight	0	(0%)	1	(1%)	0	(0%)	0	(0%)	1	(1%)	8	(6%)	1	(8%)	0	(0%)	2	(7%)	0	(0%)	1	(2%)	1	(2%)	0	(0%)	2	(3%)	1	(20%)	18	(2%)
Healthy Weight	13	(6%)	23	(29%)	7	(32%)	7	(23%)	31	(26%)	35	(25%)	4	(33%)	15	(58%)	11	(37%)	3	(10%)	34	(69%)	27	(51%)	5	(33%)	22	(35%)	2	(40%)	239	(27%)
Overweight	10	(5%)	15	(19%)	6	(27%)	8	(27%)	48	(40%)	31	(22%)	2	(17%)	3	(12%)	10	(33%)	13	(43%)	8	(16%)	17	(32%)	4	(27%)	14	(23%)	2	(40%)	191	(22%)
Obese	0	(0%)	19	(24%)	9	(41%)	15	(50%)	39	(33%)	42	(30%)	4	(33%)	8	(31%)	6	(20%)	13	(43%)	2	(4%)	6	(11%)	2	(13%)	8	(13%)	0	(0%)	173	(20%)
Unknown	10	(5%)	21	(27%)	0	(0%)	0	(0%)	1	(1%)	22	(16%)	1	(8%)	0	(0%)	1	(3%)	1	(3%)	4	(8%)	2	(4%)	4	(27%)	16	(26%)	0	(0%)	83	(9%)
Data Unavailable	177	(84%)	0	(0%)	0	(0%)	0	(0%)	0	(0%)	0	(0%)	0	(0%)	0	(0%)	0	(0%)	0	(0%)	0	(0%)	0	(0%)	0	(0%)	0	(0%)	0	(0%)	177	(20%)
Prior Covid																																
No Confirmed Infection	161	(77%)	66	(84%)	20	(91%)	28	(93%)	107	(89%)	66	(48%)	10	(83%)	23	(88%)	29	(97%)	24	(80%)	45	(92%)	47	(89%)	13	(87%)	49	(79%)	3	(60%)	691	(78%)
Yes	49	(23%)	13	(16%)	2	(9%)	2	(7%)	13	(11%)	72	(52%)	2	(17%)	3	(12%)	1	(3%)	6	(20%)	4	(8%)	6	(11%)	2	(13%)	13	(21%)	2	(40%)	190	(22%)
Vaccine Type																																
AstraZeneca	164	(78%)	27	(34%)	16	(73%)	30	(100%)	120	(100%)	86	(62%)	10	(83%)	26	(100%)	29	(97%)	30	(100%)	48	(98%)	50	(94%)	11	(73%)	31	(50%)	3	(60%)	681	(77%)
Pfizer	46	(22%)	48	(61%)	6	(27%)	0	(0%)	0	(0%)	52	(38%)	2	(17%)	0	(0%)	1	(3%)	0	(0%)	1	(2%)	3	(6%)	3	(20%)	26	(42%)	2	(40%)	190	(22%)
Moderna	0	(0%)	0	(0%)	0	(0%)	0	(0%)	0	(0%)	0	(0%)	0	(0%)	0	(0%)	0	(0%)	0	(0%)	0	(0%)	0	(0%)	0	(0%)	1	(2%)	0	(0%)	1	(0%)
Unknown	0	(0%)	4	(5%)	0	(0%)	0	(0%)	0	(0%)	0	(0%)	0	(0%)	0	(0%)	0	(0%)	0	(0%)	0	(0%)	0	(0%)	1	(7%)	4	(6%)	0	(0%)	9	(1%)
Smoking Status																																
Never Smoked	16	(8%)	18	(23%)	11	(50%)	15	(50%)	83	(69%)	36	(26%)	4	(33%)	15	(58%)	13	(43%)	12	(40%)	29	(59%)	37	(70%)	1	(7%)	17	(27%)	3	(60%)	310	(35%)
Previous Smoker	2	(1%)	13	(16%)	2	(9%)	8	(27%)	25	(21%)	15	(11%)	0	(0%)	11	(42%)	12	(40%)	11	(37%)	13	(27%)	10	(19%)	0	(0%)	7	(11%)	1	(20%)	130	(15%)
Current Smoker	0	(0%)	6	(8%)	0	(0%)	4	(13%)	12	(10%)	11	(8%)	0	(0%)	0	(0%)	5	(17%)	7	(23%)	6	(12%)	5	(9%)	0	(0%)	2	(3%)	0	(0%)	58	(7%)
Unknown	15	(7%)	42	(53%)	9	(41%)	3	(10%)	0	(0%)	76	(55%)	8	(67%)	0	(0%)	0	(0%)	0	(0%)	1	(2%)	1	(2%)	14	(93%)	36	(58%)	1	(20%)	206	(23%)
Data Unavailable	177	(84%)	0	(0%)	0	(0%)	0	(0%)	0	(0%)	0	(0%)	0	(0%)	0	(0%)	0	(0%)	0	(0%)	0	(0%)	0	(0%)	0	(0%)	0	(0%)	0	(0%)	177	(20%)
Diabetes																																
No	25	(12%)	70	(89%)	21	(95%)	24	(80%)	109	(91%)	72	(52%)	5	(42%)	23	(88%)	25	(83%)	16	(53%)	48	(98%)	52	(98%)	11	(73%)	59	(95%)	5	(100%)	565	(64%)
Yes	0	(0%)	6	(8%)	1	(5%)	5	(17%)	7	(6%)	66	(48%)	7	(58%)	3	(12%)	5	(17%)	14	(47%)	1	(2%)	1	(2%)	2	(13%)	3	(5%)	0	(0%)	121	(14%)
Not Known	8	(4%)	3	(4%)	0	(0%)	1	(3%)	4	(3%)	0	(0%)	0	(0%)	0	(0%)	0	(0%)	0	(0%)	0	(0%)	0	(0%)	2	(13%)	0	(0%)	0	(0%)	18	(2%)
Data Unavailable	177	(84%)	0	(0%)	0	(0%)	0	(0%)	0	(0%)	0	(0%)	0	(0%)	0	(0%)	0	(0%)	0	(0%)	0	(0%)	0	(0%)	0	(0%)	0	(0%)	0	(0%)	177	(20%)

Supplementary Table 6: Patient characteristics presented for patients in the Deep Immunophenotyping Group alongside matched healthy controls. Reported as N(%)

Disease Sub-group	Baseline (N)	Baseline	Pre-Boost (N)	Pre-Boost	Post-Boost (N)	Post-Boost
HC	113	0.4 (0.4, 0.4)	179	38.1 (15.3, 80.8)	192	1326.5 (608.5, 3856.0)
SC	11	0.4 (0.4, 590.0)	74	24.0 (6.2, 170.0)	67	3312.0 (566.0, 9741.0)
HM	0	. (., .)	21	20.5 (2.5, 75.9)	19	1409.0 (17.3, 5419.0)
AAV	6	0.4 (0.4, 1.3)	30	0.4 (0.4, 0.4)	29	0.4 (0.4, 24.5)
IA	109	0.4 (0.4, 0.4)	119	17.3 (5.6, 63.8)	115	338.0 (166.0, 835.0)
HD	135	0.4 (0.4, 453.0)	130	44.1 (0.6, 21114.0)	129	6046.0 (554.0, 24013.0)
HD on IS	11	0.4 (0.4, 0.8)	11	0.8 (0.4, 1.5)	12	798.0 (3.2, 3992.5)
L-Tr	6	0.4 (0.4, 0.4)	26	1.8 (0.4, 19.9)	26	68.2 (0.5, 446.0)
L-AI	4	0.4 (0.4, 0.4)	29	14.9 (1.5, 39.0)	27	440.0 (55.7, 1523.0)
L-Cir	8	0.4 (0.4, 0.4)	29	19.5 (3.0, 38.6)	27	1283.0 (484.0, 2979.0)
CD	49	0.4 (0.4, 0.4)	46	22.8 (6.5, 53.0)	42	559.5 (204.0, 911.0)
UC	52	0.4 (0.4, 0.4)	51	40.2 (18.6, 99.4)	48	1155.0 (664.5, 1969.5)
Auto-HSCT	1	0.4 (0.4, 0.4)	15	103.0 (15.1, 224.0)	13	1212.0 (645.0, 5129.0)
Allo-HSCT	15	1.1 (0.4, 34.5)	59	7.7 (0.4, 19.2)	47	517.0 (33.8, 5173.0)
CAR-T	1	0.4 (0.4, 0.4)	5	0.4 (0.4, 6.5)	5	903.0 (3.2, 8836.0)

Supplementary Table 7: Roche anti-SARS-CoV-2 RBD Ig Assay results presented using descriptive statistics, at pre- first vaccine (Baseline), pre-second vaccine (Pre-boost) & post- second vaccine (Post-boost), split by disease subgroup. Presented at each time point, Median Result (IQR)

Healthy Controls			Disease Sub-Group			
	**No Response, N(%)**	**Responder, N(%)**		**No Response, N(%)**	**Responder, N(%)**	**p-value**
Healthy Controls	2 (3.03%)	64 (96.97%)	OCTAVE	67 (11.55%)	513 (88.45%)	0.034
			SC	6 (9.52%)	57 (90.48%)	0.158
			HM	0 (0.00%)	17 (100.0)	1.00
			AAV	2 (6.90%)	27 (93.10%)	0.583
			IA	4 (3.48%)	111 (96.52%)	1.000
			HD	23 (18.55%)	101 (81.45%)	0.003*
			HD on IS	4 (33.33%)	8 (66.67%)	0.004
			L-Tr	6 (25.00%)	18 (75.00%)	0.004
			L-AI	2 (7.69%)	24 (92.31%)	0.316
			L-Cirr	2 (7.69%)	24 (92.31%)	0.316
			CD	0 (0.00%)	105 (100.00%)	0.523
			UC	4 (8.51%)	43 (91.49%)	0.232
			Auto-HSCT	2 (15.38%)	11 (84.62%)	0.124
			Allo-HSCT	11 (28.95%)	27 (71.05%)	<0.001*
			CAR-T	1 (20.00%)	4 (80.00%)	0.199

Supplementary Table 8: Chi Square (or Fisher's Exact where appropriate) to test for differences in proportions of responders and non-responders in disease sub-groups compared to healthy controls in IFNy ELISpot T Cell assay data. Significant values after Bonferroni correction (alpha =0.003) are marked with an *.

HC Obs number	Obs Number	Disease Group	Post-hoc Dunn's Test (z score)	p-value
90	63	SC	1.859	1.00
	17	HM	0.488	1.00
	29	AAV	-1.309	1.00
	115	IA	1.072	1.00
	24	L-Tr	3.821	0.004*
	26	L-AI	1.039	1.00
	26	L-Cir	1.161	1.00
	41	CD	1.724	1.00
	47	UC	1.207	1.00
	13	Auto-HSCT	1.082	1.00
	43	Allo-HSCT	3.339	0.03*
189	189	HD	3.011	0.22
	12	HD on IS		

Supplementary Table 9: Post-hoc Dunn's test (two-sided) to determine which sub-disease group(s) were different when compared to the healthy control group for ELISpot T Cell Assay (against full-spike or S1+S2 pools) response following the second vaccination (results include non-responders). P values are adjusted using Bonferroni correction, significant values are marked with an *.

Variable	OR	Lower CI (2.5%)	Upper CI (97.5%)	P value
45-64	0.92	0.7	1.2	0.53
65-74	0.71	0.52	0.98	0.0379
75+	0.73	0.47	1.12	0.1497
Black	1.27	0.75	2.16	0.3741
Asian	1.43	1.02	2.01	0.0398
Other	0.85	0.51	1.44	0.5499
SC	0.6	0.31	1.16	0.1276
HM	0.41	0.16	1.07	0.0684
AAV	0.03	0.01	0.13	<0.0001
IA	0.45	0.27	0.77	0.0031
HD	0.29	0.16	0.51	<0.0001
HD on IS	0.38	0.13	1.13	0.0819
K-Tr	0.26	0.12	0.57	0.0009
L-Tr	0.42	0.18	1.01	0.0525
L-AI	0.68	0.34	1.36	0.2739
L-Cir	1.23	0.65	2.33	0.5319
CD	0.42	0.23	0.76	0.0042
UC	0.87	0.44	1.7	0.675
IBD-U	0.44	0.06	3.18	0.4199
Auto-HSCT	0.44	0.18	1.05	0.0633
Allo-HSCT	0.25	0.14	0.46	<0.0001
CAR-T	0.03	0	0.2	0.0003
Vaccine Type Pfizer	2.99	2.33	3.84	<0.0001
Prior Covid	9.48	6	14.97	<0.0001
Anti-metabolites	0.32	0.22	0.47	<0.0001
Biologic Therapies	0.84	0.64	1.11	0.2306
Calcineurin Inhibitors	0.43	0.23	0.83	0.0116
Corticosteroids	0.64	0.47	0.88	0.0058
DMARDs	1.04	0.74	1.46	0.8155
Time Between Dose (wks)	1	1	1.01	0.3101

Supplementary Table 10: Multivariable logistic regression model presenting odds ratio of having an anti-RBD Ig response >380AU/mL after two doses of SARS-CoV-2 vaccine in all study participants and matched HC. SC: Solid cancer; HM: Haemotological malignancy; AAV: ANCA-associated vasculitis; IA: Inflammatory arthritis; HD: Haemodialysis; HD on IS: Haemodialysis on immunosuppression; L-Tr: Liver transplant; L-AI: Autoimmune hepatitis; L-Cir: Liver cirrhosis; CD: Crohn's disease; UC: Ulcerative colitis; Auto-HSCT: Autologous haematopoietic stem cell transplant; Allo-HSCT: Allogeneic haematopoietic stem cell transplant; CAR-T: Chimeric antigen receptor T cell; RBD = Receptor binding domain, Ig = immunoglobulin, DMARDs = Disease-modifying antirheumatic drugs, OR = Odds ratio; CI = Confidence interval. P<0.05 is significant

Variable	OR	Lower CI (2.5%)	Upper CI (97.5%)	P value
45-64	0.93	0.6	1.47	0.768
65-74	0.69	0.42	1.13	0.1395
75+	0.62	0.32	1.2	0.154
Black	0.88	0.42	1.82	0.7278
Asian	1.04	0.67	1.6	0.8722
Other	0.57	0.27	1.2	0.1373
SC	0.35	0.07	1.81	0.211
HM	0.16	0.03	1.06	0.0571
AAV	0.01	0	0.03	<0.0001
IA	0.32	0.08	1.31	0.1131
HD	0.17	0.04	0.66	0.0108
HD on IS	0.08	0.02	0.42	0.0028
K-Tr	0.09	0.02	0.37	0.0009
L-Tr	0.14	0.03	0.63	0.0106
L-AI	0.5	0.11	2.33	0.3751
L-Cir	1	-	-	-
CD	1	-	-	-
UC	1	-	-	-
IBD-U	0.07	0	0.92	0.0435
Auto-HSCT	0.06	0.01	0.28	0.0003
Allo-HSCT	0.11	0.03	0.42	0.0012
CAR-T	0	0	0.03	<0.0001
Vaccine Type Pfizer	1.43	1	2.03	0.0484
Prior Covid	10.34	3.99	26.83	<0.0001
Anti-metabolites	0.21	0.14	0.32	<0.0001
Biologic Therapies	1.24	0.83	1.85	0.2859
Calcineurin Inhibitors	0.5	0.23	1.09	0.0807
Corticosteroids	0.62	0.42	0.92	0.0179
DMARDs	0.8	0.34	1.85	0.5986
Time Between Dose (wks)	1	0.99	1.02	0.5334

Supplementary Table 11: Multivariable logistic regression model presenting odds ratio of having an anti-RBD Ig response >0.8AU/mL after two doses of SARS-CoV-2 vaccine in all study participants and matched HC. Age, ethnicity, the IA disease group and calcineurin inhibitor therapies were not associated with a decreased rate of seropositivity but were associated with a low serological response (Supp table 10). L-Cir, CD and UC groups were removed from this analysis as 100% of patients responded to vaccine. SC: Solid cancer; HM: Haemotological malignancy; AAV: ANCA-associated vasculitis; IA: Inflammatory arthritis; HD: Haemodialysis; HD on IS: Haemodialysis on immunosuppression; L-Tr: Liver transplant; L-AI: Autoimmune hepatitis; L-Cir: Liver cirrhosis; CD: Crohn’s disease; UC: Ulcerative colitis; Auto-HSCT: Autologous haematopoietic stem cell transplant; Allo-HSCT: Allogeneic haematopoietic stem cell transplant; CAR-T: Chimeric antigen receptor T cell; RBD = Receptor binding domain, Ig = immunoglobulin, DMARDs = Disease-modifying antirheumatic drugs, OR = Odds ratio; CI = Confidence interval. P<0.05 is significant

Variable	OR	Lower CI (2.5%)	Upper CI (97.5%)	P value
45-64	1.82	0.78	4.22	0.1653
65-74	0.9	0.37	2.18	0.8081
75+	1.54	0.43	5.53	0.5053
Black	0.67	0.19	2.34	0.5341
Asian	0.69	0.22	2.19	0.527
Other	1			
SC	0.45	0.09	2.34	0.3449
HM	1			
AAV	0.24	0.03	1.99	0.1842
IA	0.49	0.05	4.94	0.5433
HD	0.23	0.05	1	0.0502
HD on IS	0.86	0.05	14.54	0.9148
K-Tr				
L-Tr	0.26	0.02	2.91	0.2733
L-AI	0.46	0.05	4.39	0.501
L-Cir	0.2	0.03	1.45	0.1113
CD	1			
UC	0.34	0.04	2.65	0.3043
IBD-U				
Auto-HSCT	0.14	0.02	1.1	0.0611
Allo-HSCT	0.09	0.02	0.41	0.0019
CAR-T	0.07	0	1.08	0.0568
Vaccine Type Pfizer	0.23	0.11	0.51	0.0003
Prior Covid	4.05	1.5	10.9	0.0056
Anti-metabolites	0.7	0.15	3.27	0.6518
Biologic Therapies	1.07	0.41	2.8	0.8922
Calcineurin Inhibitors	0.18	0.03	1.06	0.058
Corticosteroids	0.81	0.34	1.92	0.6347
DMARDs	0.78	0.14	4.41	0.7749
Time Between Dose (wks)	0.99	0.87	1.13	0.8999

Supplementary Table 12: Multivariable logistic regression model presenting odds ratio of having an anti-SARS-CoV-2 spike T cell (≥4 SFC/10^6) after two doses of SARS-CoV-2 vaccine in all group 1 participants and matched HC. SC: Solid cancer; HM: Haematological malignancy; AAV: ANCA-associated vasculitis; IA: Inflammatory arthritis; HD: Haemodialysis; HD on IS: Haemodialysis on immunosuppression; L-Tr: Liver transplant; L-AI: Autoimmune hepatitis; L-Cir: Liver cirrhosis; CD: Crohn’s disease; UC: Ulcerative colitis; Auto-HSCT: Autologous haematopoietic stem cell transplant; Allo-HSCT: Allogeneic haematopoietic stem cell transplant; CAR-T: Chimeric antigen receptor T cell; RBD = Receptor binding domain, Ig = immunoglobulin, DMARDs = Disease-modifying antirheumatic drugs, OR = Odds ratio; CI = Confidence interval. $P<0.05$ is significant

Breakdown	Follow-up period	Responder status	Infection status	SC	HM	AAV	IA	HD	HD on IS	K-Tr	L-Tr	L-AI	L-Cirr	CD	UC	IBD-U	Auto-HSCT	Allo-HSCT	CAR-T	OCTAVE
				SARS-CoV-2 infection (Whole OCTAVE cohort)																
Serology breakdown	<6months post-V2	No response	Not Infected	0 (0%)	1 (50%)	1 (5%)	7 (28%)	7 (88%)	5 (83%)	128 (91%)	1 (5%)	1 (20%)	-	-	-	0 (0%)	0 (0%)	6 (46%)	0 (0%)	157 (62%)
			Infected	1 (33%)	0 (0%)	1 (5%)	1 (4%)	0 (0%)	0 (0%)	9 (6%)	0 (0%)	0 (0%)	-	-	-	0 (0%)	0 (0%)	2 (15%)	2 (50%)	16 (6%)
			Uk/NA	2 (67%)	1 (50%)	19 (90%)	17 (68%)	1 (13%)	1 (17%)	3 (2%)	19 (95%)	4 (80%)	-	-	-	1 (100%)	5 (100%)	5 (38%)	2 (50%)	80 (32%)
			Total#	3 (1%)	2 (1%)	21 (8%)	25 (10%)	8 (3%)	6 (2%)	140 (55%)	20 (8%)	5 (2%)	0 (0%)	0 (0%)	0 (0%)	1 (0%)	5 (2%)	13 (5%)	4 (2%)	253
		Low response	Not infected	3 (19%)	5 (71%)	0 (0%)	72 (35%)	31 (91%)	6 (86%)	131 (95%)	1 (4%)	1 (5%)	1 (6%)	11 (19%)	4 (17%)	0 (0%)	2 (33%)	15 (48%)	0 (0%)	283 (47%)
			Infected	1 (6%)	0 (0%)	0 (0%)	10 (5%)	1 (3%)	0 (0%)	5 (4%)	0 (0%)	2 (10%)	0 (0%)	2 (4%)	0 (0%)	0 (0%)	0 (0%)	5 (16%)	0 (0%)	26 (4%)
			Uk/NA	12 (75%)	2 (29%)	4 (100%)	125 (60%)	2 (6%)	1 (14%)	2 (1%)	26 (96%)	18 (86%)	16 (94%)	44 (77%)	20 (83%)	1 (100%)	4 (67%)	11 (35%)	1 (100%)	289 (48%)
			Total#	16 (3%)	7 (1%)	4 (1%)	207 (35%)	34 (6%)	7 (1%)	138 (23%)	27 (5%)	21 (4%)	17 (3%)	57 (10%)	24 (4%)	1 (0%)	6 (1%)	31 (5%)	1 (0%)	598
		High response	Not Infected	60 (76%)	11 (61%)	3 (100%)	409 (89%)	147 (95%)	16 (94%)	172 (97%)	17 (50%)	32 (76%)	66 (65%)	55 (56%)	54 (67%)	2 (67%)	19 (90%)	30 (59%)	2 (100%)	1095 (82%)
			Infected	7 (9%)	1 (6%)	0 (0%)	25 (5%)	3 (2%)	0 (0%)	4 (2%)	3 (9%)	1 (2%)	5 (5%)	13 (13%)	5 (6%)	0 (0%)	1 (5%)	3 (6%)	0 (0%)	71 (5%)
			Uk/NA	12 (15%)	6 (33%)	0 (0%)	23 (5%)	4 (3%)	1 (6%)	2 (1%)	14 (41%)	9 (21%)	30 (30%)	31 (31%)	22 (27%)	1 (33%)	1 (5%)	18 (35%)	0 (0%)	174 (13%)
			Total#	79 (6%)	18 (1%)	3 (0%)	457 (34%)	154 (11%)	17 (1%)	178 (13%)	34 (3%)	42 (3%)	101 (8%)	99 (7%)	81 (6%)	3 (0%)	21 (2%)	51 (4%)	2 (0%)	1,340
		Overall	Not Infected	63 (64%)	17 (63%)	4 (14%)	488 (71%)	185 (94%)	27 (90%)	431 (95%)	19 (23%)	34 (50%)	67 (57%)	66 (42%)	58 (55%)	2 (40%)	21 (66%)	51 (54%)	2 (29%)	1535 (70%)
			Infected	9 (9%)	1 (4%)	1 (4%)	36 (5%)	4 (2%)	0 (0%)	18 (4%)	3 (4%)	3 (4%)	5 (4%)	15 (10%)	5 (5%)	0 (0%)	1 (3%)	10 (11%)	2 (29%)	113 (5%)
			Uk/NA	26 (27%)	9 (33%)	23 (82%)	165 (24%)	7 (4%)	3 (10%)	7 (2%)	59 (73%)	31 (46%)	46 (39%)	75 (48%)	42 (40%)	3 (60%)	10 (31%)	34 (36%)	3 (43%)	543 (25%)
			Total#	98 (4%)	27 (1%)	28 (1%)	689 (31%)	196 (9%)	30 (1%)	456 (21%)	81 (4%)	68 (3%)	118 (5%)	156 (7%)	105 (5%)	5 (0%)	32 (1%)	95 (4%)	7 (0%)	2,191
	6months post-V2 - 12months post-V1	No response	Not Infected	1 (33%)	0 (0%)	1 (5%)	4 (16%)	4 (50%)	4 (67%)	93 (66%)	0 (0%)	0 (0%)	-	-	-	0 (0%)	0 (0%)	6 (46%)	1 (25%)	114 (45%)
			Infected	0 (0%)	1 (50%)	2 (10%)	2 (8%)	3 (38%)	2 (33%)	37 (26%)	1 (5%)	1 (20%)	-	-	-	0 (0%)	0 (0%)	1 (8%)	0 (0%)	50 (20%)
			Uk/NA	2 (67%)	1 (50%)	18 (86%)	19 (76%)	1 (13%)	0 (0%)	10 (7%)	19 (95%)	4 (80%)	-	-	-	1 (100%)	5 (100%)	6 (46%)	3 (75%)	89 (35%)
			Total#	3 (1%)	2 (1%)	21 (8%)	25 (10%)	8 (3%)	6 (2%)	140 (55%)	20 (8%)	5 (2%)	0 (0%)	0 (0%)	0 (0%)	1 (0%)	5 (2%)	13 (5%)	4 (2%)	253
		Low response	Not infected	2 (13%)	4 (57%)	0 (0%)	69 (33%)	21 (62%)	5 (71%)	101 (73%)	0 (0%)	1 (5%)	1 (6%)	7 (12%)	2 (8%)	0 (0%)	2 (33%)	15 (48%)	0 (0%)	230 (38%)
			Infected	1 (6%)	1 (14%)	0 (0%)	6 (3%)	6 (18%)	1 (14%)	31 (22%)	1 (4%)	2 (10%)	0 (0%)	14 (25%)	3 (13%)	0 (0%)	0 (0%)	2 (6%)	1 (100%)	69 (12%)
			Uk/NA	13 (81%)	2 (29%)	4 (100%)	132 (64%)	7 (21%)	1 (14%)	6 (4%)	26 (96%)	18 (86%)	16 (94%)	36 (63%)	19 (79%)	1 (100%)	4 (67%)	14 (45%)	0 (0%)	299 (50%)
			Total#	16 (3%)	7 (1%)	4 (1%)	207 (35%)	34 (6%)	7 (1%)	138 (23%)	27 (5%)	21 (4%)	17 (3%)	57 (10%)	24 (4%)	1 (0%)	6 (1%)	31 (5%)	1 (0%)	598
		High response	Not Infected	46 (58%)	11 (61%)	3 (100%)	392 (86%)	110 (71%)	9 (53%)	136 (76%)	12 (35%)	24 (57%)	53 (52%)	39 (39%)	28 (35%)	2 (67%)	20 (95%)	25 (49%)	2 (100%)	912 (68%)
			Infected	8 (10%)	0 (0%)	0 (0%)	35 (8%)	30 (19%)	6 (35%)	37 (21%)	9 (26%)	9 (21%)	27 (27%)	38 (38%)	35 (43%)	1 (33%)	1 (5%)	6 (12%)	0 (0%)	242 (18%)
			Uk/NA	25 (32%)	7 (39%)	0 (0%)	30 (7%)	14 (9%)	2 (12%)	5 (3%)	13 (38%)	9 (21%)	21 (21%)	22 (22%)	18 (22%)	0 (0%)	0 (0%)	20 (39%)	0 (0%)	186 (14%)
			Total#	79 (6%)	18 (1%)	3 (0%)	457 (34%)	154 (11%)	17 (1%)	178 (13%)	34 (3%)	42 (3%)	101 (8%)	99 (7%)	81 (6%)	3 (0%)	21 (2%)	51 (4%)	2 (0%)	1340
		Overall	Not Infected	49 (50%)	15 (56%)	4 (14%)	465 (67%)	135 (69%)	18 (60%)	330 (72%)	12 (15%)	25 (37%)	54 (46%)	46 (29%)	30 (29%)	2 (40%)	22 (69%)	46 (48%)	3 (43%)	1256 (57%)
			Infected	9 (9%)	2 (7%)	2 (7%)	43 (6%)	39 (20%)	9 (30%)	105 (23%)	11 (14%)	12 (18%)	27 (23%)	52 (33%)	38 (36%)	1 (20%)	1 (3%)	9 (9%)	1 (14%)	361 (16%)
			Uk/NA	40 (41%)	10 (37%)	22 (79%)	181 (26%)	22 (11%)	3 (10%)	21 (5%)	58 (72%)	31 (46%)	37 (31%)	58 (37%)	37 (35%)	2 (40%)	9 (28%)	40 (42%)	3 (43%)	574 (26%)
			Total#	98 (4%)	27 (1%)	28 (1%)	689 (31%)	196 (9%)	30 (1%)	456 (21%)	81 (4%)	68 (3%)	118 (5%)	156 (7%)	105 (5%)	5 (0%)	32 (1%)	95 (4%)	7 (0%)	2191
T cell Breakdown	<6months post-V2	No response	Not Infected	4 (67%)	-	0 (0%)	2 (50%)	21 (95%)	4 (100%)	-	1 (17%)	1 (50%)	2 (100%)	-	1 (25%)	-	2 (100%)	6 (55%)	0 (0%)	44 (67%)
			Infected	0 (0%)	-	0 (0%)	0 (0%)	0 (0%)	0 (0%)	-	0 (0%)	0 (0%)	0 (0%)	-	0 (0%)	-	0 (0%)	2 (18%)	0 (0%)	2 (3%)
			Uk/NA	2 (33%)	-	2 (100%)	2 (50%)	1 (5%)	0 (0%)	-	5 (83%)	1 (50%)	0 (0%)	-	3 (75%)	-	0 (0%)	3 (27%)	1 (100%)	20 (30%)
			Total#	6 (9%)	0 (0%)	2 (3%)	4 (6%)	22 (33%)	4 (6%)	-	6 (9%)	2 (3%)	2 (3%)	0 (0%)	4 (6%)	-	2 (3%)	11 (17%)	1 (2%)	66
		Yes response	Not infected	37 (65%)	13 (76%)	4 (15%)	47 (43%)	94 (94%)	8 (100%)	-	6 (33%)	13 (54%)	10 (42%)	22 (54%)	26 (63%)	-	8 (73%)	19 (70%)	2 (67%)	309 (61%)
			Infected	4 (7%)	1 (6%)	1 (4%)	8 (7%)	2 (2%)	0 (0%)	-	1 (6%)	0 (0%)	3 (13%)	1 (2%)	2 (5%)	-	0 (0%)	1 (4%)	0 (0%)	24 (5%)
			Uk/NA	16 (28%)	3 (18%)	21 (81%)	55 (50%)	4 (4%)	0 (0%)	-	11 (61%)	11 (46%)	11 (46%)	18 (44%)	13 (32%)	-	3 (27%)	7 (26%)	1 (33%)	174 (34%)
			Total#	57 (11%)	17 (3%)	26 (5%)	110 (22%)	100 (20%)	8 (2%)	-	18 (4%)	24 (5%)	24 (5%)	41 (8%)	41 (8%)	-	11 (2%)	27 (5%)	3 (1%)	507
		Overall	Not Infected	41 (65%)	13 (76%)	4 (14%)	49 (43%)	115 (94%)	12 (100%)	-	7 (29%)	14 (54%)	12 (46%)	22 (54%)	27 (60%)	-	10 (77%)	25 (66%)	2 (50%)	353 (62%)
			Infected	4 (6%)	1 (6%)	1 (4%)	8 (7%)	2 (2%)	0 (0%)	-	1 (4%)	0 (0%)	3 (12%)	1 (2%)	2 (4%)	-	0 (0%)	3 (8%)	0 (0%)	26 (5%)
			Uk/NA	18 (29%)	3 (18%)	23 (82%)	57 (50%)	5 (4%)	0 (0%)	-	16 (67%)	12 (46%)	11 (42%)	18 (44%)	16 (36%)	-	3 (23%)	10 (26%)	2 (50%)	194 (34%)
			Total#	63 (11%)	17 (3%)	28 (5%)	114 (20%)	122 (21%)	12 (2%)	-	24 (4%)	26 (5%)	26 (5%)	41 (7%)	45 (8%)	-	13 (2%)	38 (7%)	4 (1%)	573
	6months post-V2 - 12months post-V1	No response	Not Infected	3 (50%)	-	0 (0%)	0 (0%)	12 (55%)	2 (50%)	-	0 (0%)	1 (50%)	2 (100%)	-	0 (0%)	-	2 (100%)	7 (64%)	0 (0%)	29 (44%)
			Infected	0 (0%)	-	0 (0%)	2 (50%)	8 (36%)	2 (50%)	-	1 (17%)	0 (0%)	0 (0%)	-	1 (25%)	-	0 (0%)	1 (9%)	1 (100%)	16 (24%)
			Uk/NA	3 (50%)	-	2 (100%)	2 (50%)	2 (9%)	0 (0%)	-	5 (83%)	1 (50%)	0 (0%)	-	3 (75%)	-	0 (0%)	3 (27%)	0 (0%)	21 (32%)
			Total#	6 (9%)	0 (0%)	2 (3%)	4 (6%)	22 (33%)	4 (6%)	-	6 (9%)	2 (3%)	2 (3%)	0 (0%)	4 (6%)	-	2 (3%)	11 (17%)	1 (2%)	66
		Yes response	Not infected	30 (53%)	12 (71%)	4 (15%)	45 (41%)	76 (76%)	5 (63%)	-	5 (28%)	7 (29%)	8 (33%)	13 (32%)	14 (34%)	-	8 (73%)	17 (63%)	2 (67%)	246 (49%)
			Infected	4 (7%)	1 (6%)	2 (8%)	10 (9%)	11 (11%)	3 (38%)	-	2 (11%)	5 (21%)	4 (17%)	10 (24%)	14 (34%)	-	1 (9%)	1 (4%)	0 (0%)	68 (13%)
			Uk/NA	23 (40%)	4 (24%)	20 (77%)	55 (50%)	13 (13%)	0 (0%)	-	11 (61%)	12 (50%)	12 (50%)	18 (44%)	13 (32%)	-	2 (18%)	9 (33%)	1 (33%)	193 (38%)
			Total#	57 (11%)	17 (3%)	26 (5%)	110 (22%)	100 (20%)	8 (2%)	-	18 (4%)	24 (5%)	24 (5%)	41 (8%)	41 (8%)	-	11 (2%)	27 (5%)	3 (1%)	507
		Overall	Not Infected	33 (52%)	12 (71%)	4 (14%)	45 (39%)	88 (72%)	7 (58%)	-	5 (21%)	8 (31%)	10 (38%)	13 (32%)	14 (31%)	-	10 (77%)	24 (63%)	2 (50%)	275 (48%)
			Infected	4 (6%)	1 (6%)	2 (7%)	12 (11%)	19 (16%)	5 (42%)	-	3 (13%)	5 (19%)	4 (15%)	10 (24%)	15 (33%)	-	1 (8%)	2 (5%)	1 (25%)	84 (15%)
			Uk/NA	26 (41%)	4 (24%)	22 (79%)	57 (50%)	15 (12%)	0 (0%)	-	16 (67%)	13 (50%)	12 (46%)	18 (44%)	16 (36%)	-	2 (15%)	12 (32%)	1 (25%)	214 (37%)
			Total#	63 (11%)	17 (3%)	28 (5%)	114 (20%)	122 (21%)	12 (2%)	-	24 (4%)	26 (5%)	26 (5%)	41 (7%)	45 (8%)	-	13 (2%)	38 (7%)	4 (1%)	573

Supplementary Table 13. Summary of reported SARS-CoV-2 infections following vaccination for immunocompromised patients in OCTAVE, split by disease subgroup, follow-up timepoint and on Roche anti-RBD Ig assay result ≥0.8AU/mL and low response <380AU/mL or IFNy ELISpot response. No response = $0SFC/10^6$, Yes response $>0SFC/10^6$. * K-Tr and IBD-U groups were not included in group 1. [#] % of total OCTAVE infections. Uk : Unknown – infection status defined as unknown in OCTAVE database. NA: Not available – no data entry in OCTAVE database or patient was moved into OCTAVE DUO trial.

SC: Solid cancer; HM: Haematological malignancy; AAV: ANCA-associated vasculitis; IA: Inflammatory arthritis; HD: Haemodialysis; HD on IS: Haemodialysis on immunosuppression; L-Tr: Liver transplant; L-AI: Autoimmune hepatitis; L-Cir: Liver cirrhosis; CD: Crohn's disease; UC: Ulcerative colitis; Auto-HSCT: Autologous haematopoietic stem cell transplant; Allo-HSCT: Allogeneic haematopoietic stem cell transplant; CAR-T: Chimeric antigen receptor T cell

SARS-CoV-2 infection (COVID-19 naïve at baseline)

	Follow-up period	Responder status	Infection status	SC	HM	AAV	IA	HD	HD on IS	K-Tr	L-Tr	L-AI	L-Cirr	CD	UC	IBD-U	Auto-HSCT	Allo-HSCT	CAR-T	OCTAVE
Serology breakdown	<6months post-V2	No response	Not Infected	0 (0%)	1 (50%)	1 (5%)	7 (28%)	6 (86%)	5 (83%)	126 (92%)	1 (5%)	1 (20%)	-	-	-	0 (0%)	0 (0%)	6 (46%)	0 (0%)	154 (62%)
			Infected	1 (33%)	0 (0%)	1 (5%)	1 (4%)	0 (0%)	0 (0%)	9 (7%)	0 (0%)	0 (0%)	-	-	-	0 (0%)	0 (0%)	2 (15%)	1 (33%)	15 (6%)
			Uk/NA	2 (67%)	1 (50%)	19 (90%)	17 (68%)	1 (14%)	1 (17%)	2 (1%)	19 (95%)	4 (80%)	-	-	-	1 (100%)	5 (100%)	5 (38%)	2 (67%)	79 (32%)
			Total#	3 (1%)	2 (1%)	21 (8%)	25 (10%)	7 (3%)	6 (2%)	137 (55%)	20 (8%)	5 (2%)	0 (0%)	0 (0%)	0 (0%)	1 (0%)	5 (2%)	13 (5%)	3 (1%)	248
		Low response	Not infected	2 (14%)	5 (71%)	0 (0%)	71 (35%)	28 (90%)	6 (86%)	127 (95%)	1 (4%)	1 (5%)	1 (6%)	10 (18%)	4 (17%)	0 (0%)	2 (33%)	14 (47%)	0 (0%)	272 (47%)
			Infected	0 (0%)	0 (0%)	0 (0%)	9 (4%)	1 (3%)	0 (0%)	5 (4%)	0 (0%)	2 (10%)	0 (0%)	2 (4%)	0 (0%)	0 (0%)	0 (0%)	5 (17%)	0 (0%)	24 (4%)
			Uk/NA	12 (86%)	2 (29%)	3 (100%)	122 (60%)	2 (6%)	1 (14%)	2 (1%)	26 (96%)	18 (86%)	15 (94%)	44 (79%)	20 (83%)	1 (100%)	4 (67%)	11 (37%)	1 (100%)	284 (49%)
			Total#	14 (2%)	7 (1%)	3 (1%)	202 (35%)	31 (5%)	7 (1%)	134 (23%)	27 (5%)	21 (4%)	16 (3%)	56 (10%)	24 (4%)	1 (0%)	6 (1%)	30 (5%)	1 (0%)	580
		High response	Not Infected	51 (77%)	11 (61%)	3 (100%)	373 (89%)	88 (96%)	9 (100%)	139 (97%)	15 (50%)	30 (75%)	63 (68%)	50 (55%)	49 (67%)	1 (50%)	17 (89%)	29 (59%)	1 (100%)	929 (81%)
			Infected	6 (9%)	1 (6%)	0 (0%)	24 (6%)	2 (2%)	0 (0%)	2 (1%)	2 (7%)	1 (3%)	4 (4%)	13 (14%)	4 (5%)	0 (0%)	1 (5%)	3 (6%)	0 (0%)	63 (5%)
			Uk/NA	9 (14%)	6 (33%)	0 (0%)	22 (5%)	2 (2%)	0 (0%)	2 (1%)	13 (43%)	9 (23%)	26 (28%)	28 (31%)	20 (27%)	1 (50%)	1 (5%)	17 (35%)	0 (0%)	156 (14%)
			Total#	66 (6%)	18 (2%)	3 (0%)	419 (36%)	92 (8%)	9 (1%)	143 (12%)	30 (3%)	40 (3%)	93 (8%)	91 (8%)	73 (6%)	2 (0%)	19 (2%)	49 (4%)	1 (0%)	1148
		Overall	Not Infected	53 (64%)	17 (63%)	4 (15%)	451 (70%)	122 (94%)	20 (91%)	392 (95%)	17 (22%)	32 (48%)	64 (59%)	60 (41%)	53 (55%)	1 (25%)	19 (63%)	49 (53%)	1 (20%)	1355 (69%)
			Infected	7 (8%)	1 (4%)	1 (4%)	34 (5%)	3 (2%)	0 (0%)	16 (4%)	2 (3%)	3 (5%)	4 (4%)	15 (10%)	4 (4%)	0 (0%)	1 (3%)	10 (11%)	1 (20%)	102 (5%)
			Uk/NA	23 (28%)	9 (33%)	22 (81%)	161 (25%)	5 (4%)	2 (9%)	6 (1%)	58 (75%)	31 (47%)	41 (38%)	72 (49%)	40 (41%)	3 (75%)	10 (33%)	33 (36%)	3 (60%)	519 (26%)
			Total#	83 (4%)	27 (1%)	27 (1%)	646 (33%)	130 (7%)	22 (1%)	414 (21%)	77 (4%)	66 (3%)	109 (6%)	147 (7%)	97 (5%)	4 (0%)	30 (2%)	92 (5%)	5 (0%)	1976
	6months post-V2 - 12months post-V1	No response	Not Infected	1 (33%)	0 (0%)	1 (5%)	4 (16%)	3 (43%)	4 (67%)	93 (68%)	0 (0%)	0 (0%)	-	-	-	0 (0%)	0 (0%)	6 (46%)	1 (33%)	113 (46%)
			Infected	0 (0%)	1 (50%)	2 (10%)	2 (8%)	3 (43%)	2 (33%)	36 (26%)	1 (5%)	1 (20%)	-	-	-	0 (0%)	0 (0%)	1 (8%)	0 (0%)	49 (20%)
			Uk/NA	2 (67%)	1 (50%)	18 (86%)	19 (76%)	1 (14%)	0 (0%)	8 (6%)	19 (95%)	4 (80%)	-	-	-	1 (100%)	5 (100%)	6 (46%)	2 (67%)	86 (35%)
			Total#	3 (1%)	2 (1%)	21 (8%)	25 (10%)	7 (3%)	6 (2%)	137 (55%)	20 (8%)	5 (2%)	0 (0%)	0 (0%)	0 (0%)	1 (0%)	5 (2%)	13 (5%)	3 (1%)	248
		Low response	Not infected	1 (7%)	4 (57%)	0 (0%)	68 (34%)	18 (58%)	5 (71%)	98 (73%)	0 (0%)	1 (5%)	1 (6%)	6 (11%)	2 (8%)	0 (0%)	2 (33%)	14 (47%)	0 (0%)	220 (38%)
			Infected	0 (0%)	1 (14%)	0 (0%)	6 (3%)	6 (19%)	1 (14%)	30 (22%)	1 (4%)	2 (10%)	0 (0%)	14 (25%)	3 (13%)	0 (0%)	0 (0%)	2 (7%)	1 (100%)	67 (12%)
			Uk/NA	13 (93%)	2 (29%)	3 (100%)	128 (63%)	7 (23%)	1 (14%)	6 (4%)	26 (96%)	18 (86%)	15 (94%)	36 (64%)	19 (79%)	1 (100%)	4 (67%)	14 (47%)	0 (0%)	293 (51%)
			Total#	14 (2%)	7 (1%)	3 (1%)	202 (35%)	31 (5%)	7 (1%)	134 (23%)	27 (5%)	21 (4%)	16 (3%)	56 (10%)	24 (4%)	1 (0%)	6 (1%)	30 (5%)	1 (0%)	580
		High response	Not Infected	40 (61%)	11 (61%)	3 (100%)	359 (86%)	63 (68%)	4 (44%)	107 (75%)	10 (33%)	23 (58%)	49 (53%)	36 (40%)	24 (33%)	1 (50%)	18 (95%)	24 (49%)	1 (100%)	773 (67%)
			Infected	6 (9%)	0 (0%)	0 (0%)	32 (8%)	20 (22%)	4 (44%)	31 (22%)	8 (27%)	8 (20%)	26 (28%)	36 (40%)	34 (47%)	1 (50%)	1 (5%)	6 (12%)	0 (0%)	213 (19%)
			Uk/NA	20 (30%)	7 (39%)	0 (0%)	28 (7%)	9 (10%)	1 (11%)	5 (3%)	12 (40%)	9 (23%)	18 (19%)	19 (21%)	15 (21%)	0 (0%)	0 (0%)	19 (39%)	0 (0%)	162 (14%)
			Total#	66 (6%)	18 (2%)	3 (0%)	419 (36%)	92 (8%)	9 (1%)	143 (12%)	30 (3%)	40 (3%)	93 (8%)	91 (8%)	73 (6%)	2 (0%)	19 (2%)	49 (4%)	1 (0%)	1148
		Overall	Not Infected	42 (51%)	15 (56%)	4 (15%)	431 (67%)	84 (65%)	13 (59%)	298 (72%)	10 (13%)	24 (36%)	50 (46%)	42 (29%)	26 (27%)	1 (25%)	20 (67%)	44 (48%)	2 (40%)	1106 (56%)
			Infected	6 (7%)	2 (7%)	2 (7%)	40 (6%)	29 (22%)	7 (32%)	97 (23%)	10 (13%)	11 (17%)	26 (24%)	50 (34%)	37 (38%)	1 (25%)	1 (3%)	9 (10%)	1 (20%)	329 (17%)
			Uk/NA	35 (42%)	10 (37%)	21 (78%)	175 (27%)	17 (13%)	2 (9%)	19 (5%)	57 (74%)	31 (47%)	33 (30%)	55 (37%)	34 (35%)	2 (50%)	9 (30%)	39 (42%)	2 (40%)	541 (27%)
			Total#	83 (4%)	27 (1%)	27 (1%)	646 (33%)	130 (7%)	22 (1%)	414 (21%)	77 (4%)	66 (3%)	109 (6%)	147 (7%)	97 (5%)	4 (0%)	30 (2%)	92 (5%)	5 (0%)	1976
T cell Breakdown	<6months post-V2	No response	Not Infected	3 (60%)	-	0 (0%)	2 (50%)	17 (94%)	4 (100%)	-	1 (17%)	1 (50%)	2 (100%)	-	1 (25%)	-	2 (100%)	6 (55%)	0 (0%)	39 (64%)
			Infected	0 (0%)	-	0 (0%)	0 (0%)	0 (0%)	0 (0%)	-	0 (0%)	0 (0%)	0 (0%)	-	0 (0%)	-	0 (0%)	2 (18%)	0 (0%)	2 (3%)
			Uk/NA	2 (40%)	-	2 (100%)	2 (50%)	1 (6%)	0 (0%)	-	5 (83%)	1 (50%)	0 (0%)	-	3 (75%)	-	0 (0%)	3 (27%)	1 (100%)	20 (33%)
			Total#	5 (8%)	0 (0%)	2 (3%)	4 (7%)	18 (30%)	4 (7%)	0 (0%)	6 (10%)	2 (3%)	2 (3%)	0 (0%)	4 (7%)	0 (0%)	2 (3%)	11 (18%)	1 (2%)	61
		Yes response	Not infected	32 (64%)	13 (76%)	4 (16%)	41 (40%)	53 (93%)	6 (100%)	-	4 (25%)	13 (54%)	10 (45%)	22 (55%)	25 (64%)	-	7 (70%)	17 (68%)	1 (50%)	248 (57%)
			Infected	4 (8%)	1 (6%)	1 (4%)	8 (8%)	2 (4%)	0 (0%)	-	1 (6%)	0 (0%)	3 (14%)	1 (3%)	2 (5%)	-	0 (0%)	1 (4%)	0 (0%)	24 (6%)
			Uk/NA	14 (28%)	3 (18%)	20 (80%)	54 (52%)	2 (4%)	0 (0%)	-	11 (69%)	11 (46%)	9 (41%)	17 (43%)	12 (31%)	-	3 (30%)	7 (28%)	1 (50%)	164 (38%)
			Total#	50 (11%)	17 (4%)	25 (6%)	103 (24%)	57 (13%)	6 (1%)	0 (0%)	16 (4%)	24 (6%)	22 (5%)	40 (9%)	39 (9%)	0 (0%)	10 (2%)	25 (6%)	2 (0%)	436
		Overall	Not Infected	35 (64%)	13 (76%)	4 (15%)	43 (40%)	70 (93%)	10 (100%)	-	5 (23%)	14 (54%)	12 (50%)	22 (55%)	26 (60%)	-	9 (75%)	23 (64%)	1 (33%)	287 (58%)
			Infected	4 (7%)	1 (6%)	1 (4%)	8 (7%)	2 (3%)	0 (0%)	-	1 (5%)	0 (0%)	3 (13%)	1 (3%)	2 (5%)	-	0 (0%)	3 (8%)	0 (0%)	26 (5%)
			Uk/NA	16 (29%)	3 (18%)	22 (81%)	56 (52%)	3 (4%)	0 (0%)	-	16 (73%)	12 (46%)	9 (38%)	17 (43%)	15 (35%)	-	3 (25%)	10 (28%)	2 (67%)	184 (37%)
			Total#	55 (11%)	17 (3%)	27 (5%)	107 (22%)	75 (15%)	10 (2%)	0 (0%)	22 (4%)	26 (5%)	24 (5%)	40 (8%)	43 (9%)	0 (0%)	12 (2%)	36 (7%)	3 (1%)	497
	6months post-V2 - 12months post-V1	No response	Not Infected	2 (40%)	-	0 (0%)	0 (0%)	9 (50%)	2 (50%)	-	0 (0%)	1 (50%)	2 (100%)	-	0 (0%)	-	2 (100%)	7 (64%)	0 (0%)	25 (41%)
			Infected	0 (0%)	-	0 (0%)	2 (50%)	7 (39%)	2 (50%)	-	1 (17%)	0 (0%)	0 (0%)	-	1 (25%)	-	0 (0%)	1 (9%)	1 (100%)	15 (25%)
			Uk/NA	3 (60%)	-	2 (100%)	2 (50%)	2 (11%)	0 (0%)	-	5 (83%)	1 (50%)	0 (0%)	-	3 (75%)	-	0 (0%)	3 (27%)	0 (0%)	21 (34%)
			Total#	5 (8%)	0 (0%)	2 (3%)	4 (7%)	18 (30%)	4 (7%)	0 (0%)	6 (10%)	2 (3%)	2 (3%)	0 (0%)	4 (7%)	0 (0%)	2 (3%)	11 (18%)	1 (2%)	61
		Yes response	Not infected	27 (54%)	12 (71%)	4 (16%)	39 (38%)	42 (74%)	3 (50%)	-	4 (25%)	7 (29%)	8 (36%)	13 (33%)	13 (33%)	-	7 (70%)	15 (60%)	1 (50%)	195 (45%)
			Infected	3 (6%)	1 (6%)	2 (8%)	10 (10%)	6 (11%)	3 (50%)	-	1 (6%)	5 (21%)	4 (18%)	10 (25%)	14 (36%)	-	1 (10%)	1 (4%)	0 (0%)	61 (14%)
			Uk/NA	20 (40%)	4 (24%)	19 (76%)	54 (52%)	9 (16%)	0 (0%)	-	11 (69%)	12 (50%)	10 (45%)	17 (43%)	12 (31%)	-	2 (20%)	9 (36%)	1 (50%)	180 (41%)
			Total#	50 (11%)	17 (4%)	25 (6%)	103 (24%)	57 (13%)	6 (1%)	0 (0%)	16 (4%)	24 (6%)	22 (5%)	40 (9%)	39 (9%)	0 (0%)	10 (2%)	25 (6%)	2 (0%)	436
		Overall	Not Infected	29 (53%)	12 (71%)	4 (15%)	39 (36%)	51 (68%)	5 (50%)	-	4 (18%)	8 (31%)	10 (42%)	13 (33%)	13 (30%)	-	9 (75%)	22 (61%)	1 (33%)	220 (44%)
			Infected	3 (5%)	1 (6%)	2 (7%)	12 (11%)	13 (17%)	5 (50%)	-	2 (9%)	5 (19%)	4 (17%)	10 (25%)	15 (35%)	-	1 (8%)	2 (6%)	1 (33%)	76 (15%)
			Uk/NA	23 (42%)	4 (24%)	21 (78%)	56 (52%)	11 (15%)	0 (0%)	-	16 (73%)	13 (50%)	10 (42%)	17 (43%)	15 (35%)	-	2 (17%)	12 (33%)	1 (33%)	201 (40%)
			Total#	55 (11%)	17 (3%)	27 (5%)	107 (22%)	75 (15%)	10 (2%)	0 (0%)	22 (4%)	26 (5%)	24 (5%)	40 (8%)	43 (9%)	0 (0%)	12 (2%)	36 (7%)	3 (1%)	497

Supplementary Table 14. Summary of reported SARS-CoV-2 infections following vaccination for immunocompromised patients in OCTAVE that were SARS-CoV-2 naïve at baseline, split by disease subgroup, follow-up timepoint and on Roche anti-RBD Ig assay result ≥0.8AU/mL and low response <380AU/mL or IFNγ ELISpot response. No response = 0SFC/10^6, Yes response >0SFC/10^6. Uk : Unknown – infection status defined as unknown in OCTAVE database. NA: Not available – no data entry in OCTAVE database or patient was moved into OCTAVE DUO trial. * K-Tr and IBD-U groups were not included in group 1. # % of total OCTAVE infections

SC: Solid cancer; HM: Haematological malignancy; AAV: ANCA-associated vasculitis; IA: Inflammatory arthritis; HD: Haemodialysis; HD on IS: Haemodialysis on immunosuppression; L-Tr: Liver transplant; L-AI: Autoimmune hepatitis; L-Cir: Liver cirrhosis; CD: Crohn's disease; UC: Ulcerative colitis; Auto-HSCT: Autologous haematopoietic stem cell transplant; Allo-HSCT: Allogeneic haematopoietic stem cell transplant; CAR-T: Chimeric antigen receptor T cell

		COVID-19 naïve at baseline : Anti-RBD Ig breakdown - <6months post-v2 - 12months post-V1 (n (%))																
		SC	HM	AAV	IA	HD	HD on IS	K-Tr	L-Tr	L-AI	L-Cirr	CD	UC	IBD-U	Auto-HSCT	Allo-HSCT	CAR-T	OCTAVE
No Response	Asymptomatic	0 (0%)	0 (0%)	0 (0%)	0 (0%)	0 (0%)	1 (50%)	5 (11%)	0 (0%)	0 (0%)	-	-	-	-	-	0 (0%)	0 (0%)	6 (9%)
	Symptomatic	1 (100%)	1 (100%)	2 (67%)	1 (33%)	2 (67%)	0 (0%)	24 (53%)	1 (100%)	1 (100%)	-	-	-	-	-	1 (33%)	0 (0%)	34 (53%)
	Hospitalised - No Oxygen Required	0 (0%)	0 (0%)	0 (0%)	0 (0%)	0 (0%)	0 (0%)	3 (7%)	0 (0%)	0 (0%)	-	-	-	-	-	0 (0%)	1 (100%)	4 (6%)
	Hospitalised - Oxygen Required	0 (0%)	0 (0%)	0 (0%)	1 (33%)	0 (0%)	0 (0%)	8 (18%)	0 (0%)	0 (0%)	-	-	-	-	-	0 (0%)	0 (0%)	9 (14%)
	ITU	0 (0%)	0 (0%)	0 (0%)	1 (33%)	0 (0%)	0 (0%)	0 (0%)	0 (0%)	0 (0%)	-	-	-	-	-	0 (0%)	0 (0%)	1 (2%)
	COVID-19 related mortality	0 (0%)	0 (0%)	1 (33%)	0 (0%)	1 (33%)	1 (50%)	2 (4%)	0 (0%)	0 (0%)	-	-	-	-	-	0 (0%)	0 (0%)	5 (8%)
	Unknown/NA	0 (0%)	0 (0%)	0 (0%)	0 (0%)	0 (0%)	0 (0%)	3 (7%)	0 (0%)	0 (0%)	-	-	-	-	-	2 (67%)	0 (0%)	5 (8%)
	Total[#]	1 (2%)	1 (2%)	3 (5%)	3 (5%)	3 (5%)	2 (3%)	45 (70%)	1 (2%)	1 (2%)	0 (0%)	0 (0%)	0 (0%)	0 (0%)	0 (0%)	3 (5%)	1 (2%)	64
Low response	Asymptomatic	-	0 (0%)	-	0 (0%)	2 (29%)	0 (0%)	5 (14%)	0 (0%)	0 (0%)	-	0 (0%)	0 (0%)	-	-	1 (14%)	0 (0%)	8 (9%)
	Symptomatic	-	0 (0%)	-	12 (80%)	4 (57%)	0 (0%)	23 (66%)	1 (100%)	3 (75%)	-	15 (94%)	3 (100%)	-	-	4 (57%)	0 (0%)	65 (71%)
	Hospitalised - No Oxygen Required	-	0 (0%)	-	1 (7%)	0 (0%)	0 (0%)	5 (14%)	0 (0%)	0 (0%)	-	0 (0%)	0 (0%)	-	-	0 (0%)	0 (0%)	6 (7%)
	Hospitalised - Oxygen Required	-	0 (0%)	-	1 (7%)	1 (14%)	0 (0%)	1 (3%)	0 (0%)	0 (0%)	-	0 (0%)	0 (0%)	-	-	0 (0%)	0 (0%)	3 (3%)
	ITU	-	0 (0%)	-	0 (0%)	0 (0%)	0 (0%)	0 (0%)	0 (0%)	0 (0%)	-	0 (0%)	0 (0%)	-	-	1 (14%)	0 (0%)	1 (1%)
	COVID-19 related mortality	-	0 (0%)	-	0 (0%)	0 (0%)	1 (100%)	1 (3%)	0 (0%)	0 (0%)	-	0 (0%)	0 (0%)	-	-	0 (0%)	1 (100%)	3 (3%)
	Unknown/NA	-	1 (100%)	-	1 (7%)	0 (0%)	0 (0%)	0 (0%)	0 (0%)	1 (25%)	-	1 (6%)	0 (0%)	-	-	1 (14%)	0 (0%)	5 (5%)
	Total[#]	0 (0%)	1 (1%)	0 (0%)	15 (16%)	7 (8%)	1 (1%)	35 (38%)	1 (1%)	4 (4%)	0 (0%)	16 (18%)	3 (3%)	0 (0%)	0 (0%)	7 (8%)	1 (1%)	91
High response	Asymptomatic	0 (0%)	0 (0%)	-	5 (9%)	8 (36%)	0 (0%)	10 (30%)	1 (10%)	1 (11%)	1 (3%)	1 (2%)	0 (0%)	0 (0%)	0 (0%)	2 (22%)	-	29 (11%)
	Symptomatic	10 (83%)	0 (0%)	-	44 (79%)	12 (55%)	4 (100%)	20 (61%)	8 (80%)	8 (89%)	25 (83%)	43 (88%)	37 (97%)	1 (100%)	1 (50%)	5 (56%)	-	218 (79%)
	Hospitalised - No Oxygen Required	0 (0%)	0 (0%)	-	0 (0%)	1 (5%)	0 (0%)	1 (3%)	0 (0%)	0 (0%)	0 (0%)	1 (2%)	0 (0%)	0 (0%)	0 (0%)	0 (0%)	-	3 (1%)
	Hospitalised - Oxygen Required	0 (0%)	0 (0%)	-	0 (0%)	1 (5%)	0 (0%)	0 (0%)	1 (10%)	0 (0%)	1 (3%)	0 (0%)	0 (0%)	0 (0%)	0 (0%)	0 (0%)	-	3 (1%)
	ITU	0 (0%)	0 (0%)	-	0 (0%)	0 (0%)	0 (0%)	0 (0%)	0 (0%)	0 (0%)	0 (0%)	0 (0%)	0 (0%)	0 (0%)	0 (0%)	0 (0%)	-	0 (0%)
	COVID-19 related mortality	0 (0%)	0 (0%)	-	0 (0%)	0 (0%)	0 (0%)	1 (3%)	0 (0%)	0 (0%)	0 (0%)	0 (0%)	0 (0%)	0 (0%)	1 (50%)	0 (0%)	-	2 (1%)
	Unknown/NA	2 (17%)	1 (100%)	-	7 (13%)	0 (0%)	0 (0%)	1 (3%)	0 (0%)	0 (0%)	3 (10%)	4 (8%)	1 (3%)	0 (0%)	0 (0%)	2 (22%)	-	21 (8%)
	Total[#]	12 (4%)	1 (0%)	0 (0%)	56 (20%)	22 (8%)	4 (1%)	33 (12%)	10 (4%)	9 (3%)	30 (11%)	49 (18%)	38 (14%)	1 (0%)	2 (1%)	9 (3%)	0 (0%)	276
Total	Asymptomatic	0 (0%)	0 (0%)	0 (0%)	5 (7%)	10 (31%)	1 (14%)	20 (18%)	1 (8%)	1 (7%)	1 (3%)	1 (2%)	0 (0%)	0 (0%)	0 (0%)	3 (16%)	0 (0%)	43 (10%)
	Symptomatic	11 (85%)	1 (33%)	2 (67%)	57 (77%)	18 (56%)	4 (57%)	67 (59%)	10 (83%)	12 (86%)	25 (83%)	58 (89%)	40 (98%)	1 (100%)	1 (50%)	10 (53%)	0 (0%)	317 (74%)
	Hospitalised - No Oxygen Required	0 (0%)	0 (0%)	0 (0%)	1 (1%)	1 (3%)	0 (0%)	9 (8%)	0 (0%)	0 (0%)	0 (0%)	1 (2%)	0 (0%)	0 (0%)	0 (0%)	0 (0%)	1 (50%)	13 (3%)
	Hospitalised - Oxygen Required	0 (0%)	0 (0%)	0 (0%)	2 (3%)	2 (6%)	0 (0%)	9 (8%)	1 (8%)	0 (0%)	1 (3%)	0 (0%)	0 (0%)	0 (0%)	0 (0%)	0 (0%)	0 (0%)	15 (3%)
	ITU	0 (0%)	0 (0%)	0 (0%)	1 (1%)	0 (0%)	0 (0%)	0 (0%)	0 (0%)	0 (0%)	0 (0%)	0 (0%)	0 (0%)	0 (0%)	0 (0%)	1 (5%)	0 (0%)	2 (0%)
	COVID-19 related mortality	0 (0%)	0 (0%)	1 (33%)	0 (0%)	1 (3%)	2 (29%)	4 (4%)	0 (0%)	0 (0%)	0 (0%)	0 (0%)	0 (0%)	0 (0%)	1 (50%)	0 (0%)	1 (50%)	10 (2%)
	Unknown/NA	2 (15%)	2 (67%)	0 (0%)	8 (11%)	0 (0%)	0 (0%)	4 (4%)	0 (0%)	1 (7%)	3 (10%)	5 (8%)	1 (2%)	0 (0%)	0 (0%)	5 (26%)	0 (0%)	31 (7%)
	Total[#]	13 (3%)	3 (1%)	3 (1%)	74 (17%)	32 (7%)	7 (2%)	113 (26%)	12 (3%)	14 (3%)	30 (7%)	65 (15%)	41 (10%)	1 (0%)	2 (0%)	19 (4%)	2 (0%)	431

Supplementary Table 15. Summary of COVID-19 severity reported in combined follow-up following COVID-19 vaccination for all immunocompromised patients in OCTAVE that were COVID-19 naïve prior to vaccination, split by disease subgroup and based on Roche anti-RBD Ig assay result ≥0.8AU/mL, low response <380AU/mL and high response >380AU/mL at the post-V2 timepoint.
[#] % of total OCTAVE infections

		COVID-19 naïve at baseline, T cell breakdown - <6months post-v2 - 12months post-V1 (n (%))																
		**SC**	**HM**	**AAV**	**IA**	**HD**	**HD on IS**	**K-Tr ***	**L-Tr**	**L-AI**	**L-Cirr**	**CD**	**UC**	**IBD-U***	**Auto-HSCT**	**Allo-HSCT**	**CAR-T**	**OCTAVE**
**No response**	**Asymptomatic**	-	-	-	0 (0%)	2 (29%)	1 (50%)	-	0 (0%)	-	-	-	0 (0%)	-	-	1 (33%)	0 (0%)	4 (24%)
	**Symptomatic**	-	-	-	2 (100%)	3 (43%)	1 (50%)	-	1 (100%)	-	-	-	1 (100%)	-	-	1 (33%)	0 (0%)	9 (53%)
	**Hospitalised - No Oxygen Required**	-	-	-	0 (0%)	1 (14%)	0 (0%)	-	0 (0%)	-	-	-	0 (0%)	-	-	0 (0%)	0 (0%)	1 (6%)
	**Hospitalised - Oxygen Required**	-	-	-	0 (0%)	0 (0%)	0 (0%)	-	0 (0%)	-	-	-	0 (0%)	-	-	0 (0%)	0 (0%)	0 (0%)
	**ITU**	-	-	-	0 (0%)	0 (0%)	0 (0%)	-	0 (0%)	-	-	-	0 (0%)	-	-	1 (33%)	0 (0%)	1 (6%)
	**COVID-19 related mortality**	-	-	-	0 (0%)	1 (14%)	0 (0%)	-	0 (0%)	-	-	-	0 (0%)	-	-	0 (0%)	1 (100%)	2 (12%)
	**Unknown/NA**	-	-	-	0 (0%)	0 (0%)	0 (0%)	-	0 (0%)	-	-	-	0 (0%)	-	-	0 (0%)	0 (0%)	0 (0%)
	**Total#**	0 (0%)	0 (0%)	0 (0%)	2 (12%)	7 (41%)	2 (12%)	-	1 (6%)	0 (0%)	0 (0%)	0 (0%)	1 (6%)	-	0 (0%)	3 (18%)	1 (6%)	17
**Yes response**	**Asymptomatic**	0 (0%)	0 (0%)	0 (0%)	4 (22%)	3 (38%)	0 (0%)	-	0 (0%)	1 (20%)	1 (14%)	0 (0%)	0 (0%)	-	0 (0%)	1 (50%)	-	10 (12%)
	**Symptomatic**	6 (86%)	0 (0%)	2 (67%)	7 (39%)	4 (50%)	3 (100%)	-	1 (50%)	4 (80%)	2 (29%)	11 (100%)	16 (100%)	-	0 (0%)	1 (50%)	-	57 (67%)
	**Hospitalised - No Oxygen Required**	0 (0%)	0 (0%)	0 (0%)	1 (6%)	0 (0%)	0 (0%)	-	0 (0%)	0 (0%)	0 (0%)	0 (0%)	0 (0%)	-	0 (0%)	0 (0%)	-	1 (1%)
	**Hospitalised - Oxygen Required**	0 (0%)	0 (0%)	0 (0%)	1 (6%)	1 (13%)	0 (0%)	-	1 (50%)	0 (0%)	1 (14%)	0 (0%)	0 (0%)	-	0 (0%)	0 (0%)	-	4 (5%)
	**ITU**	0 (0%)	0 (0%)	0 (0%)	0 (0%)	0 (0%)	0 (0%)	-	0 (0%)	0 (0%)	0 (0%)	0 (0%)	0 (0%)	-	0 (0%)	0 (0%)	-	0 (0%)
	**COVID-19 related mortality**	0 (0%)	0 (0%)	1 (33%)	0 (0%)	0 (0%)	0 (0%)	-	0 (0%)	0 (0%)	0 (0%)	0 (0%)	0 (0%)	-	1 (100%)	0 (0%)	-	2 (2%)
	**Unknown/NA**	1 (14%)	2 (100%)	0 (0%)	5 (28%)	0 (0%)	0 (0%)	-	0 (0%)	0 (0%)	3 (43%)	0 (0%)	0 (0%)	-	0 (0%)	0 (0%)	-	11 (13%)
	**Total#**	7 (8%)	2 (2%)	3 (4%)	18 (21%)	8 (9%)	3 (4%)	-	2 (2%)	5 (6%)	7 (8%)	11 (13%)	16 (19%)	-	1 (1%)	2 (2%)	0 (0%)	85
**Total**	**Asymptomatic**	0 (0%)	0 (0%)	0 (0%)	4 (20%)	5 (33%)	1 (20%)	-	0 (0%)	1 (20%)	1 (14%)	0 (0%)	0 (0%)	-	0 (0%)	2 (40%)	0 (0%)	14 (14%)
	**Symptomatic**	6 (86%)	0 (0%)	2 (67%)	9 (45%)	7 (47%)	4 (80%)	-	2 (67%)	4 (80%)	2 (29%)	11 (100%)	17 (100%)	-	0 (0%)	2 (40%)	0 (0%)	66 (65%)
	**Hospitalised - No Oxygen Required**	0 (0%)	0 (0%)	0 (0%)	1 (5%)	1 (7%)	0 (0%)	-	0 (0%)	0 (0%)	0 (0%)	0 (0%)	0 (0%)	-	0 (0%)	0 (0%)	0 (0%)	2 (2%)
	**Hospitalised - Oxygen Required**	0 (0%)	0 (0%)	0 (0%)	1 (5%)	1 (7%)	0 (0%)	-	1 (33%)	0 (0%)	1 (14%)	0 (0%)	0 (0%)	-	0 (0%)	0 (0%)	0 (0%)	4 (4%)
	**ITU**	0 (0%)	0 (0%)	0 (0%)	0 (0%)	0 (0%)	0 (0%)	-	0 (0%)	0 (0%)	0 (0%)	0 (0%)	0 (0%)	-	0 (0%)	1 (20%)	0 (0%)	1 (1%)
	**COVID-19 related mortality**	0 (0%)	0 (0%)	1 (33%)	0 (0%)	1 (7%)	0 (0%)	-	0 (0%)	0 (0%)	0 (0%)	0 (0%)	0 (0%)	-	1 (100%)	0 (0%)	1 (100%)	4 (4%)
	**Unknown/NA**	1 (14%)	2 (100%)	0 (0%)	5 (25%)	0 (0%)	0 (0%)	-	0 (0%)	0 (0%)	3 (43%)	0 (0%)	0 (0%)	-	0 (0%)	0 (0%)	0 (0%)	11 (11%)
	**Total#**	7 (7%)	2 (2%)	3 (3%)	20 (20%)	15 (15%)	5 (5%)	-	3 (3%)	5 (5%)	7 (7%)	11 (11%)	17 (17%)	-	1 (1%)	5 (5%)	1 (1%)	102

Supplementary Table 16. Summary of COVID-19 severity following vaccination in group1 participants of the OCTAVE cohort which were SARS-CoV-2 infection naïve prior to vaccination, split by disease subgroup and based on IFNy ELISpot response to SARS-CoV-2 spike at the post-V2 timepoint. No response = 0SFC/10^6 , Yes response >0SFC/10^6. * K-Tr and IBD-U groups were not included in group 1. # % of total OCTAVE infections

Reaction	Grade	Astrazeneca (n=1,735)	Pfizer (n=912)	Moderna (n=3)	Unknown (n=12)	Total (n=2,662)
Injection Site Pain	1	395 (23%)	124 (14%)	1 (33%)	0 (0%)	520 (20%)
	2	83 (5%)	19 (2%)	0 (0%)	0 (0%)	102 (4%)
	3	4 (0%)	1 (0%)	0 (0%)	0 (0%)	5 (0%)
	Unknown	53 (3%)	17 (2%)	0 (0%)	0 (0%)	70 (3%)
Injection Site Redness	1	66 (4%)	13 (1%)	1 (33%)	0 (0%)	80 (3%)
	Not applicable	1 (0%)	0 (0%)	0 (0%)	0 (0%)	1 (0%)
	Unknown	8 (0%)	1 (0%)	0 (0%)	0 (0%)	9 (0%)
Injection Site Swelling	1	53 (3%)	9 (1%)	1 (33%)	0 (0%)	63 (2%)
	2	11 (1%)	2 (0%)	0 (0%)	0 (0%)	13 (0%)
	Unknown	5 (0%)	0 (0%)	0 (0%)	0 (0%)	5 (0%)
Anaphylaxis	Unknown	1 (0%)	0 (0%)	0 (0%)	0 (0%)	1 (0%)
Arthralgia	1	98 (6%)	9 (1%)	0 (0%)	0 (0%)	107 (4%)
	2	65 (4%)	5 (1%)	0 (0%)	0 (0%)	70 (3%)
	3	3 (0%)	1 (0%)	0 (0%)	0 (0%)	4 (0%)
	Unknown	12 (1%)	3 (0%)	0 (0%)	0 (0%)	15 (1%)
Chills	1	146 (8%)	12 (1%)	0 (0%)	0 (0%)	158 (6%)
	2	102 (6%)	4 (0%)	0 (0%)	0 (0%)	106 (4%)
	3	2 (0%)	0 (0%)	0 (0%)	0 (0%)	2 (0%)
	Unknown	22 (1%)	1 (0%)	0 (0%)	0 (0%)	23 (1%)
Headache	1	218 (13%)	33 (4%)	0 (0%)	0 (0%)	251 (9%)
	2	138 (8%)	14 (2%)	0 (0%)	0 (0%)	152 (6%)
	3	10 (1%)	1 (0%)	0 (0%)	0 (0%)	11 (0%)
	Unknown	23 (1%)	3 (0%)	0 (0%)	0 (0%)	26 (1%)
Myalgia	1	144 (8%)	18 (2%)	0 (0%)	0 (0%)	162 (6%)
	2	79 (5%)	7 (1%)	0 (0%)	0 (0%)	86 (3%)
	3	2 (0%)	1 (0%)	0 (0%)	0 (0%)	3 (0%)
	Unknown	23 (1%)	4 (0%)	0 (0%)	0 (0%)	27 (1%)
Nausea	1	83 (5%)	15 (2%)	0 (0%)	0 (0%)	98 (4%)
	2	27 (2%)	2 (0%)	0 (0%)	0 (0%)	29 (1%)
	3	1 (0%)	0 (0%)	0 (0%)	0 (0%)	1 (0%)
	Unknown	8 (0%)	2 (0%)	0 (0%)	0 (0%)	10 (0%)
Pyrexia/Fever	1	128 (7%)	15 (2%)	0 (0%)	0 (0%)	143 (5%)
	2	109 (6%)	3 (0%)	0 (0%)	0 (0%)	112 (4%)
	3	2 (0%)	0 (0%)	0 (0%)	0 (0%)	2 (0%)
	Unknown	23 (1%)	2 (0%)	0 (0%)	0 (0%)	25 (1%)

Supplementary Table 17: Summary of adverse reactions following first vaccination for immunocompromised patients in OCTAVE (n = 2662), split by vaccine type.

Reaction	Grade	Astrazeneca (n=1,713)	Pfizer (n=902)	Moderna (n=6)	Unknown (n=8)	Total (n=2,629)
Injection Site Pain	1	305 (18%)	123 (14%)	2 (33%)	0 (0%)	430 (16%)
	2	49 (3%)	20 (2%)	1 (17%)	0 (0%)	70 (3%)
	3	2 (0%)	2 (0%)	0 (0%)	0 (0%)	4 (0%)
	Unknown	40 (2%)	11 (1%)	0 (0%)	0 (0%)	51 (2%)
Injection Site Redness	1	40 (2%)	16 (2%)	0 (0%)	0 (0%)	56 (2%)
	Unknown	5 (0%)	3 (0%)	0 (0%)	0 (0%)	8 (0%)
Injection Site Swelling	1	36 (2%)	10 (1%)	0 (0%)	0 (0%)	46 (2%)
	2	8 (0%)	1 (0%)	0 (0%)	0 (0%)	9 (0%)
	3	0 (0%)	1 (0%)	0 (0%)	0 (0%)	1 (0%)
	Unknown	2 (0%)	0 (0%)	0 (0%)	0 (0%)	2 (0%)
Anaphylaxis	Unknown	1 (0%)	0 (0%)	0 (0%)	0 (0%)	1 (0%)
Arthralgia	1	48 (3%)	11 (1%)	0 (0%)	0 (0%)	59 (2%)
	2	24 (1%)	4 (0%)	0 (0%)	0 (0%)	28 (1%)
	3	3 (0%)	2 (0%)	0 (0%)	0 (0%)	5 (0%)
	Unknown	5 (0%)	1 (0%)	0 (0%)	0 (0%)	6 (0%)
Chills	1	65 (4%)	11 (1%)	0 (0%)	0 (0%)	76 (3%)
	2	27 (2%)	5 (1%)	0 (0%)	0 (0%)	32 (1%)
	3	2 (0%)	1 (0%)	0 (0%)	0 (0%)	3 (0%)
	Unknown	9 (1%)	2 (0%)	0 (0%)	0 (0%)	11 (0%)
Headache	1	133 (8%)	31 (3%)	0 (0%)	0 (0%)	164 (6%)
	2	57 (3%)	11 (1%)	0 (0%)	0 (0%)	68 (3%)
	3	7 (0%)	3 (0%)	0 (0%)	0 (0%)	10 (0%)
	Unknown	13 (1%)	2 (0%)	0 (0%)	0 (0%)	15 (1%)
Myalgia	1	72 (4%)	15 (2%)	0 (0%)	0 (0%)	87 (3%)
	2	35 (2%)	5 (1%)	0 (0%)	0 (0%)	40 (2%)
	3	4 (0%)	2 (0%)	0 (0%)	0 (0%)	6 (0%)
	Unknown	8 (0%)	3 (0%)	0 (0%)	0 (0%)	11 (0%)
Nausea	1	41 (2%)	6 (1%)	0 (0%)	0 (0%)	47 (2%)
	2	10 (1%)	3 (0%)	0 (0%)	0 (0%)	13 (0%)
	3	1 (0%)	0 (0%)	0 (0%)	0 (0%)	1 (0%)
	Unknown	2 (0%)	2 (0%)	0 (0%)	0 (0%)	4 (0%)
Pyrexia/Fever	1	58 (3%)	14 (2%)	0 (0%)	0 (0%)	72 (3%)
	2	28 (2%)	8 (1%)	0 (0%)	0 (0%)	36 (1%)
	3	3 (0%)	1 (0%)	0 (0%)	0 (0%)	4 (0%)
	Unknown	10 (1%)	4 (0%)	0 (0%)	0 (0%)	14 (1%)

Supplementary Table 18: Summary of adverse reactions following second vaccination for immunocompromised patients in OCTAVE (n = 2629), split by vaccine type.

Reason	Admitting Event	Grade	Other Events	Relatedness	Classification	Outcome
Hospitalisation	Myalgia	1	Headache; Fatigue; Chest Discomfort	Probably related	SAR	Resolved - no sequelae
Hospitalisation	Cough	2	Dyspnea	Possibly related	SAR	Resolved - no sequelae
Hospitalisation	Thrombocytopenia	4	Headache; Neutrophil count decreased	Possibly related	Non fatal/life-threatening SUSAR	Resolved - with sequelae

Supplementary Table 19: Summary of serious adverse events following one or two doses of COVID-19 vaccine in immunocompromised patients in OCTAVE (n = 2629).

SUSAR Description	
SUSAR Type	Thrombocytopenia*
Vaccine type	ChAdOx1 nCoV-19
Disease subgroup	Allo-HSCT
SUSAR time from vaccine	4-weeks
Nadir platelet count pre-vaccine	140 x 10*9 /L
Nadir platelet count post-vaccine	15 x 10*9/L
Outcome:	No thrombosis/vaccine-induced prothrombotic thrombocytopenia, recovered without sequelae

* at the time of reporting, thrombocytopenia was not a listed adverse reaction of ChAdOx1 nCoV-19 on the Reg 174 Information For UK Healthcare Professionals, but has since been added.

Supplementary Table 20: Further description of SUSAR event.

OCTAVE Adult Participants Recruitment by Site

Site Name	Principal Investigator	Date Opened to Recruitment	Participant Recruitment		
			Serology Group	Deep Immunophenotyping Group	Total
**Addenbrooke's Hospital, Cambridge**	Dr, R, Malladi	14/05/2021	10	0	10
**Freeman Hospital, Newcastle upon Tyne**	Dr, E, Hurst	06/07/2021	24	0	24
**Glasgow Royal Infirmary, Glasgow**	Prof, S, Siebert	17/02/2021	536	150	686
**Hammersmith Hospital, London**	Dr, M, Willicombe	10/03/2021	882	152	1034
**John Radcliffe Hospital, Oxford**	Prof, E, Barnes	26/02/2021	347	197	544
**King's College Hospital, London**	Dr, R, Sanderson	19/05/2021	1	8	9
**Royal Hallamshire Hospital, Sheffield**	Prof, J, Snowden	25/03/2021	35	33	68
**Southampton General Hospital, Southampton**	Dr, K, Orchard	18/03/2021	11	20	31
**St George's Hospital, London**	Dr, M, Koh	05/07/2021	14	0	14
**St James's University Hospital, Leeds**	Prof, G, Cook	26/03/2021	10	14	24
**Queen Elizabeth Hospital, Birmingham**	Prof, DW, Rea	15/03/2021	140	102	242
**Total**			2010	676	2686

Supplementary Table 21: Recruitment of participants to OCTAVE.

	Cov-1S	Cov-2 NTD	Cov-2 RBD	Cov-2N	Cov-2S
**IgG**	999.575	32.55316	1865.637	2957.245	1120.589
**IgA**	382.1977	565.7463	999.2172	1358.686	505.3715
**IgM**	234.4206	53.94288	977.4923	4958.536	344.734

Supplementary Table 22: Cut-off for seropositivity on MSD assay defined from pre-pandemic sera in healthy controls.

Supplementary material appendix 1
OCTAVE trial protocol version 9.0

Observational Cohort Trial-T-cells Antibodies and Vaccine Efficacy in SARS-CoV-2

Protocol

Version: 9.0, 15th March 2022

Sponsor: University of Birmingham

**EudraCT Number:**	2021-000569-33
**Sponsor Number:**	RG_21-007
**CRCTU Protocol Number:**	MX1034
**IRAS Number:**	294480
**ISRCTN Number:**	12821688

TRIAL CONTACTS

**Sponsor:**	University of Birmingham, Edgbaston, Birmingham. B15 2TT
**Chief Investigator:**	Professor Iain McInnes, University of Glasgow
**Cohort Leads/Co-leads:**	
**Adult Rheumatoid/Inflammatory Diseases**	Professor Iain McInnes and Professor Stefan Siebert, University of Glasgow<br>☏ 0141 330 4876/0141 330 3375
**Adult Cancer (Solid Tumours)**	Professor Pam Kearns and Professor Daniel Rea, University of Birmingham<br>☏ 0121 414 7854/0121 414 5345
**Adult Cancer (Haematology)**	Professor Gordon Cook, University of Leeds<br>☏ 0113 343 9038
**Adult Chronic Renal Disease**	Dr Michelle Willicombe and Dr David Thomas, Imperial College London<br>☏ 020 3313 8506
**Adult Chronic Liver Disease**	Professor Eleanor Barnes, Oxford University<br>☏ 01865 281 547
**Adult Stem Cell Transplant**	Dr Paul Miller, St George's University Hospitals NHS Foundation Trust and<br>Dr Thushan de Silva, University of Sheffield<br>☏ 0114 215 9522
**Paediatric Rheumatology & Inflammatory Diseases Lead**	Professor Lucy Wedderburn and Dr Elena Moraitis<br>Great Ormond Street Hospital<br>☏ 0207 405 9200 ext. 7887
**Paediatric Solid Organ Transplantation Lead**	Dr Rossa Brugha<br>Great Ormond Street Hospital<br>☏ 0207 405 9200 ext. 5643
**Paediatric Cancer Lead**	Dr Jessica Bate<br>Southampton Children's Hospital<br>☏ 02381 206 334
**Laboratory Lead**	Professor Carl Goodyear, University of Glasgow<br>☏ 0141 330 3865
**Other Co-Investigators:**	Professor Doreen Cantrell, University of Dundee<br>Professor Ronjon Chakraverty, University College London<br>Professor Paul Klenerman, University of Oxford<br>Dr Jack Satsangi, Oxford University Hospitals<br>Professor Gary Middleton, University of Birmingham<br>Professor Paul Moss, University of Birmingham<br>Professor Duncan Porter, University of Glasgow<br>Professor Alex Richter, University of Birmingham<br>Professor John Snowden, University of Sheffield<br>Dr Neil Basu, University of Glasgow<br>Dr Kim Orchard, University of Southampton<br>Miss Amanda Kirkham, University of Birmingham
**OCTAVE Trial Office:**	Cancer Research UK Clinical Trials Unit (CRCTU), University of Birmingham, Edgbaston, Birmingham, B15 2TT

Sponsor:	University of Birmingham, Edgbaston, Birmingham. B15 2TT
Email	✉ OCTAVE@trials.bham.ac.uk
Website	www.birmingham.ac.uk/octave
Trial Office Contacts	
Trial Statistician	Miss Amanda Kirkham and Charlotte Gaskell
CRCTU Director of Operations	Dr Sarah Bowden
Trial Management Team Leader	Ms Sophia Magwaro
Senior Manager	Mrs Ana Hughes
Trial Coordinators	Mrs Molly Harrison and Dr Ann Pope

SIGNATURE PAGE

OCTAVE Trial Protocol

This protocol has been approved by:

**Name:**	IAIN MUNNGY	**Trial Role:**	Chief Investigator
**Signature:**	[signature]	Date:	15/MAR/2022

This protocol describes the OCTAVE trial and provides information about procedures for participants taking part in the trial.

SPONSOR STATEMENT

Where the University of Birmingham takes on the sponsor role for protocol development oversight, the signing of the IRAS form by the sponsor will serve as confirmation of approval of this protocol.

AMENDMENTS

The following amendments and/or administrative changes have been made to this protocol since the implementation of the first approved version

Amendment number	Date of amendment	Protocol version number	Type of amendment	Summary of amendment
SAM01	24-Feb-2021	V 2.0	Substantial	Change to eligibility criteria to allow the Deep Immunophenotyping Group participants to enter the trial after receiving the first vaccination and Serology Group prior to post-booster time point.<br>Clarification of sample collection requirement.<br>Other non-substantial changes including addition of Trial Summary
SA_04	12-Apr-2021	V 3.0	Substantial	Removed reference to the Green Book and added that vaccines should be administered in accordance with national guidelines and current versions of the applicable information for healthcare professionals.
NSA_01	22-Apr-2021	V 4.0	Non-Substantial	Change in eligibility criteria to add recruitment of participants with systemic lupus erythematosus (SLE) and treatment with IL-12/23 inhibitors and IL-23 inhibitors<br>Clarification of sample collection time points and Other non-substantial changes, including: change of term boost to booster to refer to second dose of the vaccine; changes to CRF Forms table; inclusion on on-site monitoring section.
SA_05	24-Jun-2021	V 5.0	Substantial	Clarification of sample collection time points<br>Change of sample size<br>Clarification eligibility criteria for HSCT patients
NSA_04	14-Jul-2021	V 6.0	Non-Substantial	Addition of provision to Principal Investigators with the results of their patients' assessment of immune response and that participants should be provided with the results on request.

Amendment number	Date of amendment	Protocol version number	Type of amendment	Summary of amendment
NSA_05	9-Sep-2021	V7.0	Non-Substantial	Clarification that the 6 month sample time-point should be as close as possible to 6 months or prior to 3rd vaccine dose, as applicable; the 12 month sample time point be as close as possible to 12 months, and for saliva samples to be taken where possible at all time points.
SA_07	21-Dec-2021	V8.0	Substantial	Addition of Serology Plus Group for the adolescent population. Changes made throughout the protocol to accommodate this addition.
SA_08	15-Mar-2022	V9.0	Substantial	Expansion of the Serology Plus Group to include 5 to 11 year olds.<br>Clarification of statistical analyses methods.<br>Other minor administrative changes including update of Public Health England Laboratories name.

TRIAL SYNOPSIS

**Title**	**OCTAVE**: Observational Cohort Trial -T-cells Antibodies and Vaccine Efficacy in SARS-CoV-2
**Trial Design**	Multi-centre, multi-disease, prospective observational cohort trial of the immune response to SARS-CoV-2 vaccination.
**Aim**	To evaluate the immune response to SARS-CoV-2 vaccination in clinically vulnerable groups across the UK.
**Objectives**	**Primary Objective**<br>• To determine the magnitude of the humoral and T cell immunogenicity of SARS-CoV-2 vaccines in participants with chronic diseases and/or secondary immunodeficiency.<br><br>**Secondary Objectives**<br>• To determine phenotype and function of SARS-CoV-2 vaccine induced immune responses in participants with chronic diseases and/or secondary immunodeficiency, compared to each other and healthy controls in parallel studies.<br>• To evaluate the impact of distinct immune therapeutic drug classes on the development of humoral and cellular immune responses to SARS-CoV-2 following vaccination.
**Outcome Measures**	**Primary Outcomes**<br>Vaccine Specific Immunogenicity:<br>• To measure the presence and amount of serum antibodies to discriminate IgG responses to SARS-CoV-2 from vaccination and/or infection.<br>• To measure T cell responses to SARS-CoV-2 peptides following vaccination.<br><br>**Secondary Outcomes**<br>Clinical Protection<br>• The first symptomatic PCR-proven COVID-19 occurrence from 14 days after first dose of vaccine in participants without evidence of prior infection with SARS-CoV-2.<br><br>**Exploratory Outcomes**<br>Humoral Immunogenicity<br>• To assess the capacity of vaccine induced SARS-CoV-2 antibodies to neutralise/block SARS-CoV-2 infection.<br><br>Cellular Immunogenicity<br>• To assess the relative contribution of T cell subsets and T cell function and the recall potential of SARS-CoV-2 memory T cells at later time points.
**Patient Population**	Adult participants with end stage kidney disease, liver disease or gastrointestinal disease on immune suppressive therapy, cancer, immune-mediated rheumatic diseases and haematopoietic stem cell transplant recipients who are receiving the SARS-CoV-2 as part of the national vaccination programme.

	○ Children and Adolescent cohort between the ages of 5 and <18 years (17 years 364 days inclusive) with immune mediated diseases, post solid organ transplantation or with a cancer diagnosis who are receiving a SARS-CoV-2 vaccine as part of the national vaccination programme.
**Sample Size**	**Deep Immunophenotyping Group:** between 100 and 200 adult participants depending on disease cohort will be recruited for full immune response analysis.<br>**Serology Group:** between 150 and 850 adult participants depending on disease cohort, for serology analysis<br>**Serology Plus Group**: up to 160 children and adolescent participants; provisional sub-group numbers are 60 rheumatology patients, 50 post solid organ transplantation, and 50 with childhood cancer<br>**Total:** Up to 3250 participants
**Inclusion Criteria**	• Eligible for vaccination by one of the SARS-CoV-2 approved vaccines and:<br>○ For the Deep Immunophenotyping Group, have not received the second dose of vaccine (booster)<br>○ For the Serology Group, have not passed 28 days post second vaccine dose (booster) within -7/+56 days)<br>○ For the Serology Plus Group, either have not passed 28 days post-second vaccine dose (booster) (within -7/+56 days) or up to 6 months post second vaccine dose, including patients who have received a third or further dose in that time period<br>○ Anticipated life expectancy of ≥ 6 months<br>• Fall into one (or more) of the disease cohorts who will meet disease relevant classification, disease state, and staging according to established international standards (refer to protocol for details)
**Sample Collection**	**Deep Immunophenotyping Group:** whole blood, peripheral blood mononuclear cells (PBMC), serum, plasma and saliva will be collected at the following time points:<br>• Pre-vaccine, baseline (optional) – may have been collected prior to recruitment to OCTAVE<br>• 1 day after first vaccination (optional) – tempus and EDTA sample only<br>• Pre-second vaccine dose (booster)<br>• 28 days post-second vaccine dose (ideally within +/- 3 days)<br>• 6 months post-second vaccine dose (as close to time point as possible)<br>• 12 months after first vaccine dose or prior to third vaccine dose (if applicable), whichever is earlier<br><br>**Serology Group:** serum (as a minimum) collected at the following time points:<br>• Pre-vaccine, baseline (optional) – may have been collected prior to recruitment to OCTAVE<br>• Pre-second vaccine dose (booster) (optional) – any time after first vaccination and before second dose<br>• 28 days post-second dose (within -7/+ 56 days)<br><br>**Serology Plus Group:**<br>• A sample will be collected prior to a vaccine dose being given at trial entry (referred to as baseline (pre-first dose), pre-second vaccine, pre-third vaccine or pre-fourth vaccine, as applicable)

	• A sample will be obtained 28 days (-7/+56 days) post vaccination (referred to as post-first dose, post-second dose, post-third dose or post-fourth dose, as applicable)<br>• Further samples can be obtained 28 days (-7/+56 days) post subsequent vaccination doses (optional), referred to as post-second dose, post-third dose and/or fourth dose, as applicable<br><br>Note: Participants can enter prior to their first, second, third or fourth vaccine doses, and will be asked to provide a blood sample at no more than four time-points.
**Trial Duration**	Adult cohort patients will be recruited over a six month period and followed-up for 12 months in accordance with standard clinical practice for the relevant disease cohort.<br><br>Children and Adolescent cohort participants will be recruited over a six month period with six month follow-up where possible, in accordance with standard clinical practice for the relevant disease cohort.
**Contacts**	**Sponsor:** University of Birmingham<br>**Chief Investigator:** Professor Iain McInnes, University of Glasgow<br>**OCTAVE Trial Office:** Cancer Research UK Clinical Trials Unit (CRCTU), University of Birmingham, Edgbaston, Birmingham, B15 2TT ✉ OCTAVE@trials.bham.ac.uk

SCHEDULE OF EVENTS

		Screening	Trial Entry	Baseline[1] *Prior to vaccination*	Day 1 After First Vaccination[2]	Pre-second vaccine dose [3] *(Booster)*	Post-second vaccine dose[4] *(Booster)* *28 days post vaccination*	Pre-additional vaccine dose[5]	Post-additional vaccine dose[6] *28 days post vaccination*	6 Months Post-second Vaccination[7]	6 Month Follow-up[8] *Seen in accordance with clinical practice*	12 Months[9]
Eligibility assessment		x										
Consent		x										
Trial entry			x									
Research samples	Deep immunophenotyping Group[10]			x	x	x	x			x		x
	Serology Group[11]			x		x	x					
	Serology Plus Group[12]			x		x	x	x	x			
Data collection				x			x				x	x

Key

[1] All Groups. Optional sample. Baseline research samples may also be collected prior to recruitment to OCTAVE as part of another Research Ethics Committee (REC) approved study
[2] Deep Immunophenotyping Group only. Optional sample. To include 3ml tempus sample and 2-3 x 9ml EDTA samples only
[3] Mandatory for Deep Immunophenotyping Group, optional sample for Serology Group and Serology Plus Group; taken any time after first vaccination but before second vaccination (booster)
[4] Mandatory for Deep Immunophenotyping Group and Serology Group, optional sample for Serology Plus Group; taken within +/- 3 days for the Deep Immunophenotyping Group and -7/+ 56 days for the Serology and Serology Plus Groups
[5] Serology Plus only. Mandatory sample but only required when the participant's trial entry is post second or subsequent vaccine doses
[6] Serology Plus only. Optional sample. Only where the participant's trial entry is post second vaccine or subsequent doses; taken within the range -7/+ 56 days
[7] Deep Immunophenotyping Group only. Six months after second vaccine dose, as close to time point as possible or prior third vaccine dose (if applicable), whichever is earlier
[8] All Groups, 6 months after last trial vaccination, data collected retrospectively from participants medical records
[9] Deep Immunophenotyping Group and Serology Group only, 12 months after first vaccine dose
[10] Research samples include: whole blood, peripheral blood mononuclear cells (PBMC), serum, plasma and saliva (where possible)
[11] Research samples include: serum as a minimum, whole blood, peripheral blood mononuclear cells (PBMC), and plasma where possible
[12] Research samples include: whole blood and serum. Up to four sample time-points

CRCTU-PRT-QCD-001, version 1.0

TABLE OF CONTENTS

1. BACKGROUND AND RATIONALE ... 1
1.1 SARS-CoV-2 Vaccines ... 1
1.2 Justification for the Trial and Adult Participant Populations ... 1
1.2.1 Cancer ... 1
1.2.2 Rheumatic and Inflammatory Diseases ... 5
1.2.3 Chronic Renal Diseases ... 5
1.2.4 Chronic Liver Diseases or with Gastrointestinal Disease on Immune Suppressive Therapy ... 6
1.2.5 Haematopoietic Stem Cell Transplant Recipients ... 6
1.3 Justification for the Trial and Children and Adolescent Participant Populations ... 7
1.3.1 Solid Organ Transplant ... 7
1.3.2 Immune-mediated Rheumatic Diseases ... 7
1.3.3 Childhood Cancer ... 8
2. AIM, OBJECTIVES AND OUTCOME MEASURES ... 8
2.1 Objectives ... 8
2.1.1 Primary Objective ... 8
2.1.2 Secondary Objectives ... 8
2.2 Outcome Measures ... 9
2.2.1 Primary Outcomes ... 9
2.2.2 Secondary Outcome ... 9
2.2.3 Exploratory Outcomes ... 9
3. TRIAL DESIGN ... 10
4. ELIGIBILITY ... 10
4.1 Inclusion Criteria ... 11
5. SCREENING AND CONSENT ... 13
5.1 Screening ... 13
5.2 Informed Consent ... 13
6. TRIAL ENTRY ... 15
7. TRIAL REQUIREMENTS ... 15
7.1 Investigational Medicinal Products ... 15
7.1.1 Pharmacy Requirements ... 16
7.2 Vaccination ... 16
7.2.1 First Vaccination ... 16
7.2.2 Second Vaccination (Booster) and Subsequent Vaccine Doses ... 17
7.3 Assessments ... 17
7.4 Sample Collection and Analysis ... 17
7.4.1 Collection ... 17
7.4.2 Analysis ... 20
7.4.3 Future Research and Biobanking ... 21
7.5 Participant Follow-Up ... 21
7.6 Data Linkage ... 21
7.7 Participant Withdrawal ... 21
8. ADVERSE EVENT REPORTING ... 22
8.1 Reporting Requirements ... 22
8.1.1 Adverse Events ... 22
8.1.2 Serious Adverse Events ... 22
8.2 Reporting Period ... 22

8.3 Reporting Procedure **22**
8.3.1 Site 22
8.3.2 OCTAVE Trial Office 23
8.3.3 Reporting to the Competent Authority and Research Ethics Committee 24
9. DATA COLLECTION **24**
9.1 Archiving **27**
10. QUALITY MANAGEMENT **27**
10.1 Site Set-up and Initiation **27**
10.2 On-site Monitoring **27**
10.3 Central Monitoring **27**
10.4 Audit and Inspection **28**
10.5 Notification of Serious Breaches **28**
11. END OF TRIAL DEFINITION **28**
12. STATISTICAL CONSIDERATIONS **28**
12.1 Definition of Outcome Measures **28**
12.1.1 Primary Outcome Measures 28
12.1.2 Secondary Outcome Measures 28
12.2 Analysis of Outcome Measures **29**
12.2.1 Primary Outcome Measures 29
12.2.2 Secondary Outcome Measures 30
12.2.3 Planned Subgroup Analyses 30
12.2.4 Exploratory Analyses 30
12.2.5 Sample Size Justification 30
13. TRIAL ORGANISATIONAL STRUCTURE **30**
13.1 Sponsor **30**
13.2 Coordinating Centre **31**
13.3 Access Committee **31**
13.4 Trial Management Group **31**
13.5 Independent Oversight Committees **31**
13.6 Finance **31**
14. ETHICAL CONSIDERATIONS **32**
15. CONFIDENTIALITY AND DATA PROTECTION **32**
16. INSURANCE AND INDEMNITY **33**
17. PUBLICATION POLICY **33**
18. DATA SHARING **34**
19. REFERENCE LIST **35**
APPENDIX 1 - DEFINITION OF ADVERSE EVENTS **38**
1.1 Adverse Event (AE) **38**
1.2 Adverse Reaction (AR) **38**
1.3 Serious Adverse Event (SAE) **38**
1.4 Serious Adverse Reaction (SAR) **39**
1.5 Suspected Unexpected Serious Adverse Reaction (SUSAR) **39**
1.6 Unexpected Adverse Reaction (UAR) **39**
APPENDIX 2 - COMMON TOXICITY CRITERIA GRADINGS **40**
APPENDIX 3 – WORLD HEALTH ORGANISATION (WHO) PERFORMANCE STATUS **41**
APPENDIX 4 - WMA DECLARATION OF HELSINKI **42**

CRCTU-PRT-QCD-001, version 1.0

1. BACKGROUND AND RATIONALE

1.1 SARS-CoV-2 Vaccines

The rapid development and subsequent authorisation of vaccines against SARS-CoV-2 has been a major step forward for medical science. In the UK, three vaccines are already approved by the Medicines and Healthcare products and Regulatory Agency (MHRA), the COVID-19 mRNA Vaccine BNT162b2 (Pfizer/BioNTech), COVID-19 Vaccine AstraZeneca (formerly AZD1222), and the mRNA vaccine developed by Moderna (COVID-19 Vaccine Moderna). It is likely that further vaccines will become available in the coming months. National vaccination programmes have been initiated in the UK for Pfizer-BioNtech and the Astra-Zeneca vaccines. The populations evaluated in the trials of these vaccines were generally healthy volunteers. Therefore questions remain as to the level of protection these vaccines will afford patient populations with chronic illnesses who may have primary or secondary immune deficiencies and, therefore, may not generate the same protective responses observed in healthy volunteers.

This prospective observational trial will investigate the immune responses and protective value of approved SARS-CoV-2 vaccines as they are implemented in the national vaccination programme in patient cohorts with a range of chronic diseases that intrinsically, or as a result of the associated therapies, have impaired immunity. The effectiveness of the immune response and its durability will be assessed.

1.2 Justification for the Trial and Adult Participant Populations

The differential impact of COVID-19 infection in multiple disease groups has been extensively reported over the last 12 months. In particular, patient disease cohorts with end stage kidney disease, liver disease, cancer, immune-mediated rheumatic diseases, and transplant recipients are likely to exhibit altered immune responses to SARS-CoV-2 vaccines either as a function of their underlying disease and associated immune dysregulation or due to their requisite management with immune modifying medications, including biologics, disease-modifying anti-rheumatic drugs (DMARDs), broad spectrum immune suppressants and glucocorticoids.

1.2.1 Cancer

In the field of oncology, there are reports to suggest that some patients with cancer are more likely to be infected by SARS-CoV-2, develop a severe COVID-19 infection and more likely to die as a result of COVID-19. There is wide heterogeneity of this patient population, including the disease type, stage, nature of the (multi-modality) treatment, age and co-morbidities. Multiple prospective cohort studies have identified consistent patterns of groups of patients at risk of more severe outcomes, including patients with lung cancer, haematological malignancies and advanced or active cancers. There is a notable difference between the impact on patients with solid tumours compared to haematological malignancies, especially multiple myeloma (MM) where disease and treatment related immune dysfunction combines with the advanced age to result in significant mortality risk [1-3]. The UK

CRCTU-PRT-QCD-001, version 1.0

Coronavirus Cancer Monitoring project [4, 5] recently updated its analyses and compared patients who died of COVID-19, rather than of all causes. This revealed that patients with MM (N=108; OR 1.99; 1.18-3.34; p=0.010), leukaemia (N=139; OR 2.07; 1.24-3.44; p=0.005), as well as lung cancer (N=201; OR 1.61; 1.02-2.56; p=0.041) were at significantly increased risk of death (Middleton, manuscript in preparation). The impact of systemic anti-cancer chemotherapy *per se* is less clear but factors associated with adverse outcome in the general population, including male sex, increasing age, comorbidities, poor performance status, and smoking are also negative risk factors in the cancer patients.

There is clear vulnerability of patients with cancer to COVID-19, which therefore increases the importance of vaccination to this patient group. However, their reduced immune-competence may also compromise their response to SARS-CoV-2 immunisation. Indeed, the same factors that increase risk of COVID-19 in these patients may also reduce their immune response to SARs-Cov-2 vaccines. Studies have demonstrated that the immunogenicity of influenza vaccines is lower in patients with cancer compared to healthy individuals [6]. Population studies demonstrate that influenza vaccine effectiveness is only 25% in patients with solid tumours and only 8% in patients with haematological malignancies. The humoral immunogenicity of influenza vaccination was less in patients receiving cytotoxic chemotherapy compared to those receiving immune checkpoint inhibitors [5].

1.2.1.1 Solid Tumours

1.2.1.1.1 Breast Cancer

Breast cancer is the commonest cancer in women, affecting up to 1 in 7 women with 55,000 new cases per year in the UK and causing 11,000 deaths per year [7]. Treatment for both early and advanced disease is complex and multimodal involving surgery, radiotherapy, chemotherapy and targeted therapies including endocrine therapy, anti HER-2 therapies, immunotherapy and other treatments such as CDK inhibitors and m-TOR inhibitors. Treatment for early disease is often protracted with immunosuppressive treatments for up to 6 months and many adjuvant therapies extending over many years. In advanced disease, patients can be on myelosuppressive treatment for very protracted periods often extending over several years. Understanding the effectiveness of the SARS-CoV-2 vaccine in breast cancer patients undergoing treatment in both early and advanced disease settings is of great importance.

1.2.1.1.2 Lung Cancer

There are nearly 48,000 new cases of lung cancer per annum in the UK making it the second most common cancer in both women and men. The incidence is highest in the 85-89 age group, a demographic highly associated with poor outcomes following COVID infection [5] and 44% are diagnosed in those aged 75 or above. Although the outcome for the majority of patients with advanced inoperable disease used to be uniformly poor, both targeted therapy and immunotherapy have transformed the outcome for numerous patients with the prospect of long-term survival for many. COVID-19 represents a particularly ominous threat to patients with lung cancer and they are a cancer group that arguably more than any other might benefit from vaccination to protect against COVID

infection. The UK Coronavirus Cancer Monitoring project compared the case fatality rate of patients with different solid cancers and demonstrated that patients at significantly increased risk of death as a result of COVID infection were those with lung cancer (n=227; OR 1.62; 95% CI 1.07-,2.46; p=0.023) compared to digestive tract cancer patients. Of the 227 lung cancer patients with valid outcome, age, gender and comorbidities, there were 113 patients who received no treatment, 45 patients who received chemotherapy, 34 patients who received immunotherapy, and 32 patients who received radiotherapy in the 4 weeks before a COVID-19 diagnosis. Multivariate analysis adjusted for age, gender and comorbidities, revealed lung cancer patients who received immunotherapy in the 4 weeks before a COVID-19 diagnosis had a significantly lower risk of death than those who did not receive immunotherapy (N=34; OR 0.40; 0.18-0.89; p=0.025). On the other hand, lung cancer patients who received chemotherapy in the 4 weeks before a COVID-19 diagnosis had no significantly different outcome compared to those patients who did not receive chemotherapy (N=45; OR 0.698; 0.35-1.37; p=0.290). Similarly, lung cancer patients who received radiotherapy in the 4 weeks before a COVID-19 diagnosis did not have a significantly different outcome compared to those patients who did not receive radiotherapy (N=32; OR 1.03; 0.48-2.23; p=0.942).

In a study of outcomes of unplanned hospital admissions of patients with non-small-cell lung carcinoma (NSCLC), 36% were a direct result of pneumonia with an in-patient mortality of 43.9% compared with 13.1% for all other causes [8]. Crucially, a positive 5-year pneumovax status was protective with an odds ratio of 0.16. In the pneumonia cohort, pneumovax status also significantly reduced mortality with the odds ration being 0.269 in multivariable analysis. For patients admitted to hospital with pneumonia without previous pneumovax vaccination in the past 5 years the odds of death were almost 60-fold higher compared with those that had been vaccinated.

In summary lung cancer, the second commonest cancer in men and women, is associated with significantly increased risk of death after COVID infection which does not appear to be related to chronic obstructive pulmonary disease, smoking or treatment. The protective benefit of pneumovax vaccination strongly supports a programme of prophylactic COVID vaccination in these patients but the efficacy of such vaccination in engendering an immune response against the virus, will be explored in the OCTAVE trial.

1.2.1.2 Malignant Haematology

In a systematic review of the outcome of blood cancer patients with COVID-19, hospitalised patients have a high risk of death (pooled risk estimate 36%). Older patients experience higher mortality, and paediatric patients appear to be relatively spared [1]. All subgroups of haematological malignancies had high risks of overall mortality: acquired bone marrow dysfunction syndromes 57% (95% CI 42-72, 11 studies, 42 patients); leukaemias 44% (95% CI 31-58, 15 studies, 159 patients), MM 38% (95% CI 29-47, 18 studies, 387 patients); lymphomas (including chronic lymphocytic leukaemia (CLL)) 32% (95% CI 26-38, 16 studies, 696 patients); lymphomas (excluding CLL) 32% (95% CI 18-48, 11 studies, 156 patients); CLL 31% (95% CI 24-39, 13 studies, 457 patients) and myeloproliferative neoplasms 37% (95% CI 25-49, 9 studies, 62 patients). Importantly, based on the observational data available to date, recent cancer treatment does not appear to significantly increase the risk of dying. These data highlight the need for robust strategies to prevent patients with hematologic malignancy from contracting COVID-19.

1.2.1.2.1 Multiple Myeloma

Patients with MM, a malignancy of terminally differentiated B-cells, may be at particularly high risk from COVID-19 owing to the significant disease and treatment-related immune dysfunction present throughout the disease course. This disordered immunity is multifactorial and progressive with advancing disease, affecting innate and humoral compartments [9-13]. Occurring in a predominantly elderly population, often with multiple co-morbidities, this MM-associated immune dysfunction is additive to a background of age-related immunodeficiency and immunosenescence [14]. This is further compounded by the immunosuppressive and immunomodulatory impact of therapy, particularly autologous haemopoietic stem cell transplant (ASCT). Despite marked improvements in MM outcomes in the last 20 years, infection remains a leading cause of death, with MM patients rendered particularly susceptible to adverse outcome from both bacterial and viral respiratory tract infections [15, 16]. Responses to vaccination are frequently inadequate or short-lived. Whilst data regarding novel coronavirus vaccines in MM patients are lacking, it is likely that the impact of COVID-19 on patient management will continue until there is a significant background level of vaccination-mediated herd immunity.

Published studies on outcome in MM patients with COVID-19 are limited and drawn from early experience in the pandemic. The Spanish Myeloma Collaborative Group have reported the largest cohort to date (167 inpatients from 67 centres), though this was from early in the first wave (patients experiencing COVID-19 from 1st of March to 30th of April 2020). Mortality was 34% in MM patients, compared to 23% in non-cancer patients, rising to 42% in those >65 years and 49% for those with active or progressive disease [17]. Datasets from New York indicated a mortality rate of 30% for patients requiring in-hospital management. Interim analysis of the UK early first wave experience was published in June 2020, where the highest reported mortality of 55% was seen, rising to 71% in those >80 years [3].

1.2.1.2.2 Acute Myeloid Leukaemia

Acute myeloid leukaemia (AML) is the second commonest adult haematological malignancy and its incidence rises to more than 30 per 100,000 in patients over 70 years of age. In fit adults under the age of 75 intensive chemotherapy, consolidated by allogeneic hematopoietic stem cell transplant (allo-HSCT), represents an important curative strategy [18]. In older patients, venetoclax, in combination with azacitidine has emerged as the new standard of care [19]. In both populations, standard treatment options including potently myelosuppressive and immunosuppressive drug therapies are compounded in many patients by the additive immuno-paretic effects of the allo-HSCT. It is, therefore, no surprise that registry-based studies have identified an increased rate of both COVID-19 infection and mortality in adults with AML [20, 21]. Further prospective studies examining both the clinical course of COVID-19 infection and its immunological sequelae in adults with AML are required and Birmingham's CRCTU Trials Acceleration Programme has already recruited 193 patients to a national prospective non-interventional study (PACE) addressing this issue. Given the prolonged courses of therapy mandated for both younger and older adults with AML, it is vital that COVID-19 vaccination strategies are optimised with specific reference to timing in relation to chemotherapy, impact of specific chemotherapy regimens, including venetoclax based regimens in older patients. Vaccine

efficacy also needs to be studied in the sizeable population of patients with AML who subsequently undergo allo-HSCT.

1.2.2 Rheumatic and Inflammatory Diseases

Patients with immune-mediated rheumatic diseases require a range of long-term systemic immunosuppressive therapies to control their inflammatory diseases and are at increased risk of infections in general, which is increased further by these therapies [22-25]. This established vulnerability is now being realised in the context of COVID-19. These patients are at greater risk of infection, hospitalisation and death, with their therapies frequently predicting severe outcome [26-28]. Furthermore, many of these therapies are known to be associated with attenuated responses to influenza and other existing vaccines [29-31] and such patients were excluded from the existing studies of SARS-CoV-2 vaccines. Therefore, both the diseases and the therapies used to treat them may impact on response and require evaluation in this setting.

The immune-mediated rheumatic diseases are a heterogeneous group of conditions treated with a range of broad and targeted immunosuppressive therapies, which are likely to have differing impacts on SARS-CoV-2 infection and vaccination. It is not possible to study in detail all therapies or conditions, individually or in combination. Therefore, this trial will use a two-tiered approach in order to address the key research questions in detail for the most clinically relevant therapies and also to obtain information to understand the effects on the wider community of patients with immune-mediated inflammatory disorders (IMID) in a real-world setting.

The rheumatic and IMID component of the trial will recruit patients with inflammatory arthritis including rheumatoid arthritis (RA), seronegative and psoriatic arthritis (PsA) treated with methotrexate or tumour necrosis factor (TNF) inhibitors and patients with anti-neutrophil cytoplasm antibodies (ANCA)-associated vasculitis (AAV) treated with rituximab. Methotrexate and TNF inhibitors are the most widely prescribed conventional synthetic and biologic DMARDs in clinical practice in the UK and globally for inflammatory arthritis and a range of non-rheumatic IMID. Both therapies have been reported to be associated with reduced responses to existing vaccines [29, 30]. In order to minimise variability, these therapies will be evaluated in the context of inflammatory arthritis. Rituximab (anti-CD20 monoclonal antibody) is widely used for inflammatory rheumatic conditions, particularly in AAV where it is established as induction and maintenance therapy. Rituximab is of particular importance for SARS-CoV-2 vaccines in light of its B cell depletion and previous evidence of reduced humoral response to influenza vaccines [31, 32].

In addition, a wider clinical cohort of patients with rheumatic and inflammatory diseases who are being treated with these and other immunomodulatory therapies will be included for investigation of their serological response to the SARS-CoV-2 vaccines.

1.2.3 Chronic Renal Diseases

End-stage kidney disease (ESKD) is one of the strongest risk factors for severe COVID-19 (estimated hazard ratio for death 3.69) [33], and ESKD patients hospitalised with COVID-19 have a mortality of approximately 30% [34-37]. The UK renal registry and NHS Blood and Transplant have reported UK

CRCTU-PRT-QCD-001, version 1.0

national mortality rates of 19% in haemodialysis (HD) patients, and over 25% in renal transplant patients with COVID-19 [28, 38].

ESKD patients have a high prevalence of vascular and cardiometabolic disease (e.g. hypertension, ischaemic heart disease, diabetes), either as a result of the underlying cause of their renal disease and/or as a consequence of renal failure. In addition, ESKD results in both relative immunosuppression and chronic low-grade inflammation [39-44], which may impact viral defence and the host inflammatory response. As well as being enriched for cardio-metabolic risk factors, the ESKD group being studied at Imperial College NHS Trust also has a high proportion of Black, Asian, Minority Ethnic (BAME) origin patients. Of the patients sampled in this on-going study, 72% are non-white, including 17% of African or Afro-Caribbean origin and 42% of South Asian origin. Patients in these subgroups are at higher risk of death from COVID-19 pneumonia.

Poor seroconversion is well-documented following standard vaccinations amongst haemodialysis patients [33] and we have observed a high rate of recurrent SARS-CoV-2 infection in haemodialysis (HD) patients. Current medications have not improved SARS-CoV-2 -related mortality in these cohorts [38], and HD patients cannot easily socially distance when required to attend dialysis units several times per week [45].

1.2.4 Chronic Liver Diseases or with Gastrointestinal Disease on Immune Suppressive Therapy

Patients with chronic liver disease (CLD) and especially with cirrhosis have multiple mechanisms of immune dysfunction that can lead to increased susceptibility to infections and an increased inflammatory response during infection. Collectively known as cirrhosis associated immune dysfunction (CAID), there are alterations in the complement system, macrophage, lymphocyte and neutrophil activity, altered toll like receptors, and increases in intestinal dysbiosis [33, 35]. A recent international observational study (covid-hep.net) recruited >1200 patients with chronic liver disease and showed that baseline liver disease stage and alcoholic liver disease are independent risk factor for death from COVID-19 compared to age matched controls patients. [46]. Additional studies have shown that liver transplant recipients are more likely to require Intensive Care Unit admissions following SARS-CoV-2 infection. [45,[47] Patients with cirrhosis also have been shown to have an attenuated response to existing vaccines.[48, 49], however, patients with significant liver disease largely not been included in existing studies of SARS-CoV-2 vaccines.

Patients with inflammatory bowel disease may show an attenuated response to vaccination as disease control often requires prolonged therapy with immune suppressive and biological therapies. Evidence exists mostly on influenza vaccination which suggests a lower serological response in patients on Infliximab (anti-TNF) therapies. Evidence addressing other types of vaccines and therapies such as vedolizumab and ustekinumab is scarce.

1.2.5 Haematopoietic Stem Cell Transplant Recipients

Haematopoietic stem cell transplant (HSCT) is a potentially curative treatment for a range of malignant and non-malignant haematological conditions. Autologous (auto-) HSCT may induce periods of long-term remission in refractory autoimmune conditions. Reported outcomes of COVID-19 in patients

treated with HSCT are limited to case series, but this group appears to be highly susceptible with mortality up to 30% [50, 51]. Autologous (ASCT) and allo-HSCT recipients are immunocompromised for months to a year or more following the procedure, with quantitative and qualitative defects in the innate and adaptive immune response. In the pre-COVID-19 pandemic era, infections accounted for up to 10% of all-cause mortality following allo-HSCT [52]. Measures to reduce infection post-HSCT include physical infection control procedures, prophylactic antimicrobials and immunisation against vaccine preventable diseases. Antibody and T-cell responses to a range of vaccines are reported from 3 months post-HSCT respectively [53, 54], however the corresponding level of clinical protection against most vaccine preventable diseases in this population is unclear. The immunogenicity of the SARS-CoV-2 vaccine technologies in HSCT recipients is unknown, therefore prospective evaluation of vaccine immunogenicity and exploration of the patient, transplant and donor characteristics that impact this will inform optimisation of SARS-CoV-2 vaccine timing post-HSCT.

1.3 Justification for the Trial and Children and Adolescent Participant Populations

The rollout of COVID-19 vaccines to healthy 12 to 15-year-olds began in England Scotland, Wales and Northern Ireland in 2021 and roll out to children who are immune suppressed started in summer 2021 in all four nations. The rollout of COVID-19 vaccines to immune suppressed 5-11-year-old children began in February 2022 in all four nations.

To the best of our knowledge there are no data to inform vaccine schedules in children and young people aged 5 to <18 years who are immune suppressed due to medication following solid organ transplantation or with immune-mediated rheumatic diseases or due to treatment for cancer or cancer itself. Moreover, these patients were excluded from the existing studies of SARS-CoV-2 vaccines in those aged between 5 and 17 years and 364 days.

1.3.1 Solid Organ Transplant

Responses to influenza vaccine are diminished in recipients of solid organ transplantation, with evidence that additional dosing improves measurable antibody responses [55]. Early reports in adults following Pfizer-BioNTech SARS-CoV-2 vaccination show that antibodies can be detected in 17% of solid organ transplant recipients after the first dose, and in 54% of patients after the second dose [56]. The proportion of adults making measurable antibody responses appears to be greater in the younger age groups.

1.3.2 Immune-mediated Rheumatic Diseases

Patients with immune-mediated rheumatic diseases require a range of long-term systemic immunosuppressive therapies to control their inflammatory diseases. Safe and effective vaccination is important in patients with rheumatic and inflammatory diseases, given the increased risks of infections [57]. The host immune system might influence vaccine efficacy. Therefore, both the diseases and the therapies used to treat these children and young people may impact on response and require evaluation in this setting. Limited evidence suggests that paediatric rheumatic diseases and immunosuppressive therapies are associated with attenuated serological responses vaccines [58-60], and the generalisability of these studies is unclear.

1.3.3 Childhood Cancer

Cancer is rare during childhood, adolescence and young adulthood. In the UK, there are approximately 1,645 children aged 0-14 years and 2,110 teenagers and young adults aged 15-24 years diagnosed with cancer each year. Overall, 86% of children and young people survive for at least five years [61]. Children who receive treatment for cancer become significant immunosuppressed as a result of the treatment and the underlying diagnosis and are therefore at increased risk of serious infections [62]. In the UK, when relatively little was known on how these children and young people might be affected by COVID-19, national guidance advised stringent shielding measures which had a significant impact on the physical, psychosocial and financial wellbeing of many families [63].

Later evidence emerged that children with cancer in the UK with SARS-CoV-2 infection were not at an increased risk of severe disease when compared to the general paediatric population [64, 65]].

It is unclear how children with cancer may respond to the SARS-CoV-2 vaccine and how long any response might last. For influenza vaccines, it is known that children receiving chemotherapy generate a weaker immune response than healthy children [66]. The clinical implications of testing SARS-CoV-2 vaccine responses in children with cancer will enable evidence-based recommendations to be made in terms of underlying diagnosis, timing and will help indicate whether booster doses will be required.

2. AIM, OBJECTIVES AND OUTCOME MEASURES

The aim of this trial is to evaluate the immune response to SARS-CoV-2 vaccination in clinically vulnerable groups across the UK.

2.1 Objectives

2.1.1 Primary Objective

- To determine the magnitude of the immune response to SARS-CoV-2 vaccines in participants with chronic diseases and/or secondary immunodeficiency

2.1.2 Secondary Objectives

- To determine phenotype and function of SARS-CoV-2 vaccine induced immune responses in participants with chronic diseases and/or secondary immunodeficiency, compared to each other and healthy controls in parallel studies
- To evaluate the impact of distinct immune therapeutic drug classes on the development of humoral and cellular immune responses to SARS-CoV-2 following vaccination

2.2 Outcome Measures

2.2.1 Primary Outcomes

2.2.1.1 Vaccine specific Immunogenicity:

- Anti-SARS-CoV-2 IgG Abs following vaccination will be measured using the Roche platforms by the UK Health Security Agency formerly known as Public Health England Laboratories at Porton Down. The Roche assay measures the presence and amount of serum antibodies to both the spike (S) and the nucleocapsid (N) antigens of SARS-CoV-2. This assay will enable the discrimination of IgG responses to SARS-CoV-2 that results from vaccination and/or SARS-CoV-2 infection.
- T cell responses to SARS-CoV-2 peptides following vaccination will be measured using the Oxford Immunotec modified T-SPOT Discovery SARS-CoV-2 assay. This IFNg ELISpot assay will provide insights into the participants' reactivity to SARS-CoV-2 S1, S2, Nucleocapsid and membrane peptides.

2.2.2 Secondary Outcome

2.2.2.1 Clinical protection

- First symptomatic, PCR-proven COVID-19 occurrence from 14 days after first dose of SARS-CoV-2 vaccine in participants without evidence of prior infection with SARS-CoV-2

2.2.3 Exploratory Outcomes

2.2.3.1 Humoral Immunogenicity

- SARS-CoV-2 IgG (pseudo)neutralisation assays to assess the capacity of vaccine induced SARS-CoV-2 Abs to neutralise/block SARS-CoV-2 infection

2.2.3.2 Cellular Immunogenicity

- The relative contribution of T cell subsets and T cell function will be assessed using intracellular cytokine analysis and flow cytometry (ICCS) - established at Oxford University laboratories (https://www.biorxiv.org/content/10.1101/2020.06.05.134551v1)
- Proliferation assays (CTV assay)) will evaluate the recall potential of SARS-CoV-2 memory T cells at later (6 month) time points (established at Oxford University laboratories https://www.biorxiv.org/content/10.1101/2020.06.05.134551v1)

Additional assays relevant to immune state and response may be undertaken:

- Serum antibodies (IgG/IgM/IgA) to important SARS-CoV-2 antigens and SARS-CoV-2 related antigens (including but not limited to SARS, MERS and circulating seasonal coronaviruses: CoV-2 S, NL63 S, CoV-2 N, CoV-1 S, MERS S, HKU1 S, OC43 S, 229E S, CoV-2 RBD) will be measured in an MSD assay or bespoke ELISA established at University of Oxford laboratories

- Saliva antibodies (IgG/IgA) to both the spike (S) and the nucleocapsid (N) antigens of SARS-CoV-2 will be measured using an optimised saliva ELISA developed by the University of Bristol
- Flow cytometric characterisation of the circulating immune compartment (e.g. T cells and B cells) will be undertaken
- T cell and B cell specific responses to defined peptides/stimuli will be undertaken using established ELISpot assays, at Imperial College London and Oxford University laboratories

Furthermore, other assays may be added as data emerges from OCTAVE and other related studies.

OCTAVE will include an optional additional blood sample 1-day post-vaccine that can be requested from trial participants primarily for next generation sequencing approaches (e.g., RNA sequencing) to evaluate in detail early innate responses at this time point.

3. TRIAL DESIGN

The OCTAVE trial is a multi-centre, multi-disease, prospective observational cohort trial of the immune response to SARS-CoV-2 vaccination. We will evaluate immunity arising from receipt of the COVID-19 Vaccine AstraZeneca, COVID-19 mRNA Vaccine BNT162b2 and COVID-19 Vaccine Moderna or other MHRA approved SARS-CoV-2 vaccines implemented in the UK. Participants with end stage kidney disease, liver disease, cancer, immune-mediated rheumatic diseases, and transplant recipients attending specialist clinics across the UK will be recruited. If funding permits additional disease cohorts may also be added by protocol amendment if scientifically and clinically relevant to the objectives of the trial.

Cohorts of approximately 100 to 200 adult participants depending on disease group will be recruited for full immune response analysis (“Deep Immunophenotyping Group”). Additional adult participants will be recruited, between 150 and 850 depending on disease group, for serology analysis (“Serology Group”).

Up to 160 children and young people aged 5 to 17 years and 364 days, inclusive (aged under (<) 18 years) will be recruited to the “Serology Plus Group”.

Up to 3250 participants will be recruited in total.

Additional vaccine response studies are ongoing across the UK, including Department of Health and Social Care funded analyses of responses in health care workers, care homes, and in BAME groups. Through use of common immune assay platforms, our data will be directly comparable with these emerging datasets allowing comparison with matched controls.

4. ELIGIBILITY

Patients meeting the criteria below are eligible to participate in the trial.

4.1 Inclusion Criteria

1. Are eligible for vaccination by one of the SARS-CoV-2 vaccines approved by the MHRA administered in accordance with national guidelines and current versions of the applicable information for healthcare professionals (see Section 7.1) and:
    - For the **Deep Immunotherapy Group** only, have not received the second dose of the vaccine (booster)
    - For the **Serology Group** only, have not passed the 28 days (-7/+56 days) post second vaccine dose (booster)
    - For the **Serology Plus Group,** have not passed 28 days (within -7/+56 days) post second vaccine dose (booster) or up to 6 months post second vaccine dose, including patients who have received a third or further dose in that time period
2. Anticipated life expectancy of 6 months or greater
3. Fall into one (or more) of the following patient cohorts who will meet disease relevant classification, disease state, and staging according to established international standards:
    - **Diagnosed with any of the following malignancies**:
        - Breast
        - Lung
        - Acute Myeloid Leukaemia
        - Multiple Myeloma
        - Paediatric Cancer: any diagnosis of cancer in a child (aged 5 to < 18 years):
            - On active treatment
            - Within 6 months of completion of treatment

    - **Diagnosed with the following rheumatic/inflammatory conditions:**
        - Specialist diagnosis of relevant condition
        - Established on relevant therapy for ≥ 30 days
        - Meet the definitions in any of the following cohorts:
            - Deep Immunophenotyping Group::
                - Methotrexate plus inflammatory arthritis (to include RA, PsA, seronegative arthritis, and spondyloarthritis)
                - TNF inhibitors (any) plus inflammatory arthritis (to include RA, PsA, seronegative arthritis, spondyloarthritis)
                - Rituximab in patients with AAV
            - Serology Group:
                - Methotrexate plus:
                    - inflammatory arthritis (RA, seronegative arthritis and PsA)

        - psoriasis
    - TNF inhibitors (any) plus:
        - inflammatory arthritis (RA, seronegative arthritis, axSpA and PsA)
        - psoriasis
        - Crohn's disease
    - IL-17 inhibitors (any), IL-12/23 inhibitors and IL-23 inhibitors plus:
        - seronegative arthritis (PsA and axSpA)
        - psoriasis
    - IL-6 inhibitors (any) with RA
    - JAK inhibitors (any) with RA
    - Rituximab with RA or AAV
    - Any immune modifying treatment with Systemic Lupus Erythematosus (SLE)
- Serology Plus Group: aged 5 to <18 years at time of recruitment and
    - Methotrexate plus inflammatory arthritis with onset under the age of 16 years (also known as juvenile idiopathic arthritis JIA), with or without JIA-uveitis
    - TNF inhibitors (any) plus inflammatory arthritis with onset under the age of 16 years (JIA), with or without JIA-uveitis
    - IL-6 inhibitors (any) plus inflammatory arthritis with onset under the age of 16 years (JIA), with or without JIA-uveitis
    - Any immune modifying treatment with juvenile onset Systemic Lupus Erythematosus (JSLE)
    - Rituximab with plus inflammatory arthritis with onset under the age of 16 years (JIA), with or without JIA-uveitis or AAV

- **Diagnosed with the following chronic renal conditions:**
    - End stage kidney disease secondary to any cause
    - Renal transplant following end stage kidney disease

- **Diagnosed with the following chronic liver conditions:**
    - Liver cirrhosis
    - Liver transplantation
    - Chronic liver disease (of any stage), or gastrointestinal disease on immune suppressive therapy

- **Haematopoietic stem cell transplant patients:**
  - Previously treated with autologous or allo-HSCT for any indication and with any conditioning regimens and intensities
  - Previously treated with CAR-T cell therapies

  Note: HSCT and CAR-T recipients who have received one or two doses of a SARS-CoV-2 vaccine pre-procedure and are receiving re-vaccination post HSCT / CART-T are eligible for recruitment at:-

    - Baseline (prior to re-vaccination dose 1) to either Deep Immunophenotyping Group or Serology Group
    - For the Deep Immunophenotyping Group: before they received the second re-vaccination dose (booster)
    - For the Serology Group: up to 28 (-7 /+ 56) days post second re-vaccination (booster) only if 2 doses have been administered post-HSCT / CAR-T procedure.

- **Post solid organ transplant in 5 to <18 year olds to the Serology Plus group**
  - Post heart, lung, heart-lung, or kidney transplantation and on immune suppressing medication (calcineurin or mTOR inhibitor, plus additional agents)

5. SCREENING AND CONSENT

5.1 Screening

Participants will be identified from existing clinical databases or via specialist clinics. They will be recruited at the clinical site by members of the clinical team who have been delegated this responsibility on the Site Signature and Delegation Log by the Principal Investigator.

5.2 Informed Consent

It is the responsibility of the investigator or designee (e.g. registrars, Research Nurses if local practice allows and this responsibility has been delegated by the Principal Investigator) to obtain written informed consent for each participant before any trial related procedures. Where this responsibility has been delegated, this must be explicitly stated on a Site Signature and Delegation Log (SSDL). Parent/Guardian and Participant Information Sheets (PIS) are provided to facilitate this process.

Investigators must ensure that they adequately explain the aim of the trial and what the trial would involve for the participant to the patient/parent/legal guardian. The investigator should also stress that the patient/parent/legal guardian is completely free to refuse to take part or withdraw from the trial at any time. The patient/parent/legal guardian should be given ample time (ideally 24 hours) to read the Participant Information Sheet and to discuss their participation with others outside of the site

research team should they wish to do so. The patient/parent/legal guardian must be given an opportunity to ask questions which should be answered to their satisfaction. The right of the patient/parent/legal guardian to refuse to participate in the trial without giving a reason must be respected.

The informed consent process is expected to involve an interview between member(s) of the investigator team and the patient/parent/legal guardian which should facilitate two-way communication. It is possible for this interview to be conducted remotely. Where this occurs, the patient/parent/legal guardian can be sent the Participant Information Sheet(s) in advance in the post or electronically. If the patient/parent/legal guardian agrees to participate in the trial, they should be asked to sign and date the latest version of the Informed Consent Form. Written assent will also be obtained from patients under the age of 16 years wherever it is possible to do so using the relevant section on the Parent ICF. The Informed Consent Form should either be wet-ink signed by the patient, parent or legal guardian and the investigator (or designee) or signed electronically using software which allows signature authentication (e.g. DocuSign). If wet-ink signed, the Informed Consent Form can be returned when the patient attends for their first clinic appointment or it can be returned in the post, but it must be signed by both parties prior to the patient's entry into the trial.

Once the patient is entered into the trial, the participant's trial number should be entered on the Informed Consent Form. A copy of the Informed Consent Form should be given to the participant or parent/legal guardian, a copy should be filed in the hospital notes, a copy sent to the Trial Office and the original placed in the Investigator Site File.

Details of the informed consent/assent discussions should be recorded in the patient's medical notes; including date of, and information regarding the initial discussion, the date consent was given, with the name of the trial and the version numbers of the Participant Information Sheet and Informed Consent Form.

Throughout the trial the participant/parent/legal guardian should have the opportunity to ask questions about the trial and any new information that may be relevant to the participant's continued participation should be shared with them in a timely manner. On occasion it may be necessary to re-consent the participant, in which case the process above should be followed and the participant/parent/legal guardian's right to withdraw from the trial respected. Participants/parents/legal guardians are permitted to re-consent at the same visit that new information is provided if they wish to do so. Details of these discussions (as specified above) should also be recorded in the patient's medical notes.

Electronic copies of the Participant Information Sheet and Informed Consent Form are available from the Trial Office and should be printed or photocopied onto the headed paper of the local institution.

Patients turning 16 years should be re-consented to the adult versions of the Participant Information Sheet and Informed Consent Form. A cover letter for the participant (approved by the General Review Committee (GRC)) is available to aid in the re-consent process.

6. TRIAL ENTRY

Adult participants in the Deep Immunotherapy Group, should enter before receipt of their second dose of the vaccine; while those in the Serology Group should not have not passed the 28 days (-7/+56 days) post second vaccine dose.

Children and Adolescent participants can enter prior to their first, second or third vaccine doses.

After screening, the following will be checked prior to recruitment:

- Participant/parent/legal guardian has provided consent and, where appropriate, patient assent
- Confirmation that the eligibility criteria have been met

If eligibility is confirmed, the patient can be recruited into the trial.

Registration will be conducted by completing the password protected site specific trial entry spreadsheet which should be emailed to the trial mailbox, preferably at the end of each day a participant is recruited. A unique Trial Number will be allocated to each participant, this number should be included on all samples, forms, and correspondence relevant to that participant.

The Trial Office will enter the participants' details onto the trial electronic Remote Data Capture (eRDC) database.

Investigators must be registered with the Trial Office before they are permitted to enter patients into the trial.

The site are asked to confirm the patient's eligibility, and provide the following information:

- Details of person registering the patient
- Patient's initials and date of birth
- Date of consent

The participant's General Practitioner (GP) should be informed that they are taking part in the trial. A GP Letter is provided electronically for this purpose.

Once a participant has been entered into the trial their name and contact details should be added to the Participant Identification Log and a copy of the signed Informed Consent Form should be sent in the post to the Trial Office for internal review.

7. TRIAL REQUIREMENTS

7.1 Investigational Medicinal Products

The following SARS-CoV-2 vaccines are regarded as Investigational Medicinal Products (IMPs) for this trial:

- COVID-19 mRNA Vaccine BNT162b2, manufactured by Pfizer.

Information for healthcare professionals can be found at:
https://www.gov.uk/government/publications/regulatory-approval-of-pfizer-biontech-vaccine-for-covid-19/information-for-healthcare-professionals-on-pfizerbiontech-covid-19-vaccine

- COVID-19 Vaccine AstraZeneca, manufactured by AstraZeneca.

Information for healthcare professionals can be found at:
https://www.gov.uk/government/publications/regulatory-approval-of-covid-19-vaccine-astrazeneca/information-for-healthcare-professionals-on-covid-19-vaccine-astrazeneca-regulation-174

- COVID-19 Vaccine Moderna, manufactured by Moderna.

Information for healthcare professionals can be found at:
https://www.gov.uk/government/publications/regulatory-approval-of-covid-19-vaccine-moderna/information-for-healthcare-professionals-on-covid-19-vaccine-moderna

7.1.1 Pharmacy Requirements

The vaccines will be administered in accordance with its temporary authorisation and national guidelines. Hence there are no trial specific pharmacy requirements. IMP labelling and accountability will not be required for this low risk trial. The batch number (where this is known) of IMP administered to the participant will be collected on the Vaccination Form.

7.2 Vaccination

Vaccine will be administered in line with its temporary authorisation under Regulation 174 of the Human Medicines Regulations 2012, the national recommendations, and guidance of the Joint Committee on Vaccination and Immunisation (JCVI) and current standard NHS practice.

The trial will have no influence on the type of vaccine given to the participant but the type of vaccine and the batch number used will be recorded in the Case Report Form (CRF) where possible.

7.2.1 First Vaccination

Participants enrolled in this trial will be signposted to, or assisted where necessary, in obtaining an appointment for delivery of their first vaccination according to local NHS COVID-19 vaccine delivery options. Participants who are sent unsolicited appointments or make their own independent arrangements for vaccination are still able to participate but sites will endeavour to make their involvement in this trial and the administration of the SARS-CoV-2 vaccine as straight forward as possible, minimising any visits to hospitals or vaccination centres. The operational details for this assistance will be determined at each site.

Once the first vaccination has been delivered, the intended appointment and location for the second vaccine dose (booster) will be recorded and sampling around the second vaccination can be planned.

7.2.2 Second Vaccination (Booster) and Subsequent Vaccine Doses

The trial will have no influence on the timing of the delivery of the second (booster) and subsequent doses, but it is expected that the second and subsequent doses will be delivered in accordance with national recommendations and the guidance of the JCVI.

7.3 Assessments

Assessments should be carried out in accordance with the Schedule of Events.

7.4 Sample Collection and Analysis

7.4.1 Collection

Sites will be provided with kits for the collection of the research samples.

For the **Deep Immunophenotyping Group** up to 55ml of blood will be collected from participants at any one visit comprising whole blood, peripheral blood mononuclear cells (PBMC), serum and plasma. Saliva samples will also be collected where possible. Samples will be collected at the following time points:

- Pre-vaccine (baseline) – this is an optional time point, samples may have been collected prior to recruitment to OCTAVE
- 1 day after first vaccination – this is an optional time point*
- Pre-second vaccine dose (booster)
- 28 days post-second vaccine dose (within +/- 3 days)
- 6 months post second vaccine dose (as close to time point as possible), or prior to third vaccine dose (if applicable), whichever is earlier
- 12 months after first vaccine dose (as close to time point as possible)**

* 3ml Tempus sample and 2-3 x 9ml EDTA samples required at this time point

** 2 x 5ml SST samples required at this time point

For the **Serology Group** as a minimum 10ml of serum will be collected, but where possible whole blood, PBMC, and plasma will also be collected, from participants at the following time points:

- Pre-first vaccine (baseline) – this is an optional time point, samples may have been collected prior to recruitment to OCTAVE
- Pre-second vaccine dose (booster) – this is an optional time point, any time after the first vaccination but before booster
- 28 days post-second vaccine dose (-7/+56 days)

CRCTU-PRT-QCD-001, version 1.0

For the **Serology Plus Group**, a minimum of 5 ml and up to 22 ml of participant's blood will be collected comprising whole blood (Lithium Heparin tube) and serum (SST tube) at the following time points:

- Prior to the vaccine dose being given at trial entry (referred to as baseline (pre-first dose), pre-second vaccine, pre-third vaccine or pre-fourth vaccine as applicable)
- 28 days (-7/+56 days) post vaccination (referred to as post-first dose, post-second dose, post-third dose or post-fourth vaccine as applicable)
- 28 days (-7/+56 days) post vaccination for subsequent doses (optional) (referred to as post-second dose, post-third dose or pre-fourth dose as applicable)

A maximum of four samples will be collected per patient for this group.

See Table 1 for further details on sample types collected and storage conditions.

Full details of the sample collection and processing procedure are included in the Laboratory Manual.

CRCTU-PRT-QCD-001, version 1.0

Table 1: Details of Sample Collection and Storage

Sample Type	Collection Tube	Volume	Laboratory Analysis	Aliquots	Sample Storage
Whole Blood	Tempus RNA	3ml	RNA	None	-80°C
Whole Blood	EDTA	9ml x minimum of 2 (maximum of 3)	Cellular immunoassays & DNA	4 x 500ul (as PBMCs) 5 x 700ul (as Plasma)	-80°C for Plasma (long-term) and PBMCs (short-term), then liquid nitrogen or -150 °C; depends on site (long-term)
Serum	SST	5ml x 2 in those ≥18 years; 1 or 2 tubes in 5-<18 year olds	Immunoassay; ELISA and virus neutralisation assays	10 x 400ul	-80°C
Whole Blood	Lithium Heparin*	6ml x 2 in those ≥18 years; 1 or 2 tubes in 5-<18 year olds	ELISpot assay	None	None
Saliva †	Saliva collection funnel and 10ml collection tube	1 spit up to 1ml mark on collection tube	Immunoassay	1	-80°C

*Two 6ml lithium heparin tubes will be shipped to Oxford Immunotec at ambient temperature. Samples must be received by Oxford Immunotec within 32 hours of collection (see Laboratory Manual for further details).

† Samples should be kept at 4°C until processing and freezing at -80°C or immediate shipment.

Some participants will already have the appropriate baseline samples in storage under other sampling arrangements such as tissue banks or other observational Research Ethics Committee (REC) approved studies. Where these samples are available they can be used for this trial.

The remaining samples will be shipped in batches to the relevant coordinating laboratory. Site will be provided with details of which laboratory to send the samples to at the time of shipment.

7.4.2 Analysis

7.4.2.1 Deep Immunophenotyping Group

Laboratory analysis to be conducted on the Deep Immunophenotyping Group samples will include:

- Assessment of IFN-g T cell responses to SARS-CoV-2 antigens in an ex vivo ELISpot assay, to be undertaken by Oxford Immunotec
- Assessment of fine specificity of T cell and B cell responses (ELISpot and Flow cytometric) will be performed by laboratories at the University of Oxford and Imperial College London

This is not an all-inclusive list; additional assays will be included as more information becomes available about the immune response.

7.4.2.2 Serology Group

Laboratory analysis to be conducted on the Serology Group samples are expected to include:

- Assessment of quantitative IgG responses to SARS-CoV-2 spike and nucleocapsid antigens will be undertaken by the UK Health Security Agency Laboratories at Porton Down
- Assessment of neutralising antibody responses to SARS-CoV-2 antigens be undertaken at both the laboratories at the University of Glasgow and the UK Health Security Agency Laboratories at Porton Down
- Assessment of IgG/IgM/IgA responses to SARS-CoV-2 antigens and other relevant season antigens will be undertaken by the laboratories at the University of Oxford
- Assessment of IgG/IgA responses to SARS-CoV-2 spike and nucleocapsid antigens will be undertaken by laboratories at the University of Bristol

This is not an all-inclusive list, additional assays will be included as more information becomes available about the immune response.

Principal Investigators will be provided with the results of their patients' assessment of immune response. Participants should be provided with the results on request.

7.4.2.3 Serology Plus Group

Laboratory analysis to be conducted on the Serology Plus Group samples are expected to include:

- Assessment of quantitative IgG responses to SARS-CoV-2 spike and nucleocapsid antigens will be undertaken by the UK Health Security Agency Laboratories at Porton Down
- Assessment of neutralising antibody responses to SARS-CoV-2 antigens be undertaken at the laboratories at the University of Glasgow and the UK Health Security Agency Laboratories at Porton Down
- Assessment of IgG/IgM/IgA responses to SARS-CoV-2 antigens and other relevant seasonal antigens – the intent is for these analyses to be carried out at the University of Oxford

- Assessment of IFN-g T cell responses to SARS-CoV-2 antigens in an ex vivo ELISpot assay, to be undertaken by Oxford Immunotec

7.4.3 Future Research and Biobanking

Any samples remaining at the end of the trial will be banked in a Human Tissue Authority (HTA) licenced biobank. The samples and data will be made available for future research in other ethically approved studies (see Section 13.3 and Section 18 for further details).

7.5 Participant Follow-Up

Participants will be followed up in accordance with standard clinical practice for the relevant disease cohort.

Data will be collected retrospectively from clinic records 6 months after the second vaccination (or prior third vaccine dose (if applicable), whichever is earlier) and 12 months after first vaccination.

For the children and adolescent group, data will be collected retrospectively from clinic records 6 months after the participant's most recent vaccine.

Data collected from face-to-face consultations or telephone follow-up calls are acceptable.

7.6 Data Linkage

The participant's NHS number or CHI number will be collected to allow future linkage with national data registries such as NHS Digital, UK Health Security Agency, the Information Services Division (part of NHS Scotland), or the electronic Data Research and Innovation Service (eDRIS). For the HSCT participant group the British Society of Blood and Marrow Transplantation (BSBMT) registry identification (PROMISE ID) will also be collected, where participants have also consented to collection of data within this registry, to allow for data linkage. Data linkage will provide a more complete profile of the participants' health and disease without increased data collection burden to the NHS.

7.7 Participant Withdrawal

Participants may withdraw consent at any time during the trial. For the purposes of this trial two types of withdrawal are defined:

- The participants would like to withdraw from further sample collection but is willing to be followed up as standard (i.e. the patient has agreed that data can be collected at standard clinic visits and used in the analyses)
- The participants would like to withdraw from the trial entirely and is not willing to be followed up for the purposes of the trial (i.e. only data and samples collected prior to the withdrawal of consent can be used in the trial analysis) – withdrawal of consent

The details of withdrawal (date, reason and type of withdrawal) should be clearly documented in the source data. A Withdrawal of Consent Form should be completed to notify the Trial Office of the participant's withdrawal from the trial.

8. ADVERSE EVENT REPORTING

The collection and reporting of Adverse Events (AEs) will be in accordance with the Medicines for Human Use Clinical Trials Regulations 2004 and its subsequent amendments. Definitions of different types of AE are listed in Appendix 1. The seriousness and causality (relatedness) of all AEs experienced by the participant should be assessed with reference to the relevant information for healthcare professionals (see Section 7.1).

8.1 Reporting Requirements

8.1.1 Adverse Events

AEs are commonly encountered by participants with the chronic healthcare conditions being studied in this trial. Hence only Adverse Reactions (ARs) thought to be related to the administration of the SARS-CoV-2 vaccines will be collected.

8.1.2 Serious Adverse Events

Investigators should only report AEs that meet the definition of an SAE and which are thought to be related to the administration of the SARS-CoV-2 vaccine. These events should be reported on a SAE Form as described in Section 8.3.1.2.

8.2 Reporting Period

Details of AEs will be documented and reported from the date of recruitment into the OCTAVE trial and only after the initial vaccination of SARS-CoV-2 vaccine until 28 days after the administration of the most recent vaccine.

SAEs that are judged to be at least possibly related to the IMP and are unexpected must still be reported in an expedited manner irrespective of how long after IMP administration the reaction occurred.

8.3 Reporting Procedure

8.3.1 Site

8.3.1.1 Adverse Events

For more detailed instructions on AR reporting refer to the CRF Completion Guidelines contained in the Investigator Site File.

ARs experienced following vaccination should be recorded on the Vaccination Form.

ARs will be graded using the Common Terminology Criteria for Adverse Events (CTCAE), version 4.03 (see Appendix 2).

Any ARs experienced by the participant but not included in the CTCAE should be graded by an investigator and recorded using a scale of (1) mild, (2) moderate or (3) severe.

For each AR, the highest grade should be recorded.

8.3.1.2 Serious Adverse Events

For more detailed instructions on SAE reporting refer to the SAE Form Completion Guidelines contained in the Investigator Site File.

AEs defined as serious which are thought to be related to SARS-CoV-2 vaccination and which require reporting as an SAE should be reported on an SAE Form. When completing the form, the investigator will be asked to define the causality and the severity of the AE which should be documented using CTCAE version 4.03.

The form should be emailed to the Trial Office as soon as possible and no later than 24 hours after first becoming aware of the event:

Send SAEs to

Reg@trials.bham.ac.uk
Cc OCTAVE@trials.bham.ac.uk
Include "OCTAVE SAE" in the subject line

On receipt, the Trial Office will allocate each SAE a unique reference number. The site will be informed of the SAE reference number in an email acknowledging receipt of the event. If confirmation of receipt is not received within 1 working day please contact the Trial Office. The SAE reference number should be quoted on all correspondence and follow-up reports regarding the SAE. The email from the Trial Office acknowledging receipt should be filed with the SAE Form in the Investigator Site File.

For SAE Forms completed by someone other than the investigator the investigator will be required to countersign the original SAE Form to confirm agreement with the causality and severity assessments. The form should then be returned to the Trial Office and a copy kept in the Investigator Site File.

Investigators should also report SAEs to their own Trust or Health Board in accordance with local policy.

Provision of follow-up information

Participants should be followed up until resolution or stabilisation of the event. Follow-up information should be provided on a new SAE Form (refer to the SAE Form Completion Guidelines for further information).

8.3.2 OCTAVE Trial Office

On receipt of an SAE Form, causality and expectedness will be determined by a Clinical Coordinator. An SAE judged by the investigator or Clinical Coordinator to have a reasonable causal relationship with the trial medication will be regarded as a Serious Adverse Reaction (SAR). If the event meets the definition of a SAR that is unexpected (i.e. is not listed in the Reference Safety Information) it will be classified as a Suspected Unexpected Serious Adverse Reaction (SUSAR).

8.3.3 Reporting to the Competent Authority and Research Ethics Committee

8.3.3.1 Suspected Unexpected Serious Adverse Reactions

The Trial Office will report a minimal data set of all individual events categorised as a fatal or life threatening SUSAR to the MHRA and REC within 7 days. Detailed follow-up information will be provided within an additional 8 days.

All other events categorised as SUSARs will be reported within 15 days.

8.3.3.2 Serious Adverse Reactions

The Trial Office will report details of all SARs (including SUSARs) to the MHRA and REC annually from the date of the Clinical Trial Authorisation (CTA), in the form of a Development Safety Update Report (DSUR).

8.3.3.3 Adverse Events

Details of all AEs will be reported to the MHRA on request.

8.3.4 Reporting to Investigators

Details of all SUSARs and any other safety issue which arises during the course of the trial will be reported to Principal Investigators. A copy of any such correspondence should be filed in the Investigator Site File.

9. DATA COLLECTION

The CRF will be comprised of the forms listed in Table 2.

Table 2: OCTAVE Trial Case Report Form

Form	Summary of Data Recorded*	Schedule for Submission to Trial Office
Registration	Minimal identifiers (initials and date of birth)	Complete at trial entry
Baseline	NHS/CHI/ BSBMT registry ID, hospital number, demographic data (sex, ethnicity), World Health Organisation (WHO) performance status (Appendix 3), body mass index, medical history including comorbidities, disease group specific information (including disease status), details of prior COVID-19 infection, collection of baseline research samples	Within 1 month of trial entry

Form		Summary of Data Recorded*	Schedule for Submission to Trial Office
Treatment Form	First vaccination	Details of rituximab, corticosteroids, and nonsteroidal anti-inflammatory drugs (NSAIDs) and other disease cohort specific treatments	As soon as possible after relevant vaccination
	Second vaccination (booster)		
	Third vaccination etc.		
Vaccination form	First vaccination	Date of vaccination<br>Type of vaccination<br>Batch number (if known)<br>Planned date of second vaccination (booster) (if known)	As soon as possible after relevant vaccination
	Second vaccination (booster)	Date of second vaccination(booster)<br>Type of vaccination<br>Batch number (if known)	
	Third vaccination etc.	Date of third dose etc.<br>Type of vaccination<br>Batch number (if known)	
Vaccination Adverse Reaction Form	First vaccination	Details of ARs thought to be related to SARS-CoV-2 vaccination	As soon as possible after relevant vaccination
	Second vaccination (booster)		
	Third vaccination etc.		
Research Sample Collection Form		Confirmation of collection of research samples in accordance with the protocol	As soon as possible on collection of research samples
Follow-up *		Survival data, COVID-19 infection data, WHO performance status, disease site specific	Adult group: Six months post second vaccination (booster) and 12 months post first vaccine<br>Children and Adolescent group: Six months post last vaccine dose
Death		Date and cause of death	Immediately upon notification of participant's death

CRCTU-PRT-QCD-001, version 1.0

Form	Summary of Data Recorded*	Schedule for Submission to Trial Office
Withdrawal of Consent	Used to notify the Trial Office of the participant's withdrawal from the trial	Immediately upon patient withdrawal
Deviation Form	Details on deviations from the protocol not captured elsewhere on the CRF	Immediately upon discovery of a deviation
Serious Adverse Event	Details of any SAE thought to be related to SARS-CoV-2 vaccination	No later than 24h after becoming aware of the event

* It is anticipated data will collected retrospectively from the participants medical records.

This trial will use an eRDC system to capture the CRF data, the only exception to this will be the registration data which will be captured in an Excel workbook (a paper version of the Registration Form is also available) and the SAE Form which will be completed on paper.

Access to the eRDC system will be granted to site research staff by the Trial Office.

https://crctu.redcap.bham.ac.uk/

The investigator and site staff will ensure all data is promptly entered into the eRDC system in accordance with the trial specific User Manual and CRF Completion Guidelines. The CRF must be completed by the investigator or an authorised member of the site research team (as delegated on the Site Signature and Delegation Log).

Entries on the paper CRF should be made in ballpoint pen, in blue or black ink, and must be legible. Any errors should be crossed out with a single stroke, the correction inserted and the change initialled and dated. If it is not obvious why a change has been made, an explanation should be written next to the change.

Data reported on the CRF should be consistent with the source data or the discrepancies should be explained. If information is not known, this must be clearly indicated on the form. All missing and ambiguous data will be queried. All sections are to be completed before returning.

In all cases it remains the responsibility of the investigator to ensure that the CRF has been completed correctly and that the data are accurate.

The CRF may be amended from time to time by the Trial Office throughout the duration of the trial. Whilst this will not constitute a protocol amendment, new versions of the CRF must be implemented by participating sites immediately on receipt.

9.1 Archiving

It is the responsibility of the Principal Investigator to ensure all essential trial documentation and source records (e.g. signed Informed Consent Forms, Investigator Site Files, participants' hospital notes, etc.) at their site are securely retained for at least 10 years after the end of the trial. Do not destroy any documents without prior approval from the CRCTU Archivist.

10.QUALITY MANAGEMENT

10.1 Site Set-up and Initiation

All sites will be required to sign a model Clinical Trials Agreement (mNCA) prior to participation. In addition, all participating investigators will be asked to sign the necessary agreements e.g. Registration Forms and supply a current *curriculum vitae* (CV) to the Trial Office. Prior to commencing recruitment all sites will undergo a process of initiation. Site initiation meetings will be held on request. Where these are held, key members of the site research team will be invited to attend a teleconference covering aspects of the trial design, protocol procedures, collection and reporting of data and record keeping. Sites will be provided with the documentation for an Investigator Site File containing essential documentation, instructions, and other documentation required for the conduct of the trial. The Trial Office must be informed immediately of any change in the site research team.

10.2 On-site Monitoring

Monitoring will be carried out as required following a risk assessment and as documented in the OCTAVE Quality Management Plan. Additional on-site monitoring visits may be triggered for example by poor CRF return, poor data quality, low SAE reporting rates, excessive number of patient withdrawals or deviations. If a monitoring visit is required the Trial Office will contact the site to arrange a date for the proposed visit and will provide the site with written confirmation. Investigators will allow the OCTAVE trial staff access to source documents as requested.

10.3 Central Monitoring

The Trial Office will be in regular contact with the site research team to check on progress and address any queries that they may have. Trial staff will check incoming CRF for compliance with the protocol, data consistency, missing data and timing. Sites will be sent queries through the REDCap system requesting missing data or clarification of inconsistencies or discrepancies.

Sites may be suspended from further recruitment in the event of serious and persistent non-compliance with the protocol and/or Good Clinical Practice (GCP). Any major problems identified during monitoring may be reported to the Trial Management Group (TMG) and the relevant regulatory bodies. This includes reporting serious breaches of GCP and/or the trial protocol to the REC (see Section 9.5 for further details).

10.4 Audit and Inspection

The investigator will permit trial-related monitoring, audits, ethical review, and regulatory inspection(s) at their site, providing direct access to source data/documents.

Sites are requested to notify the Trial Office of any MHRA inspections.

10.5 Notification of Serious Breaches

In accordance with Regulation 29A of the Medicines for Human Use (Clinical Trials) Regulations 2004 and its amendments the sponsor of the trial is responsible for notifying the licensing authority in writing of any serious breach of:

- The conditions and principles of GCP in connection with that trial or;
- The protocol relating to that trial, within 7 days of becoming aware of that breach

For the purposes of this regulation, a “serious breach” is a breach which is likely to effect to a significant degree:

- The safety or physical or mental integrity of the subjects of the trial; or
- The scientific value of the trial

Sites are therefore requested to notify the Trial Office of a suspected trial-related serious breach of GCP and/or the trial protocol. Where the Trial Office is investigating whether or not a serious breach has occurred sites are also requested to cooperate with the Trial Office in providing sufficient information to report the breach to the MHRA where required and in undertaking any corrective and/or preventive action.

11. END OF TRIAL DEFINITION

The end of trial will be 18 months after the last participants’ last data capture. This will allow sufficient time for the completion of protocol procedures, data collection and data input, and sample analyses. The Trial Office will notify the REC and MHRA that the trial has ended and will provide them with a summary of the trial report within 12 months of the end of trial.

12. STATISTICAL CONSIDERATIONS

12.1 Definition of Outcome Measures

12.1.1 Primary Outcome Measures

The primary outcome measure is defined in Section 2.2.1.

12.1.2 Secondary Outcome Measures

Secondary outcome measures are defined in Section 2.2.2.

CRCTU-PRT-QCD-001, version 1.0

12.2 Analysis of Outcome Measures

Full details of the final analyses will be fully documented in the Statistical Analysis Plan but an outline of the analysis methodologies of the primary and secondary outcome measures are provided here.

OCTAVE is a multicentre, prospective observational trial, examining humoral immunogenicity in multiple prospective cohorts of patients with end stage kidney disease, liver disease, cancer, immune-mediated rheumatic diseases, and transplant recipients. Joint analysis may be held with other disease datasets as the trial evolves.

Three different groups of research samples are being assessed during the trial: Deep Immunophenotyping Group - each disease cohort will include between 100 and 200 adult participants; and the Serology Group – each disease cohort will include between 150 and 850 adult participants; and Serology Plus Group will include up to 160 participants aged 5 - <18 years. The study will recruit up to 3,250 participants in total. Samples are being collected in accordance with the study's schedule (see Schedule of Events).

The primary and secondary outcomes will be assessed using assays and methods proven to give reliable and reproducible results, allowing for direct comparison of participants data, and removing measurement bias.

Vaccination responses will be assessed against a matched control group of vaccinated healthy participants from the health care workers, care homes, and BAME groups for the adult participants and from the COV-Boost trial for the children and adolescent cohort. Case-to-control matching will be performed using variables defined in the statistical analysis plan.

Missing data will be presented and appropriate sensitivity analysis will be considered including per protocol analysis and multiple imputation.

Statistical analyses will be performed using appropriate statistical software (e.g. Stata, Stata Inc, Texas, USA).

12.2.1 Primary Outcome Measures

Continuous measures will be summarised via means, medians, standard deviations, interquartile ranges (IQR) and ranges. Categorical measures will be summarised with number and proportion in each category. To determine the magnitude of the humoral and T cell immunogenicity following vaccination, logistic and linear regression will investigate the relationship of response in disease groups in comparison to the healthy control group (if appropriate parametric or more flexible models may be considered e.g. mixed effect models). Comparisons to look at differences in IgG response from vaccination and/or infection across disease groups at specific time points will be carried out using two-sample t-tests. Appropriate data plots will be produced where applicable. Response comparisons will be made comparing disease-to-disease and disease-to-control groups (where appropriate healthy control data is available). Correlation of responses assessed using the different assays will be performed using Pearson Correlation or Spearman's Rank where appropriate.

Once data collection of the Oxford Immunotec modified T-SPOT Discovery SARS-CoV-2 assay has been completed, an interim assessment of this data will be performed. A comparison of results with a

control group is not planned to be performed and included in the interim analyses. This interim analysis is to be completed within 1 year of OCTAVE being opened.

12.2.2 Secondary Outcome Measures

Appropriate summary statistics will be produced (continuous measures via means, medians, standard deviations, IQRs and ranges, categorical measures with number and proportion in each category). Data will be analysed using appropriate regression modelling (parametric and more flexible models may be considered), giving estimates of response at specific time points. Response estimates (absolute changes) at specific time points will be compared between groups (disease-to-disease and disease-to-control) using two-sample t-tests. Contingency tables, Fisher's Exact and Chi-Square testing will be used to investigate categorical data where applicable. Appropriate data plots will be produced where applicable.

12.3 Planned Subgroup Analyses

These analyses are not powered and as such are for exploratory information. Statistical analyses methods given in Section 12.2.2 will be utilised where appropriate.

- Vaccination response in disease-specific subgroups and comparison to control groups
- Vaccine specific response comparisons in disease and control groups
- The effect of disease-specific medications on vaccination response

12.4 Exploratory Analyses

For brevity, the exploratory analyses will utilise the methods to analyse data as described in Section 12.2.2 where appropriate.

12.5 Sample Size Justification

OCTAVE is an observational trial and as such no formal sample size calculations were feasible. However, the numbers of participants for the Deep Immunophenotyping, Serology and Serology Plus Groups have been selected based on the availability of potential participants who have the underlying diseases in the investigated populations to recruit from within the specified time and funding. It is felt that the sample sizes are large enough to adequately investigate the trial's objectives.

13. TRIAL ORGANISATIONAL STRUCTURE

This is a collaborative trial being conducted by the University of Glasgow, University of Birmingham, University of Oxford and Imperial College London. Additional sites may also choose to take part.

13.1 Sponsor

The trial is being sponsored by the University of Birmingham.

CRCTU-PRT-QCD-001, version 1.0

13.2 Coordinating Centre

The trial is being conducted under the auspices of the CRCTU, University of Birmingham according to their local procedures.

13.3 Access Committee

An Access Committee has been established to ensure the appropriate governance of the research samples. Samples will be made available to academic researchers for ethically approved studies, subject to the approval of the Access Committee.

13.4 Trial Management Group

The Chief Investigator, cohort leads, co-investigators, trial statisticians, Patient and Public Involvement and Engagement (PPIE) representatives, Trial Management Team Leader and Trial Coordinator will form the TMG. The TMG will be responsible for the day-to-day conduct of the trial. They will be responsible for the clinical set-up, promotion, on-going management of the trial, the interpretation of the results and preparation and presentation of relevant publications.

The TMG will meet formally (usually virtually) every month during the recruitment phase of the trial. Thereafter the formal TMG meetings may be replaced by a regular progress report.

13.5 Independent Oversight Committees

No independent oversight committees have been established for this low risk cohort trial.

13.6 Finance

This is an investigator-initiated and investigator-led trial funded by the Medical Research Council**.**

The collaborating institutions will receive payment from the funder to pay for participant recruitment. Additional NHS sites will receive payments to cover any NHS Research costs.

Participants will be able to claim travel expenses for extra clinic visits but no other payments will be made for taking part in the trial.

14. ETHICAL CONSIDERATIONS

The trial will be performed in accordance with the recommendations guiding physicians in biomedical research involving human subjects, adopted by the 18th World Medical Association General Assembly, Helsinki, Finland, June 1964, amended at the 48th World Medical Association General Assembly, Somerset West, Republic of South Africa, October 1996 (Appendix 4).

The trial will be conducted in accordance with the UK Policy Framework for Health and Social Care Research 2017, the applicable UK Statutory Instruments, which include the Medicines for Human Use Clinical Trials 2004 and subsequent amendments; the General Data Protection Regulation 2018; and Data Protection Act 2018; and Human Tissue Act 2004 or Human Tissue (Scotland) Act 2006 and GCP.

This trial will be carried out under a Clinical Trial Authorisation in accordance with the Medicines for Human Use Clinical Trials regulations. The protocol will be submitted to and approved by the REC and Health Research Authority prior to circulation.

Before any patients are enrolled into the trial, the Principal Investigator at each site is required to obtain formal confirmation of capacity and capability from their local R&D Department and provide evidence of this to the Trial Office. Sites will not be permitted to enrol patients until this has been obtained.

It is the responsibility of the Principal Investigator to ensure that all subsequent amendments gain the necessary local approval. This does not affect the individual clinicians' responsibility to take immediate action if thought necessary to protect the health and interest of individual patients.

15.CONFIDENTIALITY AND DATA PROTECTION

The University of Birmingham is the Data Controller for this trial. Personal data recorded on all documents will be regarded as strictly confidential and will be handled and stored in accordance with the General Data Protection Regulation 2018 and the Data Protection Act 2018. Data will be processed under Article 6 (i) (performance of a task carried out in the public interest) and Article 9 (j) (necessary for archiving purposes in the public interest, scientific or historical research purposes or statistical purposes in accordance with Article 89(1)). Information about how information is handled can be found in the CRCTU and University of Birmingham's privacy policies (https://www.birmingham.ac.uk/research/activity/mds/trials/crctu/crctu-privacy-notice.aspx).

The participant's initials, date of birth, NHS/CHI Number and hospital number will be collected at trial entry to aid in identification and matching with other data sources where appropriate for the purposes of further analysis. Patients will be identified using only their unique trial number and initials on the CRF and correspondence between the Trial Office and the participating site.

The investigator must maintain documents not for submission to the Trial Office in strict confidence. In the case of specific issues and/or queries from the regulatory authorities, it will be necessary to have access to the complete trial records, provided that participant confidentiality is protected.

Representatives of the CRCTU may be required to have access to patient's notes for quality assurance purposes but patients should be reassured that their confidentiality will be respected at all times.

The Trial Office will maintain the confidentiality of all participants' data and will not disclose information by which participant may be identified to any third party other than those directly involved in the treatment of the participant and organisations for which the participant has given consent for data transfer. Anonymised participant level data and research samples may be shared in accordance with the CRCTU Data Sharing Policy (see Section 18 for further details).

16.INSURANCE AND INDEMNITY

The University of Birmingham has in place indemnity which provides cover to the University for harm which comes about through the University's, or its staff's, negligence in relation to the design or management of the trial and may alternatively, and at the University's discretion provide cover for non-negligent harm to participants.

With respect to the conduct of the trial at site and other clinical care of the participant, responsibility remains with the NHS organisation responsible for the clinical site and is therefore indemnified through the NHS Litigation Authority.

The University of Birmingham is independent of any pharmaceutical company, and as such it is not covered by the Association of the British Pharmaceutical Industry (ABPI) guidelines for participant compensation.

17.PUBLICATION POLICY

Results of this trial will be submitted for publication in a peer reviewed journals. The manuscripts will be prepared by the TMG and authorship will be on behalf of the collaborative group.

Any secondary publications and presentations prepared by investigators must be reviewed by the TMG. Manuscripts must be submitted to the TMG in a timely fashion and in advance of being submitted for publication, to allow time for review and resolution of any outstanding issues. Authors must acknowledge that the trial was performed with the support of University of Birmingham and all funding bodies must be appropriately acknowledged in accordance with the funder's terms and conditions. Intellectual property rights will be addressed in the agreements between sponsor, collaborators and the sites.

The results of the trial will be made available on ISRCTN and provided to participants in the form of a lay summary on the trial website.

18.DATA SHARING

The CRCTU is committed to responsible and controlled sharing of anonymised clinical trial data with the wider research community to maximise potential patient benefit while protecting the privacy and confidentiality of trial participants. Data anonymised in compliance with the Information Commissioners Office requirements, using a procedure based on guidelines from the MRC Methodology Hubs, will be available for sharing with researchers outside of the trials team within 6 months of the primary publication.

More detailed information on the CRCTU's Data Sharing Policy and the mechanism for obtaining data can be found on the CRCTU website:
https://www.birmingham.ac.uk/research/activity/mds/trials/crctu/index.aspx.

19.REFERENCE LIST

1. Vijenthira, A., et al., *Outcomes of patients with hematologic malignancies and COVID-19: A systematic review and meta-analysis of 3377 patients.* Blood, 2020.
2. Chari, A., et al., *Clinical features associated with COVID-19 outcome in multiple myeloma: first results from the International Myeloma Society data set.* Blood, 2020. **136**(26): p. 3033–3040.
3. Cook, G., et al., *Real-world assessment of the clinical impact of symptomatic infection with severe acute respiratory syndrome coronavirus (COVID-19 disease) in patients with multiple myeloma receiving systemic anti-cancer therapy.* British journal of haematology, 2020. **190**(2): p. e83-e86.
4. Lee, L.Y.W., et al., *COVID-19 mortality in patients with cancer on chemotherapy or other anticancer treatments: a prospective cohort study.* The Lancet, 2020. **395**(10241): p. 1919-1926.
5. Lee, L.Y.W., et al., *COVID-19 prevalence and mortality in patients with cancer and the effect of primary tumour subtype and patient demographics: a prospective cohort study.* The Lancet Oncology, 2020. **21**(10): p. 1309-1316.
6. Bitterman, R., et al., *Influenza vaccines in immunosuppressed adults with cancer.* Cochrane Database Syst Rev, 2018. **2**(2): p. Cd008983.
7. UK, C.R.; Available from: https://www.cancerresearchuk.org/health-professional/cancer-statistics/statistics-by-cancer-type/breast-cancer.
8. Patel, A.J., et al., *Characterising the impact of pneumonia on outcome in non-small cell lung cancer: identifying preventative strategies.* Journal of Thoracic Disease, 2020. **12**(5): p. 2236-2246.
9. Favaloro, J., et al., *Myeloid derived suppressor cells are numerically, functionally and phenotypically different in patients with multiple myeloma.* Leuk Lymphoma, 2014. **55**(12): p. 2893-900.
10. Pittari, G., et al., *Restoring Natural Killer Cell Immunity against Multiple Myeloma in the Era of New Drugs.* Frontiers in immunology, 2017. **8**: p. 1444-1444.
11. Cohen, A.D., et al., *How to Train Your T Cells: Overcoming Immune Dysfunction in Multiple Myeloma.* Clinical Cancer Research, 2020. **26**(7): p. 1541-1554.
12. Leblay, N., et al., *Deregulation of Adaptive T Cell Immunity in Multiple Myeloma: Insights Into Mechanisms and Therapeutic Opportunities.* Frontiers in Oncology, 2020. **10**(636).
13. D'Souza, L. and D. Bhattacharya, *Plasma cells: You are what you eat.* Immunological Reviews, 2019. **288**(1): p. 161-177.
14. Cook, G., et al., *Defining the vulnerable patient with myeloma—a frailty position paper of the European Myeloma Network.* Leukemia, 2020. **34**(9): p. 2285-2294.
15. Blimark, C., et al., *Multiple myeloma and infections: a population-based study on 9253 multiple myeloma patients.* Haematologica, 2015. **100**(1): p. 107-13.
16. Augustson, B.M., et al., *Early mortality after diagnosis of multiple myeloma: analysis of patients entered onto the United kingdom Medical Research Council trials between 1980 and 2002--Medical Research Council Adult Leukaemia Working Party.* J Clin Oncol, 2005. **23**(36): p. 9219-26.
17. Martínez-López, J., et al., *Multiple myeloma and SARS-CoV-2 infection: clinical characteristics and prognostic factors of inpatient mortality.* Blood Cancer Journal, 2020. **10**(10): p. 103.
18. Loke, J., et al., *The role of allogeneic stem cell transplantation in the management of acute myeloid leukaemia: a triumph of hope and experience.* British Journal of Haematology, 2020. **188**(1): p. 129-146.
19. DiNardo, C.D., et al., *Azacitidine and Venetoclax in Previously Untreated Acute Myeloid Leukemia.* New England Journal of Medicine, 2020. **383**(7): p. 617-629.
20. García-Suárez, J., et al., *Impact of hematologic malignancy and type of cancer therapy on COVID-19 severity and mortality: lessons from a large population-based registry study.* Journal of Hematology & Oncology, 2020. **13**(1): p. 133.
21. Passamonti, F., et al., *Clinical characteristics and risk factors associated with COVID-19 severity in patients with haematological malignancies in Italy: a retrospective, multicentre, cohort study.* The Lancet Haematology, 2020. **7**(10): p. e737-e745.
22. Frazier, O.H., et al., *Clinical experience with the Hemopump.* ASAIO Trans, 1989. **35**(3): p. 604-6.
23. Li, X., et al., *Comparative risk of serious infections among real-world users of biologics for psoriasis or psoriatic arthritis.* Ann Rheum Dis, 2020. **79**(2): p. 285-291.
24. Sarica, S.H., et al., *Characterizing infection in anti-neutrophil cytoplasmic antibody-associated vasculitis: results from a longitudinal, matched-cohort data linkage study.* Rheumatology (Oxford), 2020. **59**(10): p. 3014-3022.

25. George, M.D., et al., *Risk for Serious Infection With Low-Dose Glucocorticoids in Patients With Rheumatoid Arthritis : A Cohort Study.* Ann Intern Med, 2020. **173**(11): p. 870-878.
26. contributors, F.R.S.S.S.C.I.c.a., *Severity of COVID-19 and survival in patients with rheumatic and inflammatory diseases: data from the French RMD COVID-19 cohort of 694 patients.* Ann Rheum Dis, 2020.
27. Gianfrancesco, M., et al., *Characteristics associated with hospitalisation for COVID-19 in people with rheumatic disease: data from the COVID-19 Global Rheumatology Alliance physician-reported registry.* Ann Rheum Dis, 2020. **79**(7): p. 859-866.
28. Williamson, E.J., et al., *Factors associated with COVID-19-related death using OpenSAFELY.* Nature, 2020. **584**(7821): p. 430-436.
29. Subesinghe, S., et al., *A Systematic Review and Metaanalysis of Antirheumatic Drugs and Vaccine Immunogenicity in Rheumatoid Arthritis.* J Rheumatol, 2018. **45**(6): p. 733-744.
30. Park, J.K., et al., *Impact of temporary methotrexate discontinuation for 2 weeks on immunogenicity of seasonal influenza vaccination in patients with rheumatoid arthritis: a randomised clinical trial.* Ann Rheum Dis, 2018. **77**(6): p. 898-904.
31. van Assen, S., et al., *Humoral responses after influenza vaccination are severely reduced in patients with rheumatoid arthritis treated with rituximab.* Arthritis Rheum, 2010. **62**(1): p. 75-81.
32. Baker, D., et al., *COVID-19 vaccine-readiness for anti-CD20-depleting therapy in autoimmune diseases.* Clin Exp Immunol, 2020. **202**(2): p. 149-161.
33. Janus, N., et al., *Vaccination and chronic kidney disease.* Nephrol Dial Transplant, 2008. **23**(3): p. 800-7.
34. Docherty, A., et al., *Features of 16,749 hospitalised UK patients with COVID-19 using the ISARIC WHO Clinical Characterisation Protocol.* medRxiv, 2020: p. 2020.04.23.20076042.
35. Corbett, R.W., et al., *Epidemiology of COVID-19 in an Urban Dialysis Center.* Journal of the American Society of Nephrology, 2020. **31**(8): p. 1815-1823.
36. Ng, J.H., et al., *Outcomes of patients with end-stage kidney disease hospitalized with COVID-19.* Kidney International, 2020. **98**(6): p. 1530-1539.
37. Valeri, A.M., et al., *Presentation and Outcomes of Patients with ESKD and COVID-19.* J Am Soc Nephrol, 2020. **31**(7): p. 1409-1415.
38. Ravanan, R., et al., *SARS-CoV-2 infection and early mortality of wait-listed and solid organ transplant recipients in England: a national cohort study.* Am J Transplant, 2020.
39. Girndt, M., et al., *Impaired cellular immune function in patients with end-stage renal failure.* Nephrology Dialysis Transplantation, 1999. **14**(12): p. 2807-2810.
40. Sarnak, M.J. and B.L. Jaber, *Mortality caused by sepsis in patients with end-stage renal disease compared with the general population.* Kidney Int, 2000. **58**(4): p. 1758-64.
41. Alexiewicz, J.M., et al., *Impaired phagocytosis in dialysis patients: studies on mechanisms.* Am J Nephrol, 1991. **11**(2): p. 102-11.
42. Betjes, M.G., *Immune cell dysfunction and inflammation in end-stage renal disease.* Nat Rev Nephrol, 2013. **9**(5): p. 255-65.
43. Prasad, N., et al., *Respiratory Syncytial Virus–Associated Hospitalizations Among Adults With Chronic Medical Conditions.* Clinical Infectious Diseases, 2020.
44. Usvyat, L.A., et al., *Seasonal variations in mortality, clinical, and laboratory parameters in hemodialysis patients: a 5-year cohort study.* Clin J Am Soc Nephrol, 2012. **7**(1): p. 108-15.
45. Corbett, R.W., et al., *Epidemiology of COVID-19 in an Urban Dialysis Center.* J Am Soc Nephrol, 2020. **31**(8): p. 1815-1823.
46. Albillos, A., M. Lario, and M. Álvarez-Mon, *Cirrhosis-associated immune dysfunction: Distinctive features and clinical relevance.* Journal of Hepatology, 2014. **61**(6): p. 1385-1396.
47. Noor, M.T. and P. Manoria, *Immune Dysfunction in Cirrhosis.* Journal of clinical and translational hepatology, 2017. **5**(1): p. 50-58.
48. Marjot, T., et al., *Outcomes following SARS-CoV-2 infection in patients with chronic liver disease: An international registry study.* Journal of Hepatology.
49. Webb, G.J., et al., *Outcomes following SARS-CoV-2 infection in liver transplant recipients: an international registry study.* Lancet Gastroenterol Hepatol, 2020. **5**(11): p. 1008-1016.
50. Kanellopoulos, A., et al., *COVID-19 in bone marrow transplant recipients: reflecting on a single centre experience.* Br J Haematol, 2020. **190**(2): p. e67-e70.
51. Malard, F., et al., *COVID-19 outcomes in patients with hematologic disease.* Bone Marrow Transplant, 2020. **55**(11): p. 2180-2184.

52. Gratwohl, A., et al., *Cause of death after allogeneic haematopoietic stem cell transplantation (HSCT) in early leukaemias: an EBMT analysis of lethal infectious complications and changes over calendar time.* Bone Marrow Transplant, 2005. **36**(9): p. 757-69.
53. Haining, W.N., et al., *Measuring T cell immunity to influenza vaccination in children after haemopoietic stem cell transplantation.* British journal of haematology, 2004. **127**(3): p. 322-325.
54. Cordonnier, C., et al., *Randomized study of early versus late immunization with pneumococcal conjugate vaccine after allogeneic stem cell transplantation.* Clin Infect Dis, 2009. **48**(10): p. 1392-401.
55. Mombelli, M., et al., *Immunogenicity and safety of double versus standard dose of the seasonal influenza vaccine in solid-organ transplant recipients: A randomized controlled trial.* Vaccine, 2018. **36**(41): p. 6163-6169.
56. Boyarsky, B.J., et al., *Antibody Response to 2-Dose SARS-CoV-2 mRNA Vaccine Series in Solid Organ Transplant Recipients.* JAMA, 2021. **325**(21): p. 2204-2206.
57. Heijstek, M.W., et al., *EULAR recommendations for vaccination in paediatric patients with rheumatic diseases.* Annals of the Rheumatic Diseases, 2011. **70**(10): p. 1704-1712.
58. Maritsi, D.N., et al., *Risk Factors Associated With Accelerated Rubella IgG Antibody Loss in Previously Vaccinated, Treatment-Naive Patients With Juvenile Systemic Lupus Erythematosus: A Prospective Study.* Arthritis Rheumatol, 2019. **71**(6): p. 1022-1023.
59. Maritsi, D.N., et al., *Decreased antibodies against rubella in previously vaccinated treatment-naïve childhood systemic lupus erythematous patients: a prospective case-control study.* Scand J Rheumatol, 2019. **48**(1): p. 74-76.
60. Maritsi, D.N., et al., *Decreased antibodies against hepatitis A in previously vaccinated treatment naïve juvenile SLE patients: a prospective case control study.* Clin Exp Rheumatol, 2017. **35**(3): p. 544-545.
61. Service, N.C.R.a.A. *Children, teenagers and young adults UK cancer statistics report 2021*. 2021 [cited 2021 09-Dec-2021]; Available from: http://ncin.org.uk/cancer_type_and_topic_specific_work/cancer_type_specific_work/cancer_in_children_teenagers_and_young_adults/.
62. *Children, teenagers and young adults UK cancer statistics report 2021*.
63. Erik A H Loeffen, R.R.G.K., Joren Boerhof, E A M Lieke Feijen, Johannes H M Merks, Ardine M J Reedijk, Jan A Lieverst, Rob Pieters, H Marike Boezen, Leontien C M Kremer, Wim J E Tissing, *Treatment-related mortality in children with cancer: Prevalence and risk factors.* Eur J Cancer, 2019. **121**: p. 113-122.
64. Anne-Sophie E Darlington, J.E.M., Richard Wagland, Samantha C Sodergren, David Culliford, Ashley Gamble, Bob Phillips, *COVID-19 and children with cancer: Parents' experiences, anxieties and support needs.* Pediatr Blood Cancer, 2021. **68**(2): p. e28790.
65. Gerard C. Millen, R.A., Jean-Baptiste Cazier, Helen Curley, Richard G. Feltbower, Ashley Gamble, Adam W. Glaser, Richard G. Grundy, Lennard Y. W. Lee, Martin G. McCabe, Robert S. Phillips, Charles A. Stiller, Csilla Várnai & Pamela R. Kearns, *Severity of COVID-19 in children with cancer: Report from the United Kingdom Paediatric Coronavirus Cancer Monitoring Project.* British Journal of cancer, 2021. **124**: p. 754-759.
66. G C Millen, R.A., JB Cazier, H Curley, R Feltbower, A Gamble, A Glaser, R G Grundy, L Kirton, L Y W Lee, M G McCabe, C Palles, B Phillips, C A Stiller, C Varnai, P Kearns, *COVID-19 in children with haematological malignancies.* Arch Dis Child, 2021.

APPENDIX 1 - DEFINITION OF ADVERSE EVENTS

12.1 Adverse Event (AE)

Any untoward medical occurrence in a patient or clinical trial subject administered a medicinal product and which does not necessarily have a causal relationship with this treatment.

Comment:

An AE can therefore be any unfavourable and unintended sign (including abnormal laboratory findings), symptom or disease temporally associated with the use of an IMP whether or not related to the IMP.

12.2 Adverse Reaction (AR)

All untoward and unintended responses to an IMP related to any dose administered.

Comment:

An AE judged by either the reporting investigator or sponsor as having causal relationship to the IMP qualifies as an AR. The expression reasonable causal relationship means to convey in general that there is evidence or argument to suggest a causal relationship.

12.3 Serious Adverse Event (SAE)

Any untoward medical occurrence or effect that at any dose:

- Results in death
- Is life threatening*
- Requires hospitalisation** or prolongation of existing inpatients' hospitalisation
- Results in persistent or significant disability or incapacity
- Is a congenital anomaly/birth defect
- Or is otherwise considered medically significant by the investigator***

Comments:

The term severe is often used to describe the intensity (severity) of a specific event. This is not the same as serious, which is based on patients/event outcome or action criteria.

* Life threatening in the definition of an SAE refers to an event in which the participant was at risk of death at the time of the event; it does not refer to an event that hypothetically might have caused death if it were more severe.

**Hospitalisation is defined as an unplanned, formal inpatient admission, even if the hospitalisation is a precautionary measure for continued observation. Thus hospitalisation for protocol treatment (e.g. line insertion), elective procedures (unless brought forward because of worsening symptoms) or for social reasons (e.g. respite care) are not regarded as an SAE.

*** Medical judgment should be exercised in deciding whether an AE is serious in other situations. Important AEs that are not immediately life threatening or do not result in death or hospitalisation but may jeopardise the participant or may require intervention to prevent one of the other outcomes listed in the definition above, should be considered serious.

12.4 Serious Adverse Reaction (SAR)

An Adverse Reaction which also meets the definition of a Serious Adverse Event.

12.5 Suspected Unexpected Serious Adverse Reaction (SUSAR)

A SAR that is unexpected i.e. the nature, or severity of the event is not consistent with the applicable product information.

A SUSAR should meet the definition of an AR, UAR and SAR.

12.6 Unexpected Adverse Reaction (UAR)

An AR, the nature or severity of which is not consistent with the Reference Safety Information.

When the outcome of an AR is not consistent with the Reference Safety Information, the AR should be considered unexpected.

APPENDIX 2 - COMMON TOXICITY CRITERIA GRADINGS

Toxicities will be recorded according to the Common Terminology Criteria for Adverse Events (CTCAE), version 4.03. The full CTCAE document is available on the National Cancer Institute (NCI) website, the following address was correct when this version of the protocol was approved: https://www.eortc.be/services/doc/ctc/ctcae_4.03_2010-06-14_quickreference_5x7.pdf

CRCTU-PRT-QCD-001, version 1.0

APPENDIX 3 – WORLD HEALTH ORGANISATION (WHO) PERFORMANCE STATUS

Grade	WHO Performance Status
0	Fully active, able to carry on all pre-disease performance without restriction
1	Restricted in physically strenuous activity but ambulatory and able to carry out work of a light or sedentary nature, e.g. light house work, office work
2	Ambulatory and capable of all self-care but unable to carry out any work activities. Up and about more than 50% of waking hours
3	Capable of only limited self-care, confined to bed or chair more than 50% of waking hours
4	Completely disabled. Cannot carry on any self-care. Totally confined to bed or chair
5	Dead

APPENDIX 4 - WMA DECLARATION OF HELSINKI

Recommendations guiding physicians in biomedical research involving human patients

Adopted by the 18th World Medical Assembly Helsinki, Finland, June 1964
and amended by the
29th World Medical Assembly, Tokyo, Japan, October 1975
35th World Medical Assembly, Venice, Italy, October 1983
41st World Medical Assembly, Hong Kong, September 1989
48th General Assembly, Somerset West, Republic of South Africa, October 1996

Introduction

It is the mission of the physician to safeguard the health of the people. His or her knowledge and conscience are dedicated to the fulfilment of this mission.

The Declaration of Geneva of the World Medical Association binds the physician with the words, "The Health of my patient will be my first consideration," and the International Code of Medical Ethics declares that, "A physician shall act only in the patient's interest when providing medical care which might have the effect of weakening the physical and mental condition of the patient."

The purpose of biomedical research involving human patients must be to improve diagnostic, therapeutic and prophylactic procedures and the understanding of the aetiology and pathogenesis of disease.

In current medical practice most diagnostic, therapeutic or prophylactic procedures involve hazards. This applies especially to biomedical research.

Medical progress is based on research which ultimately must rest in part on experimentation involving human patients.

In the field of biomedical research a fundamental distinction must be recognized between medical research in which the aim is essentially diagnostic or therapeutic for a patient, and medical research, the essential object of which is purely scientific and without implying direct diagnostic or therapeutic value to the person subjected to the research.

Special caution must be exercised in the conduct of research which may affect the environment, and the welfare of animals used for research must be respected.

Because it is essential that the results of laboratory experiments be applied to human beings to further scientific knowledge and to help suffering humanity, the World Medical Association has prepared the following recommendations as a guide to every physician in biomedical research involving human patients. They should be kept under review in the future. It must be stressed that the standards as drafted are only a guide to physicians all over the world. Physicians are not relieved from criminal, civil and ethical responsibilities under the laws of their own countries.

I. Basic principles

a. Biomedical research involving human patients must conform to generally accepted scientific principles and should be based on adequately performed laboratory and animal experimentation and on a thorough knowledge of the scientific literature.
b. The design and performance of each experimental procedure involving human patients should be clearly formulated in an experimental protocol which should be transmitted for consideration, comment and guidance to a specially appointed committee independent of the investigator and the sponsor provided that this independent committee is in conformity with the laws and regulations of the country in which the research experiment is performed.
c. Biomedical research involving human patients should be conducted only by scientifically qualified persons and under the supervision of a clinically competent medical person. The responsibility for the human patient must always rest with a medically qualified person and never rest on the patient of the research, even though the patient has given his or her consent.
d. Biomedical research involving human patients cannot legitimately be carried out unless the importance of the objective is in proportion to the inherent risk to the patient.
e. Every biomedical research project involving human patients should be preceded by careful assessment of predictable risks in comparison with foreseeable benefits to the patient or to others. Concern for the interests of the patient must always prevail over the interests of science and society.
f. The right of the research patient to safeguard his or her integrity must always be respected. Every precaution should be taken to respect the privacy of the patient and to minimize the impact of the study on the patient's physical and mental integrity and on the personality of the patient.
g. Physicians should abstain from engaging in research projects involving human patients unless they are satisfied that the hazards involved are believed to be predictable. Physicians should cease any investigation if the hazards are found to outweigh the potential benefits.
h. In publication of the results of his or her research, the physician is obliged to preserve the accuracy of the results. Reports of experimentation not in accordance with the principles laid down in this Declaration should not be accepted for publication.
i. In any research on human beings, each potential patient must be adequately informed of the aims, methods, anticipated benefits and potential hazards of the study and the discomfort it may entail. He or she should be informed that he or she is at liberty to abstain from participation in the study and that he or she is free to withdraw his or her consent to participation at any time. The physician should then obtain the patient's freely-given informed consent, preferably in writing.
j. When obtaining informed consent for the research project the physician should be particularly cautious if the patient is in a dependent relationship to him or her or may consent under duress. In that case the informed consent should be obtained by a physician who is not engaged in the investigation and who is completely independent of this official relationship.
k. In case of legal incompetence, informed consent should be obtained from the legal guardian in accordance with national legislation. Where physical or mental incapacity makes it impossible to obtain informed consent, or when the patient is a minor, permission from the

responsible relative replaces that of the patient in accordance with national legislation. Whenever the minor child is in fact able to give a consent, the minor's consent must be obtained in addition to the consent of the minor's legal guardian.

l. The research protocol should always contain a statement of the ethical considerations involved and should indicate that the principles enunciated in the present Declaration are complied with.

II. Medical research combined with professional care (Clinical Research)

1. In the treatment of the sick person, the physician must be free to use a new diagnostic and therapeutic measure, if in his or her judgement it offers hope of saving life, re-establishing health or alleviating suffering.
2. The potential benefits, hazards and discomfort of a new method should be weighed against the advantages of the best current diagnostic and therapeutic methods.
3. In any medical study, every patient - including those of a control group, if any - should be assured of the best proven diagnostic and therapeutic method. This does not exclude the use of inert placebo in studies where no proven diagnostic or therapeutic method exists.
4. The refusal of the patient to participate in a study must never interfere with the physician-patient relationship.
5. If the physician considers it essential not to obtain informed consent, the specific reasons for this proposal should be stated in the experimental protocol for transmission to the independent committee (I, 2).
6. The physician can combine medical research with professional care, the objective being the acquisition of new medical knowledge, only to the extent that medical research is justified by its potential diagnostic or therapeutic value for the patient.

III. Non-therapeutic biomedical research involving human Patients (Non-Clinical Biomedical Research)

1. In the purely scientific application of medical research carried out on a human being, it is the duty of the physician to remain the protector of the life and health of that person on whom biomedical research is being carried out.
2. The patient should be volunteers - either healthy persons or patients for whom the experimental design is not related to the patient's illness.
3. The investigator or the investigating team should discontinue the research if in his/her or their judgement it may, if continued, be harmful to the individual.
4. In research on man, the interest of science and society should never take precedence over considerations related to the wellbeing of the patient.

CRCTU-PRT-QCD-001, version 1.0

TRIAL OFFICE

Cancer Research UK Clinical Trials Unit
Institute of Cancer and Genomic Sciences
University of Birmingham
Edgbaston
Birmingham B15 2TT
☎ 0121 414 3100 🖷 0121 414 2230
✉ OCTAVE@trials.bham.ac.uk
www.birmingham.ac.uk/octave

Trial Database

https://crctu.redcap.bham.ac.uk/

Serious Adverse Event Reporting

Reg@trials.bham.ac.uk
CC OCTAVE@trials.bham.ac.uk
Include "OCTAVE SAE" in the subject line

CRCTU-PRT-QCD-001, version 1.0
