# Supplementary material for: SARS-CoV-2-specific immune responses and clinical outcomes after COVID-19 vaccination in patients with immune-suppressive disease
Source: Nat Med. 2023 Jul 6;29(7):1760–74. doi: 10.1038/s41591-023-02414-4 (PMC10353927; doi:10.1038/s41591-023-02414-4)
Supplement: Supplementary file 2 — Reporting Summary [file 41591_2023_2414_MOESM2_ESM.pdf]

nature portfolio

Corresponding author(s): Iain McInnes

Last updated by author(s): 15/05/2023

Reporting Summary

Nature Portfolio wishes to improve the reproducibility of the work that we publish. This form provides structure for consistency and transparency in reporting. For further information on Nature Portfolio policies, see our Editorial Policies and the Editorial Policy Checklist.

Statistics

For all statistical analyses, confirm that the following items are present in the figure legend, table legend, main text, or Methods section.

n/a	Confirmed	
☐	☒	The exact sample size (*n*) for each experimental group/condition, given as a discrete number and unit of measurement
☐	☒	A statement on whether measurements were taken from distinct samples or whether the same sample was measured repeatedly
☐	☒	The statistical test(s) used AND whether they are one- or two-sided<br>*Only common tests should be described solely by name; describe more complex techniques in the Methods section.*
☐	☒	A description of all covariates tested
☐	☒	A description of any assumptions or corrections, such as tests of normality and adjustment for multiple comparisons
☐	☒	A full description of the statistical parameters including central tendency (e.g. means) or other basic estimates (e.g. regression coefficient) AND variation (e.g. standard deviation) or associated estimates of uncertainty (e.g. confidence intervals)
☐	☒	For null hypothesis testing, the test statistic (e.g. *F*, *t*, *r*) with confidence intervals, effect sizes, degrees of freedom and *P* value noted<br>*Give P values as exact values whenever suitable.*
☒	☐	For Bayesian analysis, information on the choice of priors and Markov chain Monte Carlo settings
☐	☒	For hierarchical and complex designs, identification of the appropriate level for tests and full reporting of outcomes
☐	☒	Estimates of effect sizes (e.g. Cohen's *d*, Pearson's *r*), indicating how they were calculated

Our web collection on statistics for biologists contains articles on many of the points above.

Software and code

Policy information about availability of computer code

Data collection	Data were collected and stored using a REDCap v12.3.1 ©2022 Vanderbilt University.
Data analysis	Data analyses were performed using Stata v17.0, StataCorp, Texas USA; GraphPad Prism (v9.4.); R (v4.2.1) with RStudio 2022.02.3.

For manuscripts utilizing custom algorithms or software that are central to the research but not yet described in published literature, software must be made available to editors and reviewers. We strongly encourage code deposition in a community repository (e.g. GitHub). See the Nature Portfolio guidelines for submitting code & software for further information.

Data

Policy information about availability of data

All manuscripts must include a data availability statement. This statement should provide the following information, where applicable:

- Accession codes, unique identifiers, or web links for publicly available datasets
- A description of any restrictions on data availability
- For clinical datasets or third party data, please ensure that the statement adheres to our policy

Participant data and the associated supporting documentation will be available within 6 months after the publication of this manuscript. Details of our data request process is available on the CRCTU website. Only scientifically sound proposals from appropriately qualified research groups will be considered for data sharing. The decision to release data will be made by the CRCTU Director's Committee, who will consider the scientific validity of the request, the qualifications and resources of the research group, the views of the Chief Investigator and the trial steering committee, consent arrangements, the practicality of anonymising the requested data

and contractual obligations. A data sharing agreement will cover the terms and conditions of the release of trial data and will include publication requirements, authorship and acknowledgements and obligations for the responsible use of data. An anonymised encrypted dataset will be transferred directly using a secure method and in accordance with the University of Birmingham's IT guidance on encryption of data sets. Information on data requests, including a contact address and expected time frame of requests can be found at the following link: https://www.birmingham.ac.uk/research/crctu/data-sharing-policy.aspx

Human research participants

Policy information about studies involving human research participants and Sex and Gender in Research.

Reporting on sex and gender	Participants of male and female sex were included in this study and their demographics are included in Table 1. Sex was included as a variable in multivariable logistic models.
Population characteristics	The covariate-relevant population characteristics collected in all participants are included in Table 1 and include: Age, sex, ethnicity, BMI, Smoking status, diabetes diagnosis and disease phenotype.
Recruitment	Written informed consent was obtained from all the participants. Adult patients in clinically vulnerable groups were recruited between 19 February 2021 and 01 October 2021 based on the following eligibility criteria:<br>- Are eligible for vaccination by one of the SARS-CoV-2 vaccines approved by the MHRA administered in accordance with national guidelines.<br>- Have not received their second dose of the vaccine for the "deep immunophenotyping group", or have not passed the day 28 post second vaccine dose timepoint (21-84 days after second vaccination) for the "serology group".<br>- Have an anticipated life span of 6 months or greater.<br>- Have a diagnosis belonging to one of the following disease groups: Solid cancer (SC); Haematological malignancy (HM); Rheumatic Inflammatory Conditions (including ANCA Associated Vasculitis on rituximab (AAV) and Inflammatory Arthritis (IA)), Chronic renal disease (including end-stage kidney disease and kidney transplantation (K-Tr)), Chronic liver disease (including liver cirrhosis (L-Cir), liver disease on immunosuppressive therapy (L-AI) or liver transplantation (L-Tr)), Inflammatory Bowel Disease on immunosuppressive therapy (IBD; Crohn's disease (CD), ulcerative colitis (UC) and undefined IBD-U) and Haematopoietic stem cell transplant patients (HSCT) (including patients that were previously treated with CAR-T therapies, however none with CAR-T monotherapy).<br><br>Investigators chose whether to recruit adult participants into a "serology group" for the evaluation of SARS-CoV-2 spike Ab responses 28 days after V2 or a "deep immune phenotyping group" for the evaluation of T cell and humoral responses.<br><br>Participants were recruited as per the above recruitment criteria and as listed in the study protocol (supplementary).
Ethics oversight	It was approved by the UK Medicines and Healthcare Products Regulatory Agency on the 5th February 2021 and the London and Chelsea Research Ethics Committee (REC Ref:21/HRA/0489) on 12th February 2021. The protocol has subsequently been amended eight times with five substantial amendments (with ethical approvals dated 3rd March 2021, 19th April 2021, 24th December 2021, and 4th April 2022) and three non-substantial amendments protocol version dated 22nd April 2021, 14th July 2021, and 10th September 2021).

Note that full information on the approval of the study protocol must also be provided in the manuscript.

Field-specific reporting

Please select the one below that is the best fit for your research. If you are not sure, read the appropriate sections before making your selection.

☒ Life sciences ☐ Behavioural & social sciences ☐ Ecological, evolutionary & environmental sciences

For a reference copy of the document with all sections, see nature.com/documents/nr-reporting-summary-flat.pdf

Life sciences study design

All studies must disclose on these points even when the disclosure is negative.

Sample size	The sample size was based on the numbers of recruits that were estimated to have been able to recruit within the short space of time required. An effect size calculation, based on a t-test was done to provide information as to whether the number of recruits would be sufficient
Data exclusions	Missing data were excluded from the analyses. No other data were omitted.
Replication	Analyses were independently reproduced by a separate statistician where appropriate (replicated one time). Results were compared and no discrepancies were found. For Roche anti-SARS-CoV-2 S Elecsys assays performed at UKHSA, and T-SPOT assays performed at Oxford Immunotec samples were tested in singlets, in highly standardized and reproducible assays. For ELISpot assays done to assess variant of concern cross-reactivity, they were run in duplicate.
Randomization	This was an observational study and as such randomization was not required. Patients were selected based on inclusion criteria above, and therefore were not randomized.

Blinding

Blinding was not required for this study as recruits were receiving whichever COVID-19 vaccine as available as part of the UK COVID-19 vaccine roll-out. However, as recruitment was being carried out at numerous different sites, results relating to patient responses to the vaccine were not shared until after the adult cohort recruitment had been closed.

Reporting for specific materials, systems and methods

We require information from authors about some types of materials, experimental systems and methods used in many studies. Here, indicate whether each material, system or method listed is relevant to your study. If you are not sure if a list item applies to your research, read the appropriate section before selecting a response.

Materials & experimental systems

n/a	Involved in the study	
☐	☒	Antibodies
☒	☐	Eukaryotic cell lines
☒	☐	Palaeontology and archaeology
☒	☐	Animals and other organisms
☐	☒	Clinical data
☒	☐	Dual use research of concern

Methods

n/a	Involved in the study	
☒	☐	ChIP-seq
☒	☐	Flow cytometry
☒	☐	MRI-based neuroimaging

Antibodies

Antibodies used

anti-human IFNy monoclonal antibody (mAb), clone 1-D1K, Mabtech, cat 3420-2A ; anti-human IFNy biotinylated detection antibody (clone 7-B6-1) Mabtech, cat 3420-2A. Goat anti-human IgG, HRP conjugate, Sigma, UK cat AP112P.

Validation

IFNy mAb reference: Cecil Czerkinsky, Gudrun Andersson, Hans-Peter Ekre, Lars-Åke Nilsson, Lars Klareskog, Örjan Ouchterlony, Reverse ELISPOT assay for clonal analysis of cytokine production I. Enumeration of gamma-interferon-secreting cells, Journal of Immunological Methods, Volume 110, Issue 1, 1988

Clinical data

Policy information about clinical studies
All manuscripts should comply with the ICMJE guidelines for publication of clinical research and a completed CONSORT checklist must be included with all submissions.

Clinical trial registration

ISRCTN12821688

Study protocol

Study protocol included in manuscript supplementary/appendix.

Data collection

Data were collected and stored using a REDCap v12.3.1 ©2022 Vanderbilt University. Recruitment and data occurred between 19 February 2021 and 01 October 2021

Outcomes

Primary Outcomes
Vaccine Specific Immunogenicity:
1) To measure the presence and amount of serum antibodies to discriminate IgG responses to SARS-CoV-2 from vaccination and/or infection.

2) To measure T cell responses to SARS-CoV-2 peptides following vaccination.

Measurement:
1) Anti-SARS-CoV-2 IgG Abs following vaccination will be measured using the Roche platforms by the UK Health Security Agency formerly known as Public Health England Laboratories at Porton Down. The Roche assay measures the presence and amount of serum antibodies to both the spike (S) and the nucleocapsid (N) antigens of SARS-CoV-2. This assay will enable the discrimination of IgG responses to SARS-CoV-2 that results from vaccination and/or SARS-CoV- 2 infection.

2)T cell responses to SARS-CoV-2 peptides following vaccination will be measured using the Oxford Immunotec modified T-SPOT Discovery SARS-CoV-2 assay. This IFNg ELISpot assay will provide insights into the participants' reactivity to SARS-CoV-2 S1, S2, N and membrane peptides.

Secondary Outcomes
Clinical Protection: The first symptomatic PCR-proven COVID-19 occurrence from 14 days after first dose of vaccine in participants without evidence of prior infection with SARS-CoV-2.

Measurement: Incidence of first symptomatic, PCR-proven COVID-19 occurrence from 14 days after first dose of SARSCoV-2 vaccine in participants without evidence of prior infection with SARS-CoV-2 will be recorded and indications for protection against SARS-CoV-2 infection assessed.
